# Recent advances in the synthesis of *N*-acyl sulfonamides

**DOI:** 10.1039/d5ra05157f

**Published:** 2025-09-08

**Authors:** Michelle O'Driscoll, Gangireddy Sujeevan Reddy, Timothy P. O'Sullivan

**Affiliations:** a School of Chemistry, University College Cork Cork T12 YN60 Ireland tim.osullivan@ucc.ie; b School of Pharmacy, University College Cork Cork T12 YN60 Ireland; c Analytical and Biological Chemistry Research Facility, University College Cork Cork T12 YN60 Ireland

## Abstract

The *N*-acyl sulfonamide group is widespread in pharmaceutically active compounds. This is partly due to the ability of *N*-acyl sulfonamides to act as bioisosteric equivalents of carboxylic acids. Accordingly, methods for the efficient preparation of *N*-acyl sulfonamides are of considerable interest to medicinal chemists. In this review, we summarise developments in the synthesis of this pharmaceutically relevant functional group across a broad range of methodologies.

## Abstract 2

A comprehensive review highlighting advances in the synthesis of *N*-acyl sulfonamides, including classical acylation techniques, transition-metal catalysis, and C–H functionalisation.

## Graphical abstract

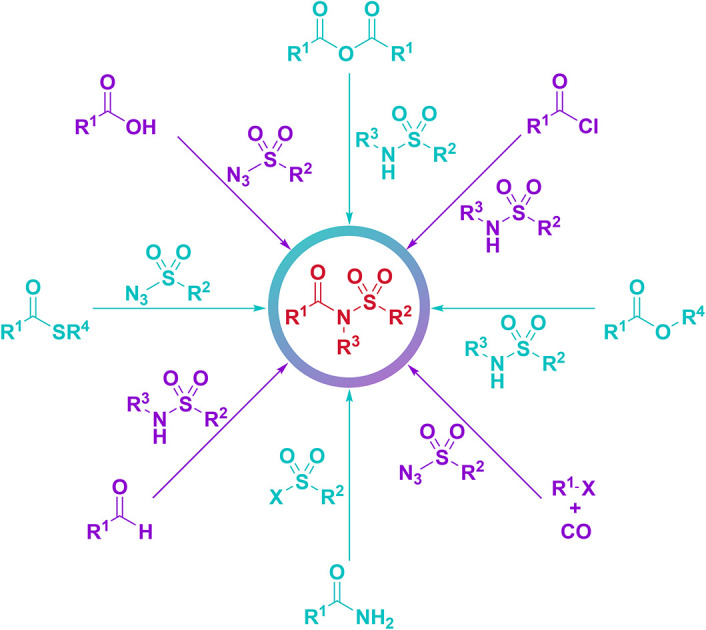

## 1. Introduction

The *N*-acyl sulfonamide is a widely explored moiety in drug discovery, primarily due to its ability to act as a bioisostere of carboxylic acids.^1–4^ Unlike sulfonamides, the acidity of *N*-acyl sulfonamides is comparable to carboxylic acids with p*K*_a_ values of 3.5–4.5.^5–7^ The comparable distances between the two sulfonyl oxygens to that of a carboxylate offers similar hydrogen bonding geometries and increased hydrogen bonding capabilities over carboxylic acids. In addition, *N*-acyl sulfonamides display increased hydrolytic and enzymatic stability compared to their carboxylic acid surrogates.^8^ The incorporation of an *N*-acyl sulfonamide permits the introduction of a wide range of structural modifiers at the carbon or sulfur atom unlike simple carboxylic acids.^9^ A judicious choice of substituents can provide access to additional regions of an enzyme receptor, allowing for control over both physicochemical properties and biological activity, a feature we have exploited in our own work on the development of novel sulfonamide-based quorum sensing inhibitors.^10,11^

**Fig. 1:**
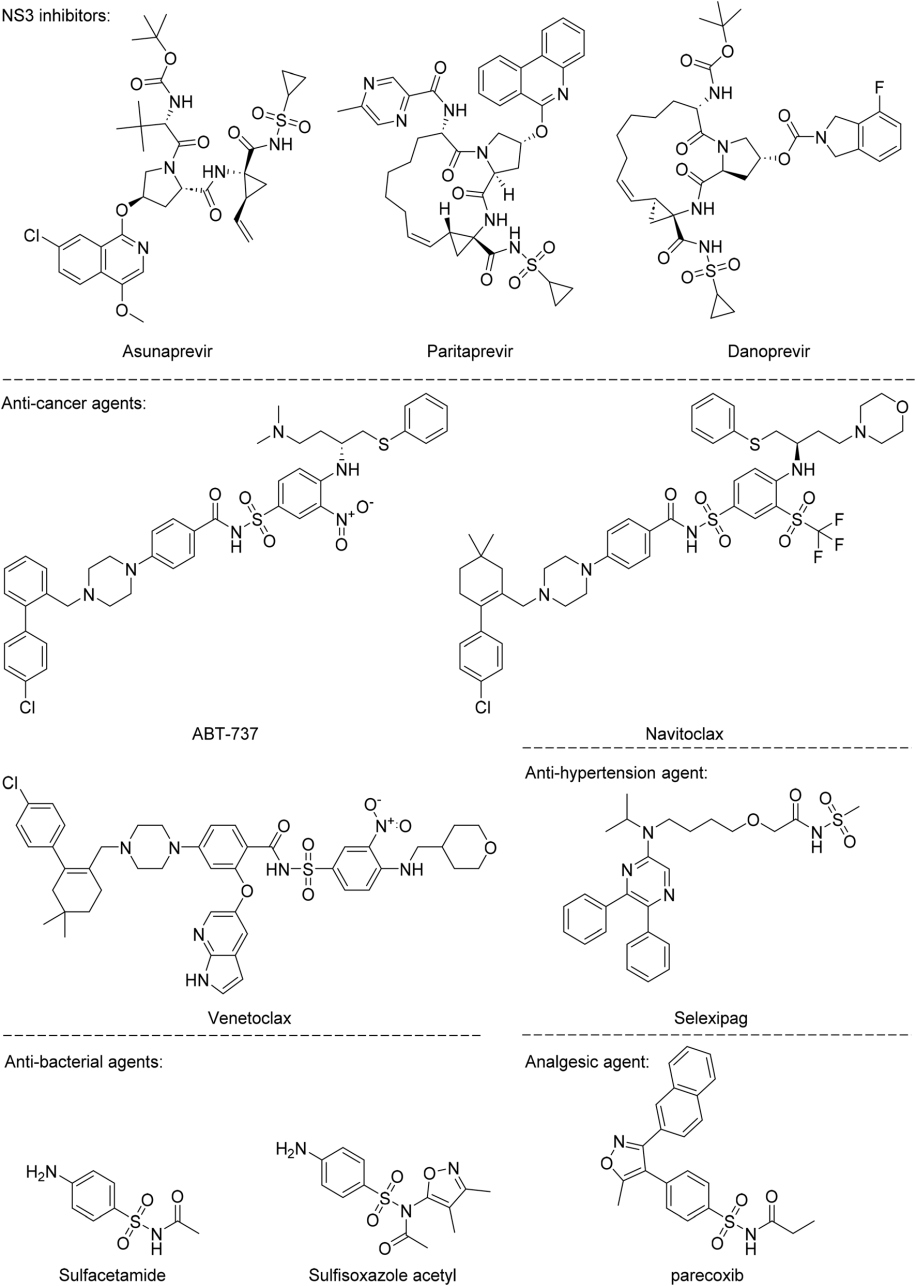
*N*-Acyl sulfonamides of clinical interest.

Therapeutics containing *N*-acyl sulfonamides are being continually developed for various indications. Examples include treatments for Alzheimer's disease,^12^ diabetes,^13^ osteoporosis,^14^ hypertension,^15,16^ cancer,^17–20^ reperfusion,^21^ asthma,^6,22^ antibacterial^23^ and antiviral infections.^24,25^ As an example, several *N*-acyl sulfonamide-based protease inhibitors of hepatitis C have been developed by Bristol-Myers Squibb,^26,27^ Abbott Laboratories^28^ and Array Biopharma^29^ and were subsequently approved by the FDA (Asunaprevir, Paritaprevir and Danoprevir). The *N*-acyl sulfonamide group is present in a variety of FDA-approved drugs and drug candidates and sample of these have been shown in Fig. 1.

*N*-Acyl sulfonamides have also found application in asymmetric organocatalysis. Ley and co-workers synthesised *N*-acyl sulfonamide-containing organocatalysts for asymmetric Mannich, nitro-Micheal and Aldol reactions.^30^ The catalyst afforded good to excellent yields and enantioselectivities and required lower catalytic loadings and shorter reaction times than their original proline lead. Likewise, Bellis and co-workers prepared and evaluated a series of prolyl sulfonamides for organocatalysed aldol reactions.^31^ 4-Benzyloxyprolyl and 4-hydroxyprolyl sulfonamides were the most promising alternatives to proline and resulted in increased enantiomeric excess of up to 20%. Additionally, acyl sulfonamides have been employed as ‘‘safety-catch” linkers in solid-phase reactions.^32^

While *N*-acyl sulfonamides are common in medicinal chemistry and organocatalysis, reviews dedicated to their synthesis are somewhat limited. Most existing reports mention this motif only briefly, typically within a broader discussion on amide bioisosteres or sulfonamide chemistry in drug design. Reviews on bioisosterism often cite *N*-acyl sulfonamides as a carboxylic acid replacement, but focus on drug design and biology.^33^ Similarly, broader surveys of sulfonamide-containing compounds usually mention *N*-acyl sulfonamides but with little in-depth coverage of their preparation.^34^ Synthetic methodology reviews may address amide bond formation or sulfonylation chemistry, but rarely integrate the two to highlight the unique challenges in accessing *N*-acyl sulfonamides.^35^

Herein, we review recent developments in the synthesis of this important functional group. This review has been subdivided based on the different reagents and approaches as follows:

(1) Acid anhydrides.

(2) Acid chlorides, bromides and benzotriazoles.

(3) Carboxylic acids and esters.

(4) Thio acids.

(5) Aldehydes and ketones.

(6) Pd-catalysed aminocarbonylations.

(7) Sulfonyl fluorides, chlorides and benzotriazoles.

(8) Alkynes.

## 2. Acid anhydrides

*N*-Acyl sulfonamides are most commonly obtained by treating a sulfonamide with an acid anhydride or acid chloride in the presence of base. A methodology to acylate aryl sulfonamides under acidic conditions was developed by GlaxoSmithKline.^36^ A catalytic amount of sulfuric acid (3 mol%) in acetonitrile was sufficient for this transformation (Table 1). Reaction of primary aliphatic sulfonamides proceeded in excellent yields (entries 1–6) as did the *N*-acylation of a secondary sulfonamide (entry 7). Trifluoromethyl and *tert*-butyl anhydrides were successfully coupled to both aliphatic and aromatic sulfonamides (entries 8–12). These conditions were also suitable for unsubstituted aromatic anhydrides (entries 13 and 14).

**Table 1:** *N*-Acylation of sulfonamides with anhydrides in the presence of sulfuric acid

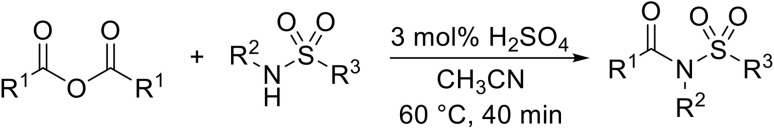				
Entry	R^1^	R^2^	R^3^	Yield
1	Me	H	Me	92%
2	Me	H	^*t*^Bu	86%
3	Me	H	Ph	95%
4	Me	H	4-MeOC_6_H_4_	91%
5	Me	H	4-O_2_NC_6_H_4_	98%
6	Me	H	2,4,6-^*i*^Pr_3_C_6_H_2_	90%
7	Me	Me	Ph	96%
8	CF_3_	H	Ph	60%
9	^*t*^Bu	H	Me	94%
10	^*t*^Bu	H	^*t*^Bu	61%
11	^*t*^Bu	H	Ph	94%
12	^*t*^Bu	H	2,4,6-^*i*^Pr_3_C_6_H_2_	90%
13	Ph	H	Me	44%
14	Ph	H	Ph	77%
15	4-F_3_CC_6_H_4_	H	Ph	n.r.
16	4-MeOC_6_H_4_	H	Ph	n.r.

Samant and co-workers found that Fe-exchanged montmorillonite was an effective catalyst for the *N*-acylation of sulfonamides.^37^ The K10–FeO catalyst was prepared by treating montmorillonite K10 clay with anhydrous iron(iii) chloride in acetonitrile. Under the optimised conditions of K10–FeO catalyst in acetonitrile at 60 °C, several primary and secondary sulfonamides were treated with acetic anhydride, benzoic anhydride and trifluoroacetic anhydride respectively (Table 2). Acetic anhydride was the superior acylating agent affording excellent yields and shorter reaction times (entries 1–6). The use of benzoic anhydride resulted in slower reaction times and moderate yields (entries 7–11). Even after 5 cycles, the activity of the catalyst was unaffected.

**Table 2:** *N*-Acylation of sulfonamides under K10–FeO catalysis

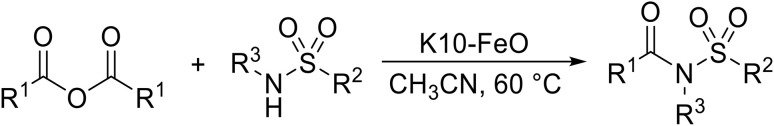					
Entry	R^1^	R^2^	R^3^	Time (min)	Yield
1	Me	Ph	H	15	92%
2	Me	4-MeC_6_H_4_	H	15	95%
3	Me	4-MeOC_6_H_4_	H	15	91%
4	Me	4-ClC_6_H_4_	H	5	98%
5	Me	Me	H	15	82%
6	Me	4-MeC_6_H_4_	Me	15	92%
7	Ph	Ph	H	120	74%
8	Ph	4-MeC_6_H_4_	H	120	72%
9	Ph	4-MeOC_6_H_4_	H	120	67%
10	Ph	4-ClC_6_H_4_	H	120	78%
11	Ph	Me	H	120	62%
12	Ph	4-MeC_6_H_4_	Me	120	—
13	CF_3_	Ph	H	30	62%
14	CF_3_	4-ClC_6_H_4_	H	30	66%

A range of Lewis acids were screened by Reddy *et al.* for the *N*-acylation of benzenesulfonamide.^38^ As the cheapest and easiest catalyst to handle, zinc chloride was subsequently selected for the *N*-acylation of a series of sulfonamide compounds under solvent-free conditions (Table 3). Reaction of benzenesulfonamide with different aliphatic anhydrides proceeded efficiently and in high yields (entries 1–5). Toluene sulfonamide was successfully converted to the *N*-acetyl and *N*-trifluoro derivatives in 97% (entry 7) and 93% yields (entry 8). The presence of an electron-withdrawing nitro group did not hinder transformation to the desired products (entries 9 and 10). Alkyl sulfonamides were likewise converted in excellent yields (entries 11 and 12). *N*-Substitution of a secondary sulfonamide with benzyl/methyl groups was successful (entries 13–17). This protocol was also expanded to the acylation of carboxylic acid substrates *via* a mixed anhydride (see Scheme 10).

**Table 3:** *N*-Acylation of sulfonamides under zinc chloride catalysis

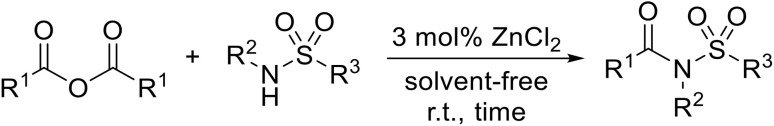					
Entry	R^1^	R^2^	R^3^	Time (min)	Yield
1	Me	H	Ph	2	97%
2	Et	H	Ph	4	96%
3	^*n*^Pr	H	Ph	7	95%
4	^*t*^Bu	H	Ph	60	90%
5	CF_3_	H	Ph	10	94%
6	Ph	H	Ph	240	88%
7	Me	H	4-MeC_6_H_4_	2	97%
8	CF_3_	H	4-MeC_6_H_4_	10	93%
9	Me	H	4-O_2_NC_6_H_4_	3	95%
10	^*n*^Pr	H	4-O_2_NC_6_H_4_	5	94%
11	Me	H	Me	2	98%
12	^*n*^Pr	H	Me	3	96%
13	Me	Bn	4-MeC_6_H_4_	5	94%
14	Et	Bn	4-MeC_6_H_4_	8	93%
15	Me	Bn	Me	5	95%
16	Et	Bn	Me	7	92%
17	Me	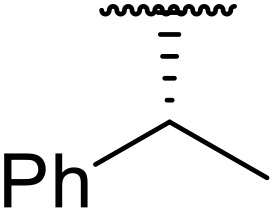	Me	2	96%

The catalytic effects of magnetic Fe_3_O_4_ diatomite earth (Fe_3_O_4_@DE) was investigated by Kowsari and co-workers (Table 4).^39^ Fe_3_O_4_@DE easily outperformed other iron-based heterogeneous catalysts and equalled or surpassed acetic acid (entries 1–10). Notably, Fe_3_O_4_@DE is effective under solvent-free conditions, can be easily recovered by application of a magnetic field and withstands five reaction cycles without loss of activity. This catalyst system has also been successfully applied to benzoyl chloride-mediated *N*-acylations (see Scheme 3).

**Table 4:** *N*-Acylation of sulfonamides using acetic anhydride and Fe_3_O_4_@DE

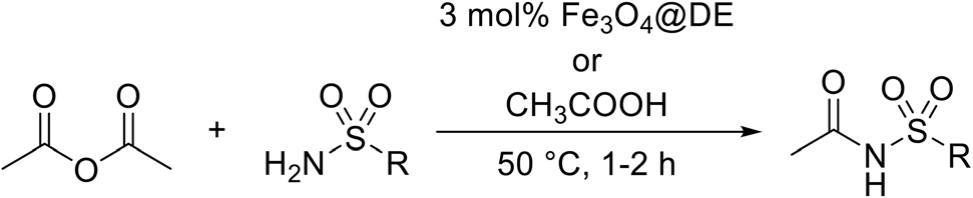			
Entry	R	Yield	
		Fe_3_O_4_@DE	CH_3_COOH
1	4-AcHNC_6_H_4_	93%	95%
2	Ph	95%	90%
3	4-ClC_6_H_4_	95%	85%
4	4-MeC_6_H_4_	93%	85%
5	4-MeOC_6_H_4_	95%	80%
6	3-O_2_NC_6_H_4_	95%	85%
7	3-HO_2_CC_6_H_4_	95%	75%
8	4-BrC_6_H_4_	90%	85%
9	4-O_2_NC_6_H_4_	88%	80%
10	Me	97%	95%

Bougheloum and co-workers described the use of H_6_P_2_W_18_O_62_, a Wells–Dawson type heteropolyacid catalyst, for the conversion of sulfamides to *N*-acyl sulfamides (Table 5).^40^ Addition of 1 mmol% H_6_P_2_W_18_O_62_ in acetonitrile efficiently catalysed the conversion of aliphatic and aromatic sulfamides to the corresponding *N*-acyl sulfamide in excellent yields (entries 1–5).

**Table 5:** *N*-Acylation of sulfamides under H_6_P_2_W_18_O_62_ catalysis

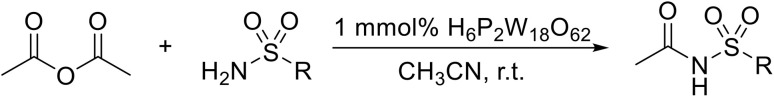			
Entry	R	Time (min)	Yield
1	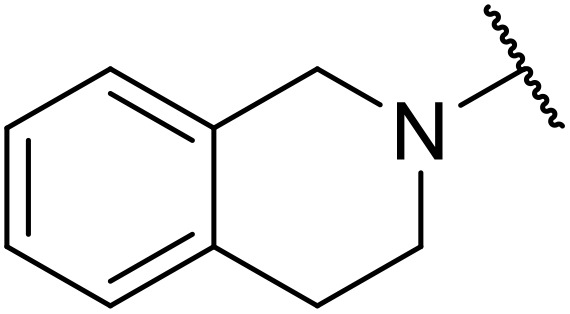	15	95%
2	^*n*^PrNH	22	92%
3	^*t*^BuNH	20	93%
4	PhCH_2_NH	25	94%
5	3-FC_6_H_4_NH	20	94%

Bougheloum adapted this chemistry into a green synthesis of *N*-acylsulfonamides.^41^ A cesium salt of the Wells–Dawson heteropolyacid, which offered good recoverability, was assessed using several acid anhydrides in water (Table 6). Under these conditions, acetic anhydride reacted with sulfonamides in good to excellent yields and more rapidly than acetyl chloride (entries 1–9). Cyclic anhydrides, including succinic anhydride (entries 10–17), maleic anhydride (entry 19), 2,3-dichloro maleic anhydride (entry 20), glutaric anhydride (entry 21) and phthalic anhydride (entry 22), were similarly compatible with this protocol.

**Table 6:** *N*-Acylation of primary sulfonamides in water using Cs_5_HP_2_W_18_O_62_

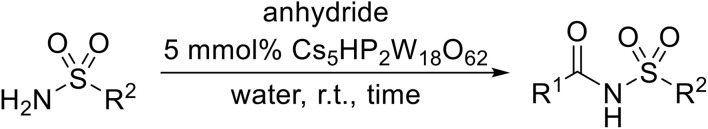					
Entry	Anhydride	R^1^	R^2^	Time (min)	Yield
1	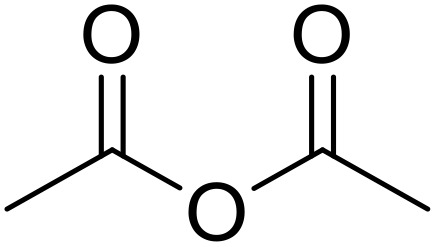	Me	^*n*^BuNH	20	91%
2	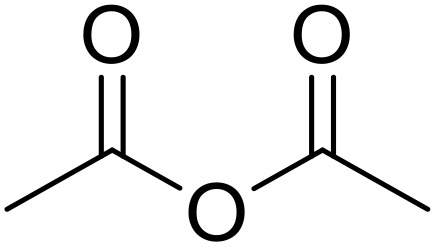	Me	^*t*^BuNH	20	92%
3	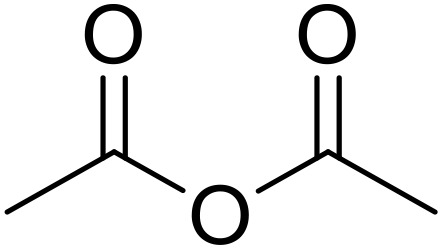	Me	Cl(CH_2_)_2_NH	30	79%
4	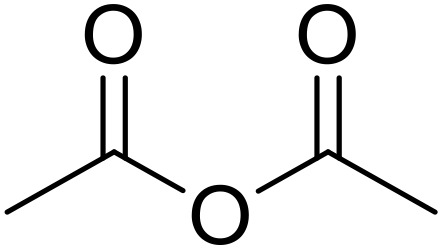	Me	PhNH	25	92%
5	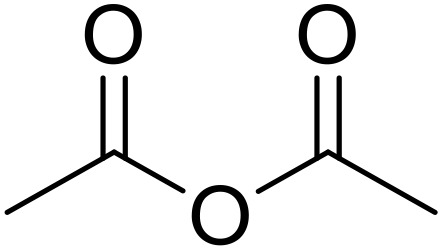	Me	PhCH_2_NH	25	85%
6	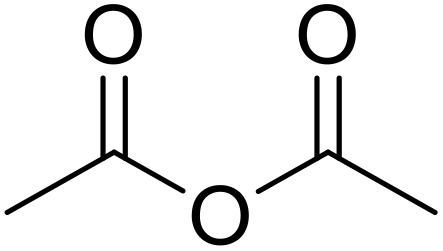	Me	3-FC_6_H_4_NH	30	76%
7	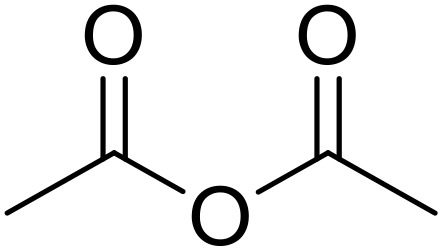	Me	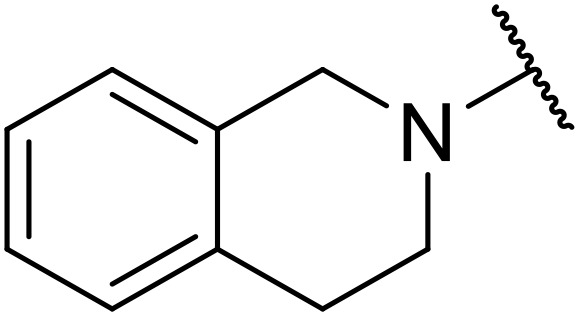	30	88%
8	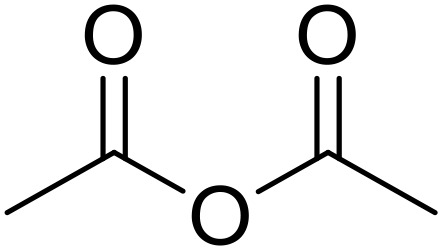	Me	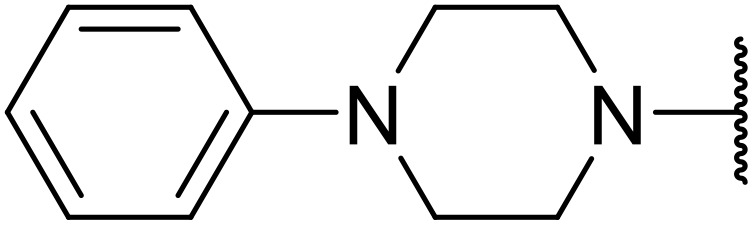	30	85%
9	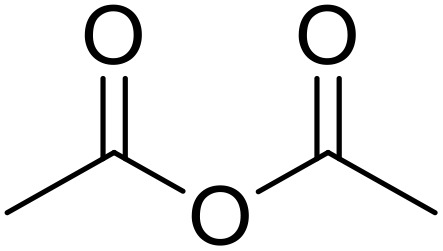	Me	4-MeC_6_H_4_	20	92%
10	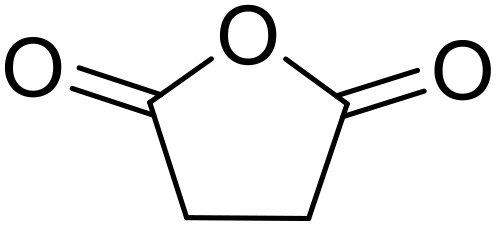	HO_2_CCH_2_CH_2_	^*n*^BuNH	20	89%
11	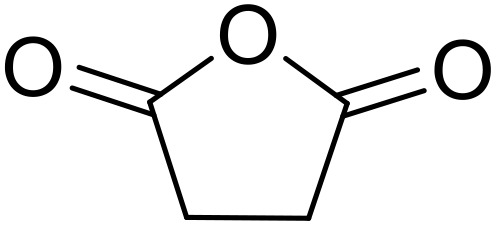	HO_2_CCH_2_CH_2_	^*t*^BuNH	20	90%
12	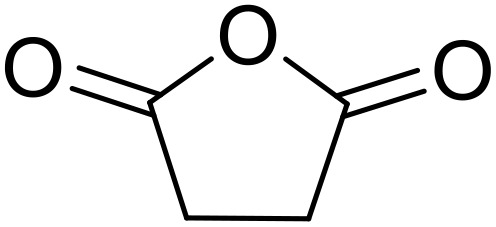	HO_2_CCH_2_CH_2_	PhNH	25	88%
13	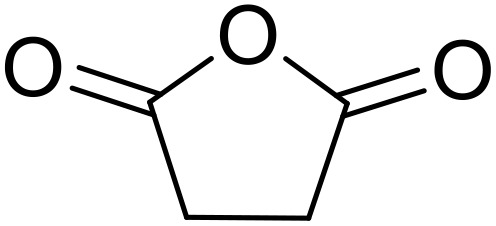	HO_2_CCH_2_CH_2_	PhCH_2_NH	30	86%
14	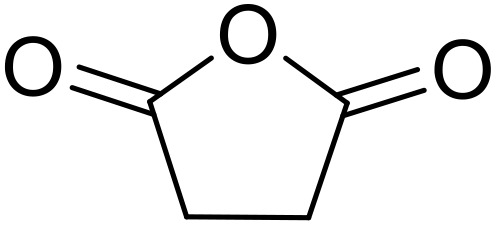	HO_2_CCH_2_CH_2_	3-FC_6_H_4_NH	30	74%
15	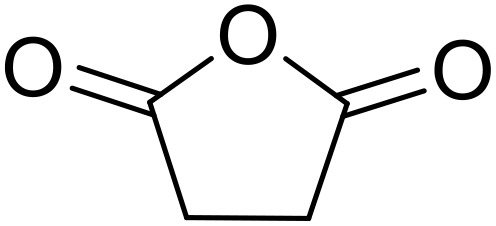	HO_2_CCH_2_CH_2_	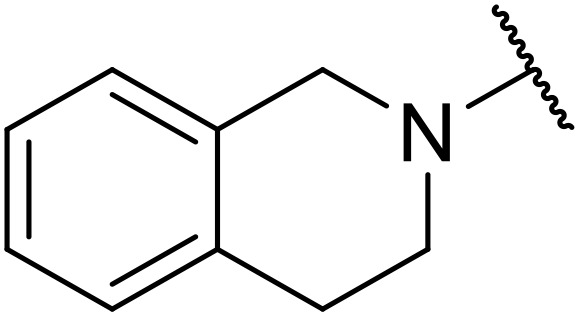	30	85%
16	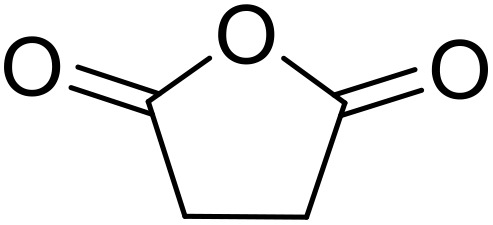	HO_2_CCH_2_CH_2_	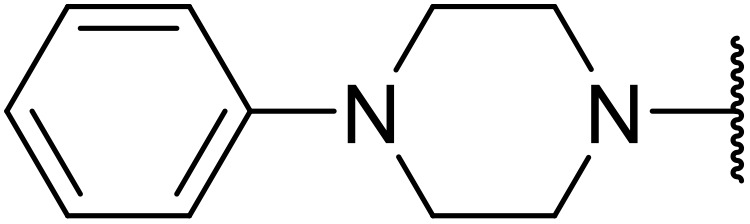	30	84%
17	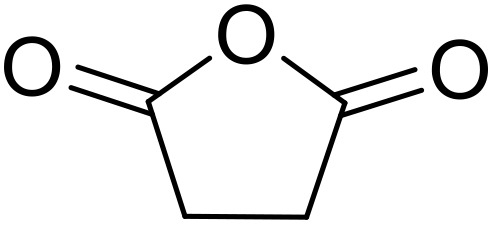	HO_2_CCH_2_CH_2_	4-MeC_6_H_4_	35	90%
18	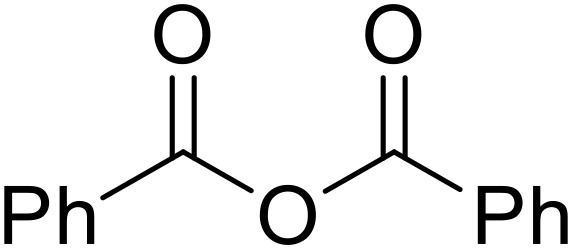	Ph	4-MeC_6_H_4_	90	65%
19	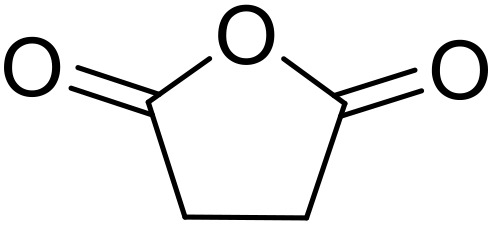	HO_2_CCH <svg xmlns="http://www.w3.org/2000/svg" version="1.0" width="13.200000pt" height="16.000000pt" viewBox="0 0 13.200000 16.000000" preserveAspectRatio="xMidYMid meet"><metadata> Created by potrace 1.16, written by Peter Selinger 2001-2019 </metadata><g transform="translate(1.000000,15.000000) scale(0.017500,-0.017500)" fill="currentColor" stroke="none"><path d="M0 440 l0 -40 320 0 320 0 0 40 0 40 -320 0 -320 0 0 -40z M0 280 l0 -40 320 0 320 0 0 40 0 40 -320 0 -320 0 0 -40z"/></g></svg> CH	4-MeC_6_H_4_	35	79%
20	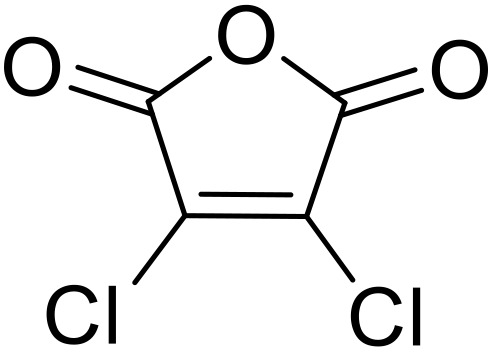	HO_2_CC(Cl)C(Cl)	4-MeC_6_H_4_	45	75%
21	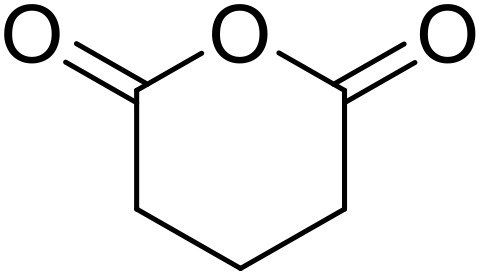	HO_2_C(CH_2_)_2_CH_2_	4-MeC_6_H_4_	45	80%
22	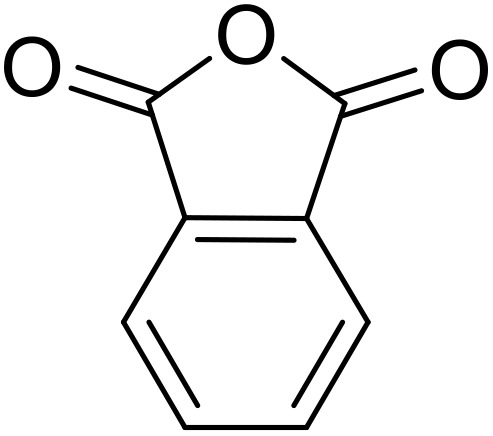	2-HO_2_CC_6_H_4_	4-MeC_6_H_4_	50	55%

**Scheme 1:**
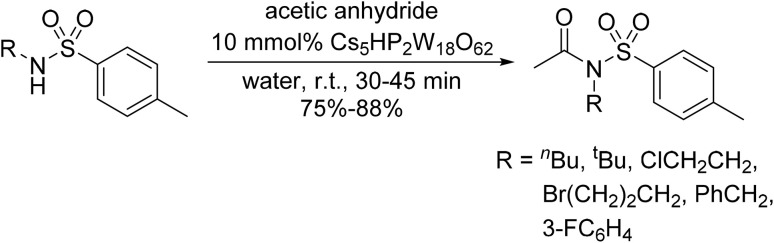
*N*-Acylation of secondary sulfonamides in water using Cs_5_HP_2_W_18_O_62_.

The acylation of secondary sulfonamides with acetic anhydride was effectively catalysed by the same catalyst in water, furnishing the target *N*-acyl sulfonamides in 75–88% yields (Scheme 1). In general, the conversion of primary sulfonamides (Table 6) was faster and higher yielding than their secondary sulfonamide counterparts (Scheme 1). By increasing the catalyst loading from 5 mmol% to 10 mmol%, comparable yields to the primary sulfonamides were achieved.

Ghazviniyan and co-workers established a procedure for obtaining *N*-acyl sulfonamides under functionalised graphene oxide@ZIF-90-supported sulfuric acid (GO@ZIF-90@NCH_2_CH_2_NH_3_^+^HSO_4_^−^ or GZAH) catalysis.^42^ A small number of sulfonamides were successfully acylated with acetic anhydride in acetonitrile at 50 °C in excellent yields of 85–95% (Table 7). The GZAH catalyst could be recycled in five sequential reactions without significant loss of efficacy.

**Table 7:** *N*-Acylation of sulfonamides under GZAH catalysis

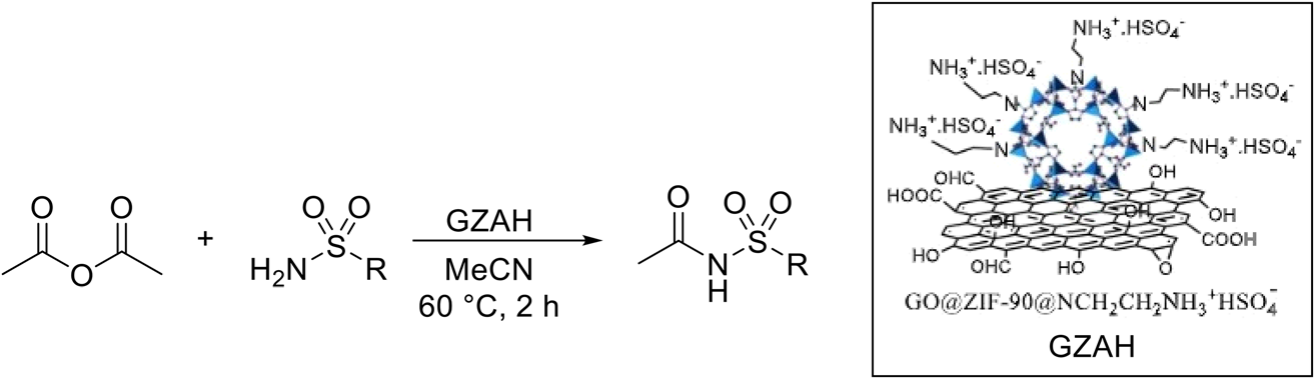		
Entry	R	Yield
1	Ph	95%
2	4-MeOC_6_H_4_	93%
3	4-MeC_6_H_4_	85%
4	4-ClC_6_H_4_	85%
10	4-BrC_6_H_4_	92%

Bouasla and colleagues demonstrated how ultrasonic irradiation could be exploited for the preparation *N*-acyl sulfonamides under green conditions (Table 8).^43^ Several sulfonamides were acetylated using an ultrasonic bath at 40 kHz, without the requirement of a catalyst or solvent. The products were obtained in 60–96% yields over 15–60 minutes (entries 1–10). The accelerating effect of ultrasound likely results from cavitation, whereby bubbles implode, generating sites of high temperatures and pressures in the reaction mixture, thus driving the reaction forward.

**Table 8:** Catalyst- and solvent-free acylation under ultrasonic irradiation

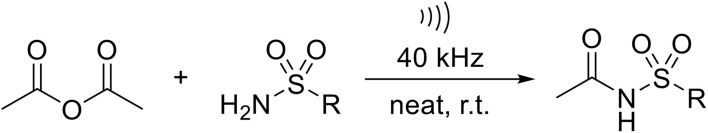			
Entry	R	Time (min)	Yield
1	Cy–NH	30	94%
2	^*n*^PrNH	15	90%
3	^*t*^BuNH	15	95%
4	PhCH_2_NH	30	96%
5	3-FC_6_H_4_NH	30	93%
6	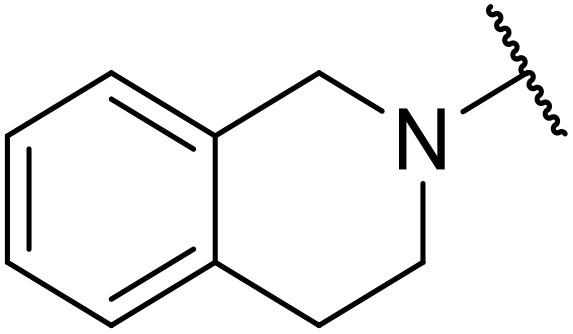	40	90%
7	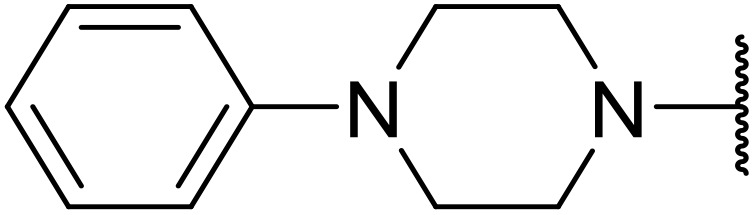	60	60%
8	4-MeOC_6_H_4_NH	35	80%
9	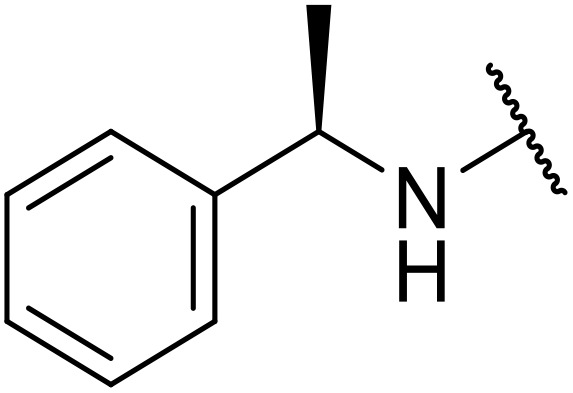	20	85%
10	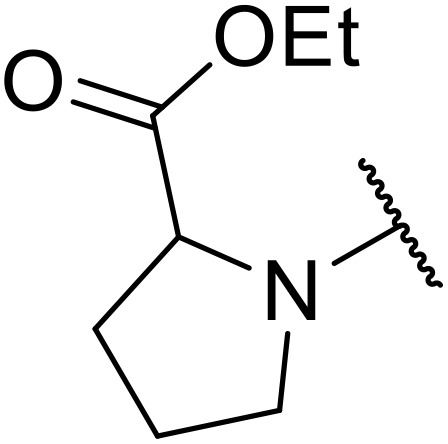	30	83%

Atropoisomerism is form of chirality which results from restricted rotation around a bond axis.^44^ Ong and co-workers developed an isothiourea-catalysed atroposelective *N*-acylation of sulfonamides in good to high enantiopurities (Table 9).^45^ Three different isothiourea catalysts were screened, with commercially available isothiourea (*S*)-homobenzotetramisole or (*S*)-HBTM in cyclopentyl methyl ether proving optimal. Electron-rich and electron-poor anhydrides performed equally well (entries 2 and 3). Bicyclic (entry 4), aliphatic (entries 5 and 9) and heteroaromatic (entry 6) unsaturated anhydrides were equally well tolerated, achieving yields of 73–93% and enantioselectivities of 65–99%.

**Table 9:** Isothiourea-catalysed atroposelective *N*-acylation with various anhydrides

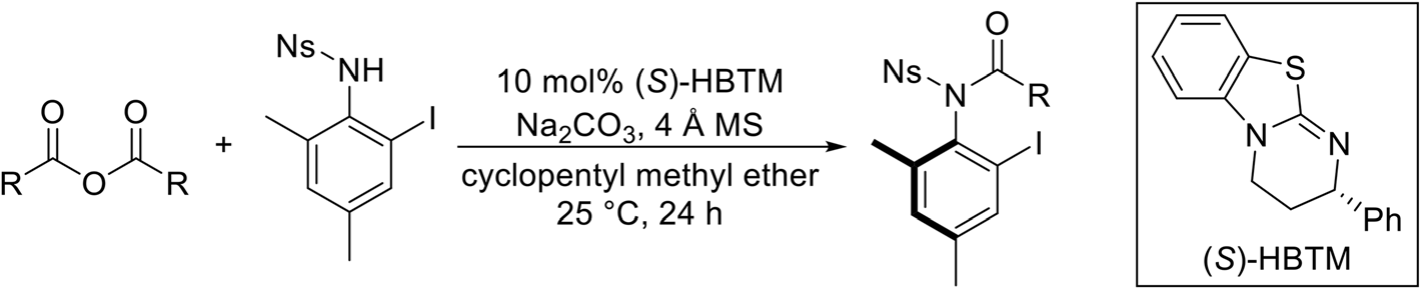			
Entry	R	Yield	ee
1	(*E*)-PhCH=CH	73%	85%
2	(*E*)-2-MeOC_6_H_4_CH=CH	59%^a^	82%
3	(*E*)-4-FC_6_H_4_CH=CH	78%	85%
4	(*E*)-1-NapthylC_6_H_4_CH=CH	88%	82%
5	(*E*)-^*n*^PrCH=CH	90%	65%
6	(*E*)-2-FurylCH=CH	93%	72%
7	Ph	73%^b^	82%
8	PhCH_2_CH_2_	86%	80%
9	Et	86%^a^	88%

a 20 mol% (*S*)-HBTM used.

b 96 h reaction time.

The same authors next investigated the scope of the sulfonamide substrates (Table 10). Replacing the *para*-methyl for a phenyl, chloride, bromide or iodide substituent gave similar yields but reduced enantioselectivities of 71–79% (entries 1–4). An *ortho*-methyl was required for good enantioselectivity as replacing an *ortho*-methyl with an *ortho*-ethyl was associated with a significant reduction in enantioselectivity to 49% (entry 5). Swapping the *ortho*-iodo for an *ortho*-bromo was beneficial, affording the acylated products in up to 94% yield and achieving enantioselectivities of 81–84% (entries 6 and 7). By contrast, substituting the iodine with a chlorine did not furnish the desired atropoisomer, likely due to the chlorine atom being too small to provide an adequate rotation barrier along the C–N axis (entry 11). Replacing the 4-nosyl protecting group with either a 3-nosyl (entry 8), 4-chlorophenyl (entry 9) or methyl (entry 10) produced comparable yields and stereoselectivities. Finally, no atropoisomerism was observed in the absence of an *ortho*-alkyl substituent (entries 12 and 13).

**Table 10:** Isothiourea-catalysed atroposelective *N*-acylation of various sulfonamides

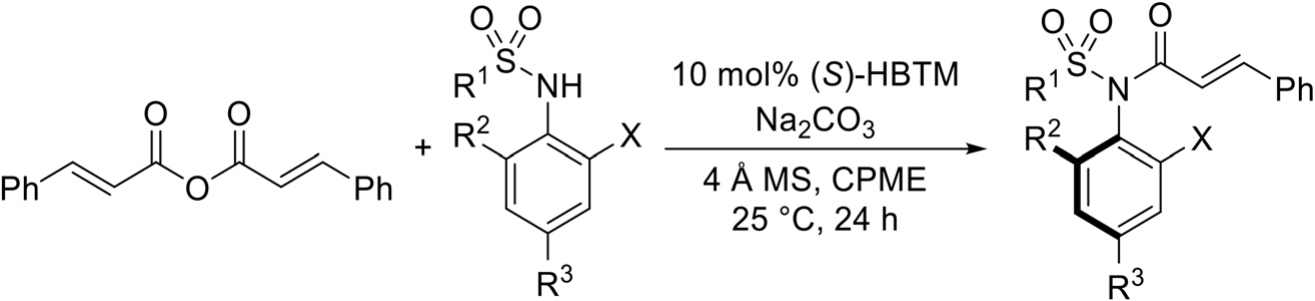						
Entry	R^1^	R^2^	R^3^	X	Yield	ee
1	4-O_2_NC_6_H_4_	Me	Ph	I	83%	79%
2	4-O_2_NC_6_H_4_	Me	Cl	I	81%	73%
3	4-O_2_NC_6_H_4_	Me	Br	I	78%	72%
4	4-O_2_NC_6_H_4_	Me	I	I	63%	71%
5	4-O_2_NC_6_H_4_	Et	I	I	52%	49%
6	4-O_2_NC_6_H_4_	Me	Me	Br	94%	84%
7	4-O_2_NC_6_H_4_	Me	H	Br	73%	81%
8	3-O_2_NC_6_H_4_	Me	Me	I	82%	88%
9	4-ClC_6_H_4_	Me	Me	I	83%	78%^a^
10	Me	Me	Me	I	78%	84%
11	4-O_2_NC_6_H_4_	Me	Me	Cl	—	N.A.^b^
12	4-O_2_NC_6_H_4_	H	H	I	—	N.A.^b^
13	4-O_2_NC_6_H_4_	H	H	I	—	N.A.^b^

a 20 mol% (*S*)-HBTM used.

b N.A.: no atropoisomerism.

In a series of papers, Massah and co-workers investigated several catalysts (P_2_O_5_/SiO_2_,^46^ H_3_PO_4_/SiO_2_,^47^ SiO_2_–Cl,^48^ Bi(OTf)_3_,^49^ BiCl_3_,^49^ Al(HSO_4_)_3_ ^50^ and Zr(HSO_4_)_4_)^50^ for the *N*-acylation of sulfonamides using common acid anhydrides under both homogeneous and heterogeneous conditions (Table 11). Acetylation catalysed by Bi(OTf)_3_ at 5 mol% loading was exceptionally rapid, yielding products in as little as 1–2 minutes in yields of up to 96% (entry 11). Methyl, benzene and *ortho*-methylbenzene sulfonamides were *N*-acylated with methanoic, ethanoic, *n*-propanoic, *i*-propanoic, *n*-butanoic, *n*-pentanoic, benzoic and 4-nitrobenzoic anhydrides (entries 1–23). Several of these reactions were also carried out using acid chlorides (see Acid chlorides section).

**Table 11:** Comparison of the effect of selected catalysts on the *N*-acylation of primary sulfonamides

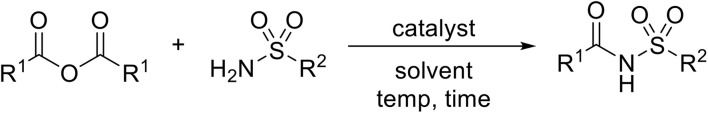																
Entry	R^1^	R^2^	P_2_O_5_/SiO_2_^46^		H_3_PO_4_/SiO_2_ ^47^		SiO_2_–Cl^48^		Bi(OTf)_3_ ^49^		BiCl_3_ ^49^		Al(HSO_4_)_3_ ^50^		Zr(HSO_4_)_4_ ^50^	
			Time (min)	Yield^a^	Time (min)	Yield^b^	Time (min)	Yield^c^	Time (min)	Yield^d^	Time (min)	Yield^e^	Time (min)	Yield^f^	Time (min)	Yield^g^
1	Me	Me	40	85%	60	80%	72	88%	2	90%	10	90%	5	86%	7	85%
2	Et	Me	15	80%	20	82%	18	85%	2	91%	12	88%				
3	^*n*^Pr	Me	25	75%	20	80%	24	80%	1	91%	10	88%				
4	^*i*^Pr	Me	25	96%	20	96%	15	95%	2	91%	13	91%	5	89%	7	85%
5	^*n*^Bu	Me			30	80%			5	90%	13	90%				
6	^*n*^Pent	Me											5	89%	7	85%
7	Ph	Me	180	82%			300	85%	30	91%	60	87%				
8	Me	Ph	45	85%	55	80%	90	80%	2	94%	15	90%	7	91%	15	90%
9	Et	Ph	25	85%	25	85%	30	85%	2	92%	17	89%				
10	^*n*^Pr	Ph	30	80%	30	80%	36	90%	2	95%	17	90%	7	88%	15	91%
11	^*i*^Pr	Ph	35	95%	25	95%	24	95%	1	96%	20	92%	7	85%	15	81%
12	^*n*^Bu	Ph			35	80%			3	92%	20	90%				
13	Ph	Ph	105	90%			360	80%	35	90%	70	85%	25	72%	70	70%
14	4-O_2_NC_6_H_4_	Ph							600	10%	720	10%				
15	Me	4-MeC_6_H_4_	60	75%	90	95%	72	74%	2	94%	20	90%	5	86%	7	90%
16	Et	4-MeC_6_H_4_	45	83%	30	80%	36	85%	2	93%	20	92%	5	88%	7	89%
17	^*n*^Pr	4-MeC_6_H_4_	35	78%	35	85%	36	93%	3	90%	20	90%				
18	^*i*^Pr	4-MeC_6_H_4_	30	97%	25	97%	30	95%	1	91%	20	89%				
19	^*n*^Bu	4-MeC_6_H_4_							3	96%	20	88%	5	88%	7	88%
20	^*n*^Pent	4-MeC_6_H_4_											5	89%	7	85%
21	Ph	4-MeC_6_H_4_	160	80%			420	60%	30	88%	80	82%				
22	Et	4-O_2_NC_6_H_4_	80	78%												
23	^*n*^Pr	4-O_2_NC_6_H_4_	90	75%												

a Sulfonamide: 2 mmol; acid anhydride: 2 mmol; catalyst: 0.35 g; CH_2_Cl_2_: 5 mL; reflux.

b Sulfonamide: 1 mmol; acid anhydride: 2 mmol (in two portions); H_3_PO_4_/SiO_2_: 0.3 g; *n*-hexane: 5 mL; reflux.

c Sulfonamide: 1 mmol; acid anhydride: 2 mmol; SiO_2_–Cl: 0.3 g; *n*-hexane: 5 mmol; reflux.

d Sulfonamide: 1 mmol; acid anhydride: 2 mmol; Bi(OTf)_3_: 5 mol%; CHCl_3_: 5 mL; reflux.

e Sulfonamide: 1 mmol; acid anhydride: 2 mmol; BiCl_3_: 10 mol%; CHCl_3_: 5 mL; reflux.

f Sulfonamide: 1 mmol; acid anhydride: 1.1–1.5 mmol; Al(HSO_4_)_3_: 5 mol%; CH_2_Cl_2_: 5 mL; r.t.

g Sulfonamide: 1 mmol; acid anhydride: 1.1–1.5 mmol; Zr(HSO_4_)_4_: 5 mol%; CH_2_Cl_2_: 5 mL; r.t.

This methodology was also compatible with secondary sulfonamides (Table 12).^50^ Increasing the steric bulk on the nitrogen substituent from ^*i*^Pr (entry 1) to ^*n*^Bu (entry 5) did not impact upon reaction efficiency. The desired products were recovered in as little as 5 minutes and in yields of up to 96% (entries 1–8). The incorporation of a phenyl ring was associated with slower reaction rates and reduced yields of 70–75% (entries 9–13).

**Table 12:** *N*-Acylation of secondary sulfonamides under metal hydrogen sulfate catalysis

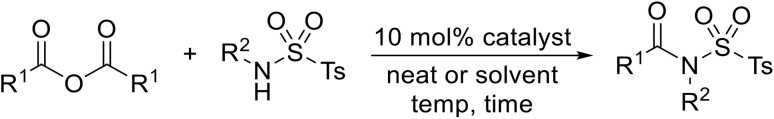										
Entry	R^1^	R^2^	Al(HSO_4_)_3_				Zr(HSO_4_)_4_			
			Solvent-free		CH_2_Cl_2_		Solvent-free		CHCl_3_	
			Time (min)	Yield	Time (min)	Yield	Time (min)	Yield	Time (min)	Yield
1	Me	^*n*^Pr	5	93%	7	90%	10	82%	15	80%
2	^*n*^Pr	^*n*^Pr	5	95%	7	94%	10	80%	15	85%
3	^*n*^Bu	^*n*^Pr	5	96%	7	93%	10	78%	15	82%
4	^*i*^Pr	^*n*^Pr	5	85%	7	83%	10	71%	15	70%
5	Me	^*n*^Bu	5	90%	7	88%	10	82%	15	78%
6	Et	^*n*^Bu	5	92%	7	89%	10	80%	15	81%
7	^*n*^Pr	^*n*^Bu	5	92%	7	90%	10	78%	15	80%
8	^*n*^Bu	^*n*^Bu	5	89%	7	85%	10	85%	15	82%
9	Et	Ph	15	75%	20	75%	30	72%	45	72%
10	^*n*^Pr	Ph	15	75%	20	71%	30	70%	45	70%
11	^*n*^Bu	Ph	15	74%	20	70%	30	70%	45	70%
12	^*i*^Pr	Ph	15	72%	20	70%	30	71%	45	70%
13	^*n*^Pent	Ph	15	70%	20	70%	30	71%	45	70%

**Scheme 2:**
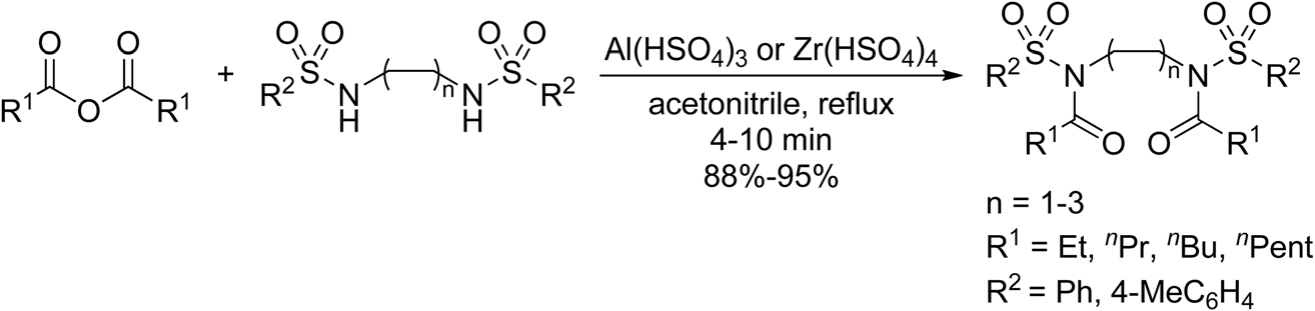
*N*-Acylation of bis-sulfonamides under metal hydrogen sulfate catalysis.

Massah also reported the first examples of the preparation of bis-*N*-acyl sulfonamides (Scheme 2).^50^ Several bis-sulfonamides were initially prepared *via* sulfonylation of an appropriate diamine. *N*-Acylation of these substrates was subsequently conducted in the presence of Al(HSO_4_)_3_ or Zr(HSO_4_)_4_ in refluxing acetonitrile. Excellent yields (88–95%) of bis-*N*-acyl sulfonamides of varying chain lengths and increasing steric bulk were obtained in less than 10 minutes.

## 3. Acid chlorides, bromides and benzotriazoles

A one-pot sequential sulfonylation and acylation of amines in the presence of ZnO was described by Tamaddon and colleagues (Table 13).^51^ An initial one hour stir time of the sulfonyl chloride, amine and 20 mol% ZnO, followed by the addition of the acid chloride, generated the *N*-acyl sulfonamides in excellent yields (entries 1–10). Type I ZnO nanoparticles were found to outperform both ZnO and type II ZnO nanoparticles, as well as the recovered ZnO catalyst (entries 3 and 6–8).

**Table 13:** One-pot sequential sulfonylation/acylation

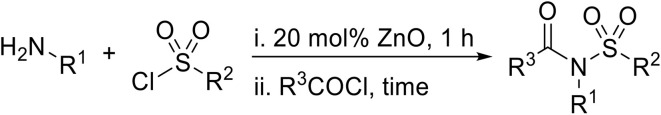					
Entry	R^1^	R^2^	R^3^	Time (h)	Yield
1	H	Me	Me	0.5	95%
2	H	Me	Ph	0.5	92%
3	H	Ph	Ph	1	90%, 91%^a^
4	H	4-MeC_6_H_4_	Me	0.5	92%
5	H	4-MeC_6_H_4_	Ph	0.5	90%
6	Ph	Me	Ph	0.75	89%, 91%^a^
7	Ph	Ph	Me	0.75	93%, 93%^a^
8	Ph	4-MeC_6_H_4_	Me	0.5	93%, 91%^a^
9	Bu	4-MeC_6_H_4_	Me	0.5	92%
10	Bu	4-MeC_6_H_4_	Ph	1	93%

a 0.3 mmol (30 mol%) of type I nanoZnO used.

**Scheme 3:**
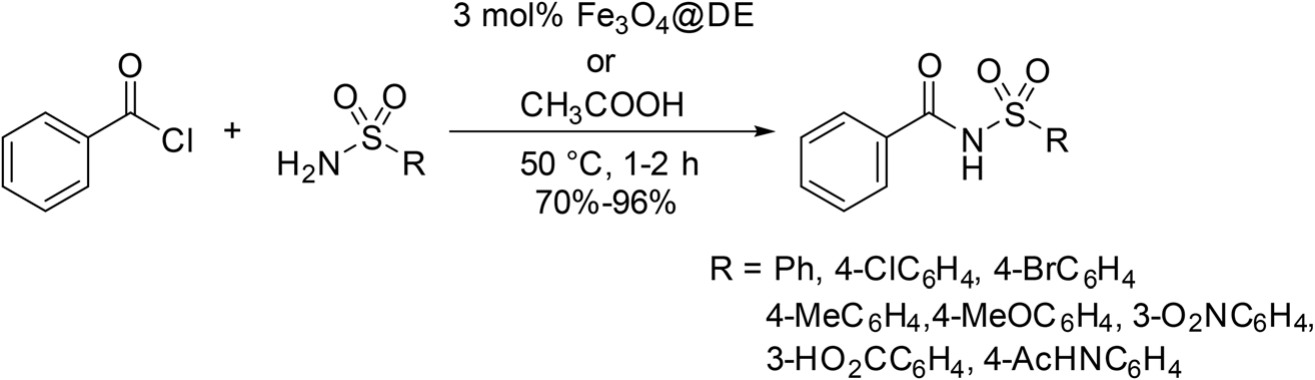
*N*-Acylation of sulfonamides under Fe_3_O_4_@DE catalysis.

Fe_3_O_4_@DE, developed by Kowsari as an *N*-acylation catalyst for anhydrides (see Table 4), is equally applicable to acid chlorides (Scheme 3).^39^ In most cases, benzoyl chloride was a better coupling partner, affording higher yields than acetic anhydride in the presence of either Fe_3_O_4_@DE or acetic acid.

Williams explored the utility of iodide salts as activating agents for acid chlorides.^52^ Potassium iodide in acetonitrile at 60 mol% was optimal and proved superior to both lithium and sodium iodide. Various sulfonamides and acid chlorides were employed to probe the reaction scope with yields of 54–97% being recorded (Table 14). Partial racemisation was observed when (*S*)-2-phenylpropanoyl chloride was employed, suggesting that the reaction does not proceed solely *via* a ketene intermediate (entry 12). The mechanism likely involves conversion of the acid chloride to the more reactive acid iodide, which is characterised by greater polarizability and reduced overlapping of the iodine's lone pairs with 
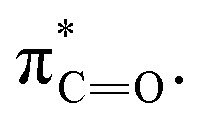


**Table 14:** Use of potassium iodine to generate acid iodides

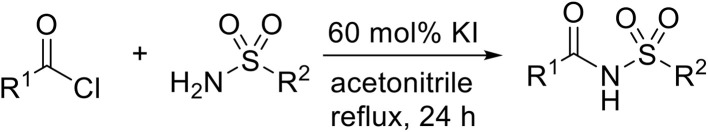			
Entry	R^1^	R^2^	Yield
1	Ph	4-MeC_6_H_4_	89%
2	^*n*^Bu	4-MeC_6_H_4_	88%
3	4-BrC_6_H_4_	4-MeC_6_H_4_	67%
4	2-MeC_6_H_4_	4-MeC_6_H_4_	92%
5	PhCH_2_CH_2_	4-MeC_6_H_4_	91%
6	^*t*^Bu	4-MeC_6_H_4_	80%
7	4-MeOC_6_H_4_	4-MeC_6_H_4_	97%
8	Ph	Ph	96%
9	Ph	4-MeOC_6_H_4_	83%
10	Ph	2,4,6-Me_3_C_6_H_2_	73%
11	Ph	Me	83%
12	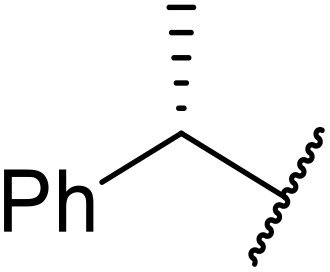	4-MeC_6_H_4_	54% (80% ee)

Massah expanded his study of anhydride catalysts (see Table 11), and found that acid chlorides were even more reactive in the presence of P_2_O_5_/SiO_2_, Bi(OTf)_3_ or BiCl_3_ (Table 15). Equimolar equivalents of acid chloride proved sufficient for smooth conversion whereas at least two equivalents of the anhydride were required. Bi(OTf)_3_ produced remarkably fast and efficient reactions, generating products in 1–45 min (entries 1–9). Contrastingly, reactions were generally slower (35–240 min) and lower yielding (60–85%) with the H_3_PO_4_/SiO_2_ system (entries 1–9). Secondary sulfonamides underwent successful acylation in the presence of metal hydrogen sulfate catalysts (entries 10–12).

**Table 15:** *N*-Acylation of sulfonamides in the presence of different acid catalysts

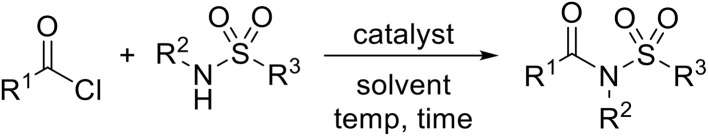																	
Entry	R^1^	R^2^	R^3^	P_2_O_5_/SiO_2_ ^46^		H_3_PO_4_/SiO_2_ ^47^		SiO_2_–Cl^48^		Bi(OTf)_3_ ^49^		BiCl_3_ ^49^		Al(HSO_4_)_3_ ^50^		Zr(HSO_4_)_4_ ^50^	
				Time (min)	Yield^a^	Time (min)	Yield^b^	Time (min)	Yield^c^	Time (min)	Yield^d^	Time (min)	Yield^e^	Time (min)	Yield^f^	Time (min)	Yield^g^
1	Me	H	Me	60	80%	60	85%	72	90%	5	95%	12	88%	15	92%	30	83%
2	Et	H	Me	120	70%	60	80%	60	80%	1	94%	10	90%				
3	Ph	H	Me	45	85%	35	80%	138	85%	25	93%	35	88%				
4	Me	H	Ph	90	75%	130	80%	90	85%	5	91%	17	87%	15	83%	30	75%
5	Et	H	Ph	120	85%	110	80%	108	80%	5	96%	15	90%	15	83%	30	80%
6	Ph	H	Ph	60	87%	40	82%	150	90%	40	95%	45	85%				
7	Me	H	4-MeC_6_H_4_	120	78%	240	60%	90	78%	5	86%	12	90%	15	85%	30	82%
8	Et	H	4-MeC_6_H_4_	240	70%	90	75%	90	75%	5	92%	10	89%				
9	Ph	H	4-MeC_6_H_4_	90	82%	120	80%	420	75%	45	85%	55	83%				
10	Me	^*n*^Pr	4-MeC_6_H_4_											15	85%	20	84%
11	Et	^*n*^Bu	4-MeC_6_H_4_											15	80%	20	75%
12	Me	Ph	4-MeC_6_H_4_											30	70%	60	70%

a Sulfonamide: 2 mmol; acid chloride: 2 mmol; catalyst: 0.35 g; CH_2_Cl_2_: 5 mL; reflux.

b Sulfonamide: 1 mmol; acid chloride: 2 mmol (in two portions); H_3_PO_4_/SiO_2_: 0.3 g; *n*-hexane: 5 mL; reflux.

c Sulfonamide: 1 mmol; acid chloride: 2 mmol; SiO_2_–Cl: 0.3 g; *n*-hexane: 5 mmol; reflux.

d Sulfonamide: 1 mmol; acid chloride: 2 mmol; Bi(OTf)_3_: 5 mol%; CHCl_3_: 5 mL; reflux.

e Sulfonamide: 1 mmol; acid chloride: 2 mmol; BiCl_3_: 10 mol%; CHCl_3_: 5 mL; reflux.

f Sulfonamide: 1 mmol; acid chloride: 1.1–1.5 mmol; Al(HSO_4_)_3_: 5–10 mol%; CH_2_Cl_2_: 5 mL; r.t.

g Sulfonamide: 1 mmol; acid chloride: 1.1–1.5 mmol; Zr(HSO_4_)_4_: 5–10 mol%; CH_2_Cl_2_ or CHCl_3_: 5 mL; r.t.

**Scheme 4:**
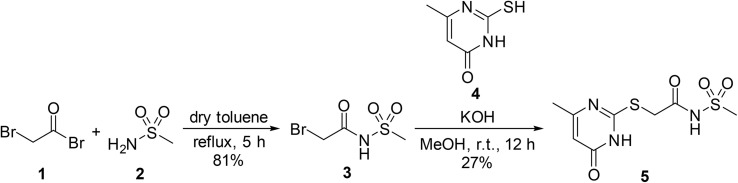
Synthesis of a pyrimidone-containing acyl sulfonamide.

A series of pyrimidone-containing acyl sulfonamides was evaluated by Bochet *et al.* for their anti-cancer properties.^53^ Pyrimidone-derived adduct 5 was prepared over two steps: 2-bromoacetyl bromide 1 was first combined with methanesulfonamide 2 under reflux to afford 2-bromo-*N*-(methylsulfonyl)acetamide 3 in 81% yield (Scheme 4). Intermediate 3 was then treated with 2-mercapto-6-methylpyrimidin-4(3*H*)-one 4 to produce target acyl sulfonamide 5. The rationale for employing an acid bromide is not provided, but is presumably due to the higher reactivity afforded by the bromide leaving group.

The use of *N*-acyl benzotriazoles has been pioneered by Katritzky.^54–56^*N*-Acyl benzotriazoles represent a viable alternative to acid chlorides, which can be difficult to prepare and handle.^57^ Reaction of *N*-acyl benzotriazoles with primary sulfonamides in the presence of sodium hydride in THF proceeded in good to excellent yields (Table 16).^58^ Aryl- (entries 1–5) and heteroaryl-containing (entries 6–11) substrates were readily transformed. However, 4-diethylaminophenyl and 4-pyridyl derivatives (entries 5–7) proved difficult to isolate, likely due to the increased aqueous solubility. Coupling of Cbz-protected amino acid-based benzotriazoles was also possible under these conditions (entries 12–14). Acylation of an acetazolamide-containing sulfonamide with both aromatic *N*-acyl derivatives (entries 15–17) and amino acid benzotriazole (entry 18) was similarly successful.

**Table 16:** Synthesis of *N*-acyl sulfonamides using *N*-acyl benzotriazoles and sulfonamides

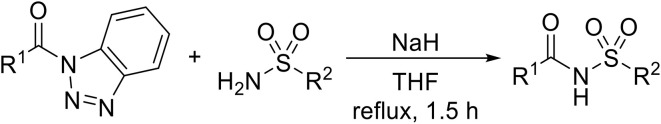			
Entry	R^1^	R^2^	Yield
1	4-MeC_6_H_4_	Me	85%
2	4-MeC_6_H_4_	4-MeC_6_H_4_	95%
3	4-MeOC_6_H_4_	Me	85%
4	4-MeOC_6_H_4_	4-MeC_6_H_4_	98%
5	4-(Et_2_N)C_6_H_4_	4-MeC_6_H_4_	98%
6	4-Pyridyl	4-MeC_6_H_4_	97%
7	4-Pyridyl	Me	98%
8	2-Furyl	4-MeC_6_H_4_	95%
9	4-Pyrrolyl	Me	80%
10	4-Pyrrolyl	4-MeC_6_H_4_	76%
11	2-Indolyl	4-MeC_6_H_4_	83%
12	Cbz–Ala	Me	78%
13	Cbz–Ala	4-MeC_6_H_4_	87%
14	Cbz–Phe	4-MeC_6_H_4_	90%
15	4-MeC_6_H_4_	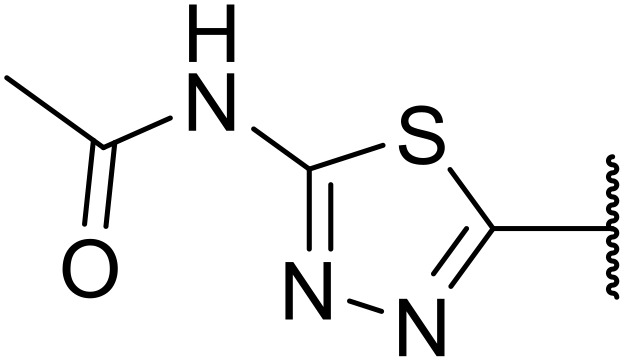	88%
16	4-Pyridyl	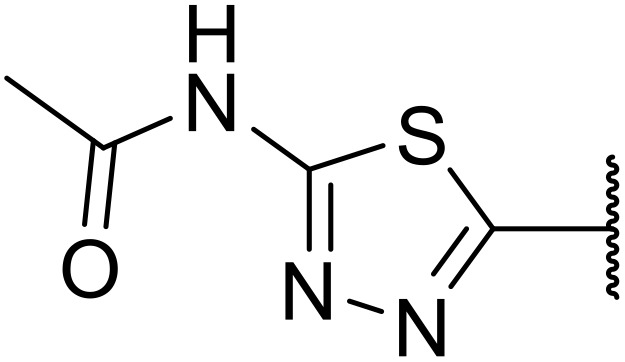	100%
17	4-O_2_NC_6_H_4_	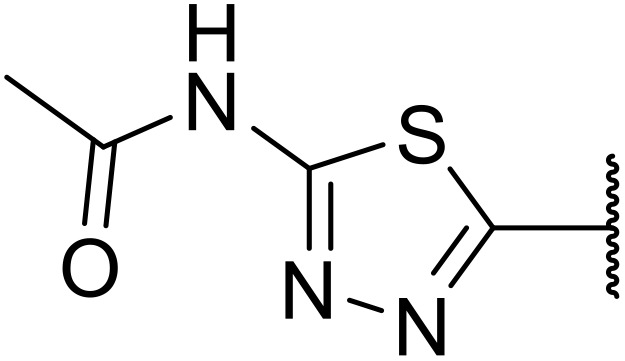	81%
18	Cbz–Ala	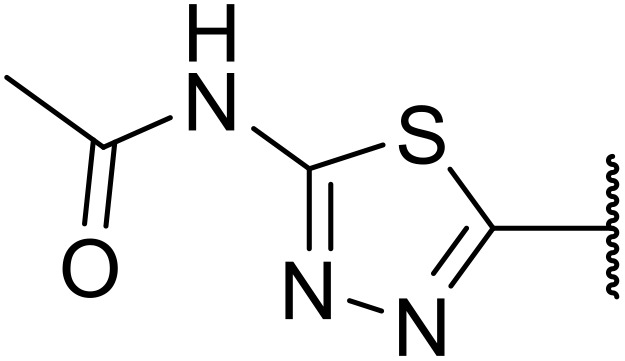	96%

The same group later expanded this methodology to encompass *N*-(α,β-unsaturated acyl)-benzotriazoles (Table 17).^59^ Two methods for coupling the benzotriazoles were established, employing either potassium *tert*-butoxide at 0 °C to room temperature (method A, entries 1–4) or sodium hydride at room temperature/reflux (method B, entries 5–12). β-Heteroarylacroyl benzotriazoles treated with substituted aromatic sulfonamides and potassium *tert*-butoxide generated the desired product in excellent yields (entries 2, 3 and 5), with the exception of a 2-thienyl acyl benzotriazole substrate (entry 4). Incorporation of methyl or phenyl groups at the α-position was not a barrier to successful coupling (entries 9 and 10).

**Table 17:** Synthesis of *N*-acyl sulfonamides from *N*-(α,β-unsaturated acyl)-benzotriazoles

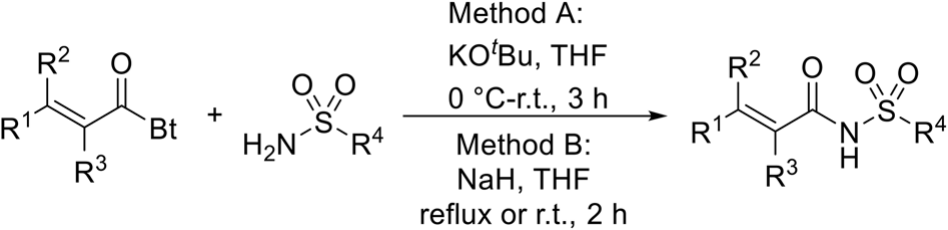						
Entry	R^1^	R^2^	R^3^	R^4^	Method	Yield
1	Ph	H	H	4-MeC_6_H_4_	A	80%
2	2-Furyl	H	H	4-MeC_6_H_4_	A	89%
3	2-Thienyl	H	H	4-MeC_6_H_4_	A	91%
4	2-Thienyl	H	H	Ph	A	48%
5	2-Thienyl	H	H	2,4,6-Me_3_C_6_H_2_	B	87%
6	Ph	H	H	2,4,6-Me_3_C_6_H_2_	B	65%
7	3,4,5-(MeO)_3_C_6_H_2_	H	H	3-F_3_CC_6_H_4_	B	84%
8	3,4,5-(MeO)_3_C_6_H_2_	H	H	4-^*t*^BuC_6_H_4_	B	56%
9	Ph	H	Me	4-^*t*^BuC_6_H_4_	B	84%
10	Ph	H	Ph	Me	B	60%
11	Ph	H	H	Me	B	63%
12	Me	Me	H	4-^*t*^BuC_6_H_4_	B	70%

## 4. Carboxylic acids and esters

Solid-phase organic synthesis is a widely used method for the generation of peptides and small molecule libraries.^60^ A polymer-supported version of EDC (1-(3-dimethylaminopropyl)-3-ethylcarbodiimide) was employed by Sturino to prepare various *N*-acyl sulfonamides (Table 18).^61^ This methodology was compatible with methanesulfonamide (entry 1) and a wide range of aromatic sulfonamides (entries 2–24). Coupling of heteroaryl sulfonamides was also possible under these conditions (entry 25).

**Table 18:** Application of polymer-supported EDC in the synthesis of *N*-acyl sulfonamides

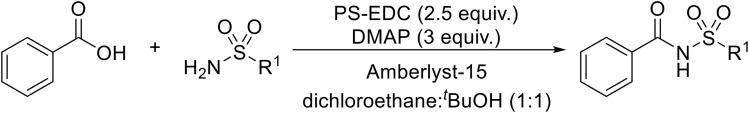		
Entry	R^1^	Yield
1	Me	66%
2	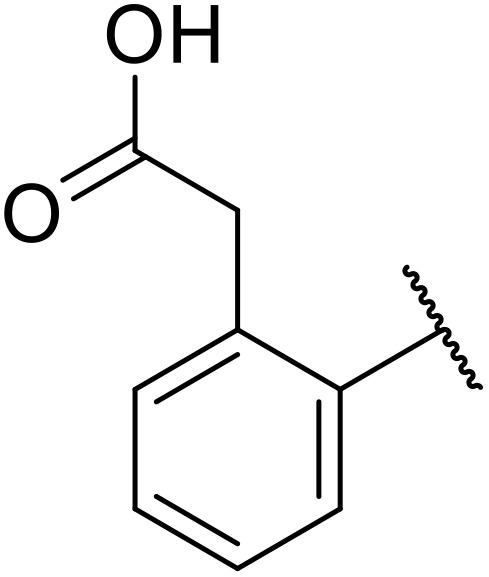	75%
3	Ph	56%
4	2-MeC_6_H_4_	75%
5	3-MeC_6_H_4_	73%
6	2,5-Me_2_C_6_H_3_	76%
7	4-MeOC_6_H_4_	68%
8	2,5-(MeO)_2_C_6_H_3_	78%
9	2-FC_6_H_4_	79%
10	2-ClC_6_H_4_	81%
11	3-ClC_6_H_4_	63%
12	4-ClC_6_H_4_	62%
13	3-BrC_6_H_4_	68%
14	2,4-Cl_2_C_6_H_3_	67%
15	2,5-Cl_2_C_6_H_3_	73%
16	2-Cl-6-MeC_6_H_3_	76%
17	3,4,5-Cl_3_C_6_H_2_	74%
18	C_6_F_5_	73%
19	2-O_2_NC_6_H_4_	63%
20	4-O_2_NC_6_H_4_	63%
21	2-F_3_CC_6_H_4_	63%
22	4-F_3_CC_6_H_4_	76%
23	Bn	64%
24	2-PhC_6_H_4_	73%
25	5-Br-2-Thienyl	57%

In 2013, Bach and co-workers discovered a novel series of ethyl 6-aminonicotinate acyl sulfonamides as P2Y_12_ receptor antagonists.^62^ Ethyl 6-azetidinylnicotinate acyl sulfonamides were synthesized *via* the initial reaction of ethyl 6-chloronicotinates with azetidine-3-carboxylic acids to afford azetidinylpyridine derivatives, which were subsequently coupled with various sulfonamides to yield the desired acyl sulfonamides (Table 19). Similarly, ethyl 6-piperidinylnicotinate acyl sulfonamides were prepared from the reaction of ethyl 6-chloronicotinates with piperidine-4-carboxylic acids, followed by sulfonamide coupling. Acyl sulfonamides with thienyl moiety were prepared in low to moderate yields (entries 1 and 2). Synthesis of acyl sulfonamides containing 2-phenylethyl and cyclohexyl was also successful. Treatment of carboxylic acids with benzene sulfonamides afforded the desired products in good yields (entry 5). Reaction with various benzyl sulfonamides proceeded in low to high yields (entries 6–16).

**Table 19:** Synthesis of ethyl-6-azetidinyl/piperidinyl nicotinate acyl sulfonamides

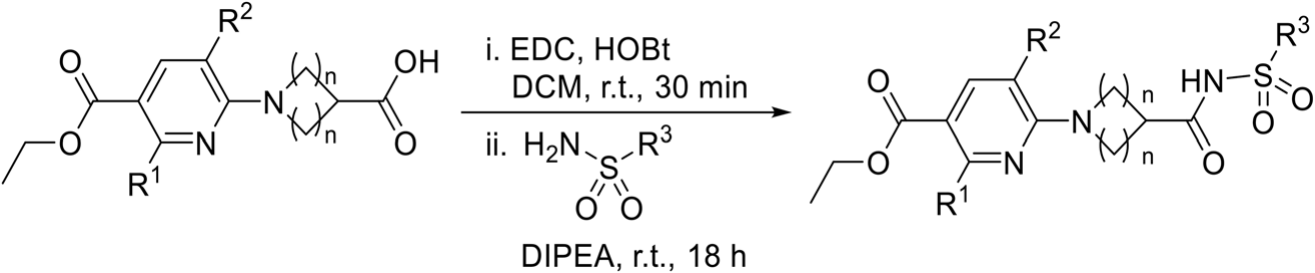					
Entry	R^1^	R^2^	R^3^	Yield	
				*n* = 1	*n* = 2
1	H	Cl	5-Cl-2-Thienyl	56%	24%
2	Me	CN	5-Cl-2-Thienyl	15%	45%
3	Me	CN	PhCH_2_CH_2_	39%	
4	Me	CN	CyCH_2_	11%	
5	Me	CN	Ph	70%	49%
6	Me	CN	Bn	54%	83%
7	Me	CN	2-MeBn	2%	
8	Me	CN	3-F_3_CBn	39%	
9	Me	CN	3-MeBn	15%	
10	Me	CN	4-ClBn	48%	
11	Me	CN	4-FBn	43%	80%
12	Me	CN	4-MeBn	15%	45%
13	Me	CN	4-CH_2_OHBn	19%	
14	Me	CN	4-OMeBn		45%
15	Me	CN	4-^*i*^PrBn		56%
16	Me	CN	2,4-F_2_Bn		34%

**Scheme 5:**
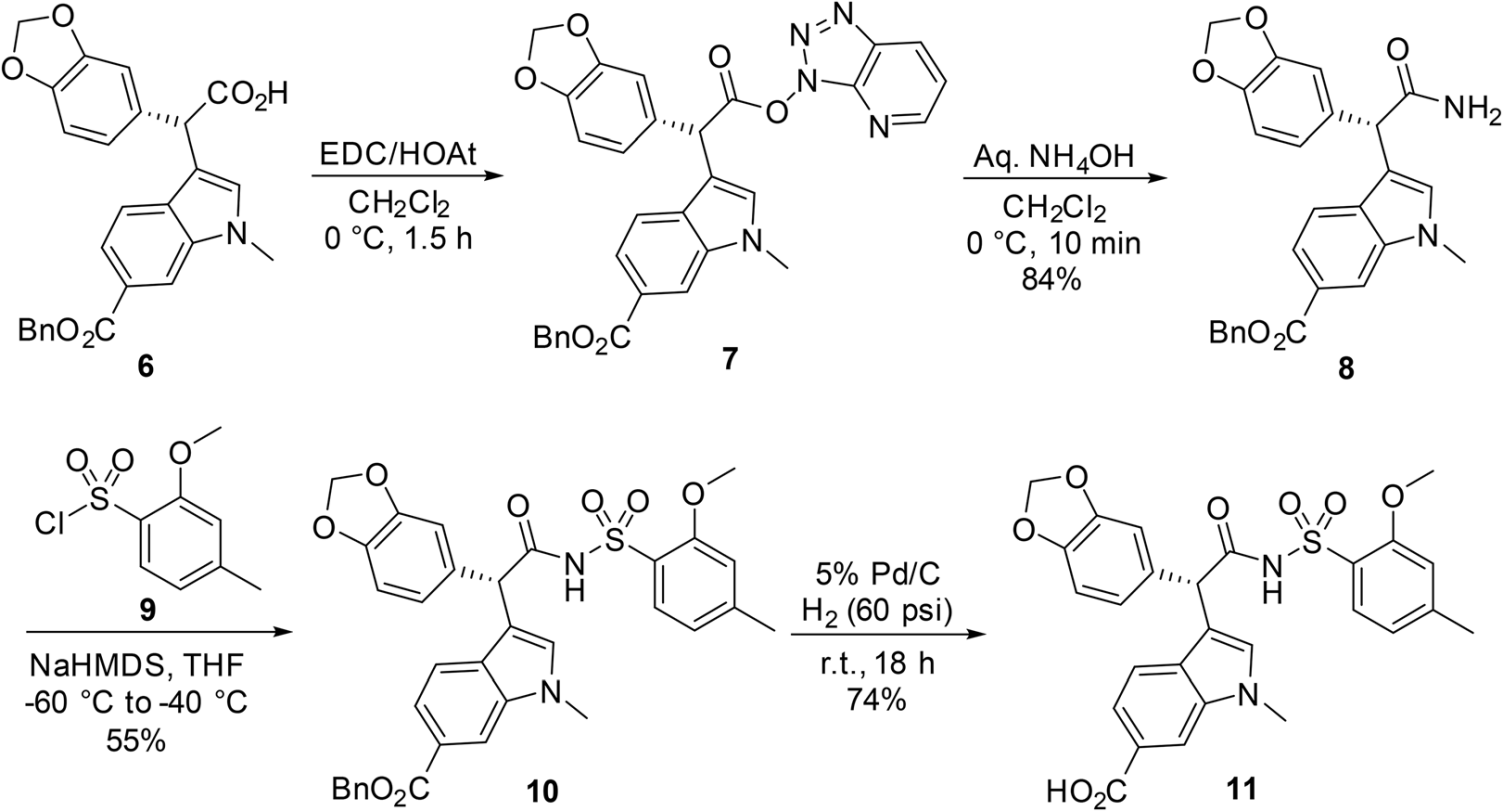
Preparation of enantiopure acyl sulfonamides.

Ellis described a racemisation-free pathway for the synthesis of the endothelin-A antagonist acyl sulfonamide 11.^63^ Initial attempts at coupling enantiopure 6 using EDC/HOAt and DMAP (stoichiometric or catalytic) resulted in complete racemisation. However, when enantiopure acid 6 was treated with EDC and HOAt, the corresponding configurationally stable HOAt ester 7 was successfully isolated (Scheme 5). Subsequent treatment with saturated aqueous ammonia afforded enantiomerically pure amide 8. Reaction of amide 8 with sulfonyl chloride 9 in the presence of NaHMDS in THF at low temperature, afforded protected acyl sulfonamide 10 which was subsequently hydrogenolysed to provide enantiomerically pure acyl sulfonamide 11.

Rad *et al.* described the use of cyanuric chloride (2,4,6-trichloro-1,3,5-triazine or TCT) for the one-pot synthesis of *N*-acyl sulfonamides from carboxylic acids.^64^ A combination of alumina and triethylamine in anhydrous acetonitrile afforded high yields and short reaction times (Table 20). Coupling of aliphatic and aromatic carboxylic acids with sulfonamides was successful under these conditions (entries 1–16). Conversion of carboxylic acids containing electron-withdrawing substituents (entries 5 and 16) necessitated longer reaction times. Moderate yields were noted when a benzamide moiety was incorporated into the acid substrate (entries 10–12).

**Table 20:** Cyanuric acid-mediated coupling of sulfonamides and carboxylic acids

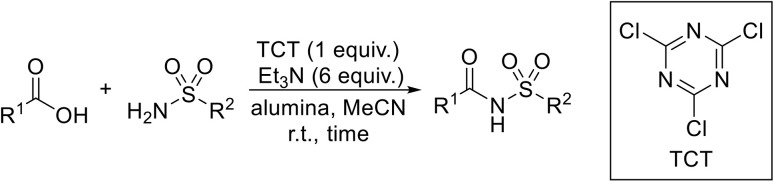				
Entry	R^1^	R^2^	Time (min)	Yield
1	Ph	Ph	60	92%
2	Ph	4-MeC_6_H_4_	40	85%
3	Ph	Me	60	65%
4	4-ClC_6_H_4_	Ph	50	85%
5	4-ClC_6_H_4_	Me	100	76%
6	4-MeOC_6_H_4_	Ph	50	86%
7	4-O_2_NC_6_H_4_	Ph	90	75%
8	4-MeOC_6_H_4_CH_2_	Ph	90	65%
9	(*E*)-PhCH=CH	Ph	120	62%
10	BzNHCH_2_	Ph	75	60%
11	BzNHCH_2_	4-MeC_6_H_4_	70	55%
12	BzNHCH_2_	Me	70	60%
13	Me	Ph	80	74%
14	Me	4-MeC_6_H_4_	75	70%
15	Me	Me	75	68%
16	3-Pyridyl	Ph	120	70%

**Scheme 6:**
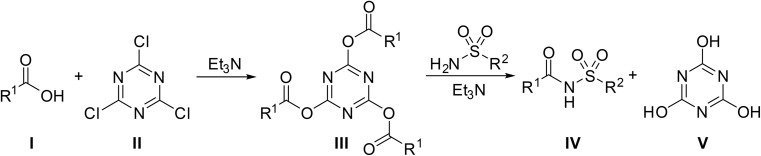
Proposed mechanism for cyanuric acid couplings.

The mechanism likely proceeds *via* an S_N_-Ar type reaction between the carboxylic acid I and cyanuric chloride II in the presence of triethylamine, resulting in the formation of an active carboxylate cyanuric ester intermediate III (Scheme 6). The chemoselectivity arises from the fact that sulfonamides remain unreactive toward cyanuric chloride under the reaction conditions, whereas carboxylic acids readily form carboxylate anions, which are significantly more nucleophilic than sulfonamides. Consequently, cyanuric chloride reacts exclusively with the carboxylate species to produce the active ester. The ester then combines with a sulfonamide to form *N*-acyl sulfonamide product IV, with the concomitant liberation of cyanuric acid V.

A one-pot methodology for coupling carboxylic acids and sulfonamides using the Mukaiyama reagent (2-chloro-1-methylpyridinium iodide or CMPI) was developed by Chen and Luo.^65^ A 2 : 1 ratio of sulfonamide to carboxylic acid, with triethylamine as base and catalytic DMAP in dichloromethane, proved optimum (Table 21). Coupling of methanesulfonamide with aromatic (entries 1–7), heteroaromatic (8–10), and aliphatic (entries 11–13) acids was successful. The sulfonamide partner could also be varied, ranging from aliphatic (entries 1–13, 15 and 16) to aromatic (entries 14 and 17–19).

**Table 21:** Coupling of carboxylic acids and sulfonamides using the Mukaiyama reagent

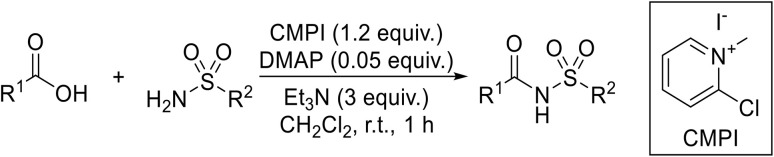			
Entry	R^1^	R^2^	Yield
1	4-ClC_6_H_4_	Me	81%
2	3-ClC_6_H_4_	Me	82%
3	2-ClC_6_H_4_	Me	73%
4	3-MeOC_6_H_4_	Me	89%
5	2-MeC_6_H_4_	Me	91%
6	2-F_3_CC_6_H_4_	Me	78%
7	4-O_2_NC_6_H_4_	Me	78%
8	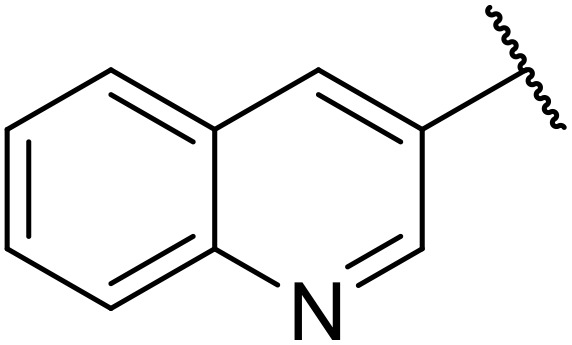	Me	72%
9	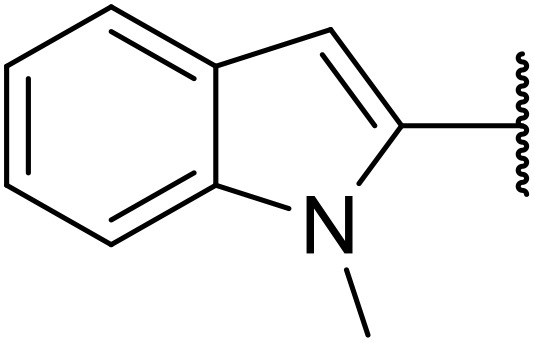	Me	78%
10	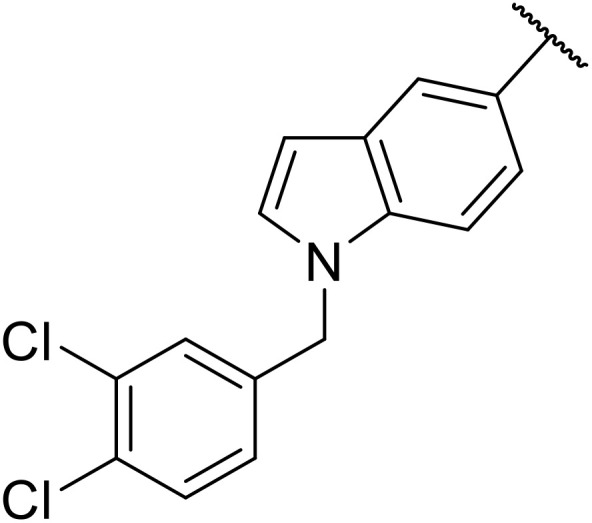	Me	58%
11	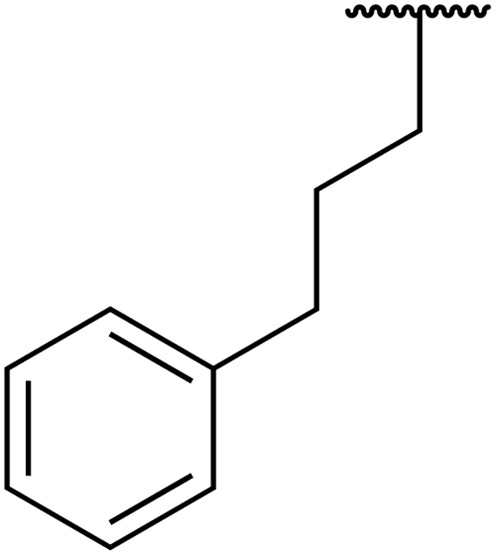	Me	75%
12	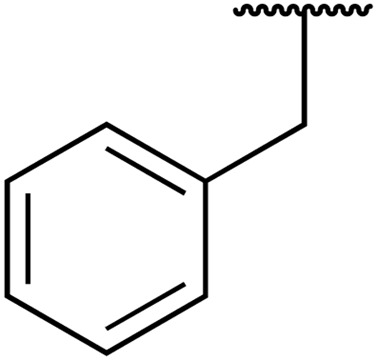	Me	71%
13	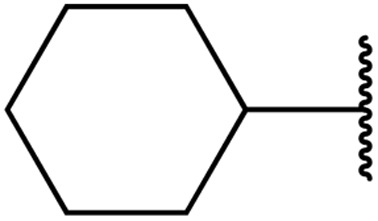	Me	77%
14	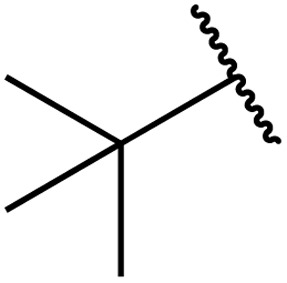	Ph	36%
15	Ph	^*i*^Pr	83%
16	Ph	Cyclopropyl	89%
17	Ph	Ph	83%
18	Ph	3-ClC_6_H_4_	84%
19	Ph	2-ClC_6_H_4_	87%

Manabe *et al.* reported the successful exploitation of sulfonyl isocyanates to access *N*-acyl sulfonamides from carboxylic acid (Table 22).^66^ Carboxylic acids containing functional groups such as esters (entry 1), alkenes (entry 2), halides (entry 3), acetals (entries 7 and 8), and ethers (entries 5 and 9) were transformed in excellent yields. Notably, reaction of *N*-Cbz-l-glutamic acid α-benzyl afforded the *N*-acyl sulfonamide in quantitative yield (entry 6) without the formation of pyroglutamate side products previously reported.^67^ This strategy was adapted to the preparation of biologically active nucleoside derivatives (entry 8), as well as anti-tumor agents (entry 10), both in high yields.

**Table 22:** Preparation of *N*-acyl sulfonamides using sulfonyl isocyanates

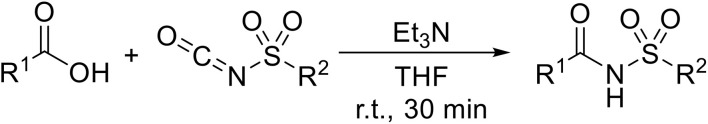			
Entry	R^1^	R^2^	Yield
1	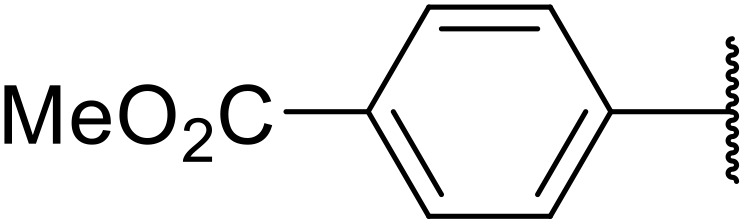	4-MeC_6_H_4_	97%
2	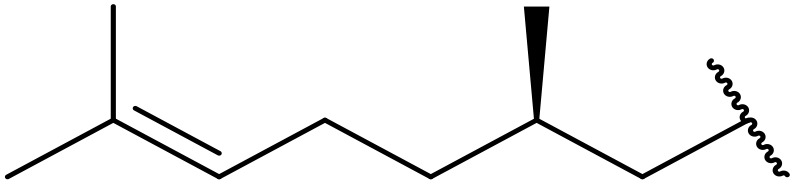	4-MeC_6_H_4_	85%
3	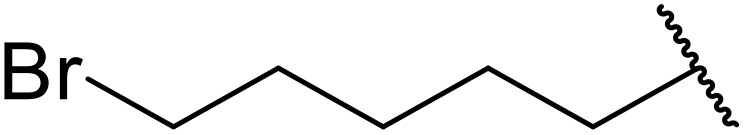	4-MeC_6_H_4_	96%
4	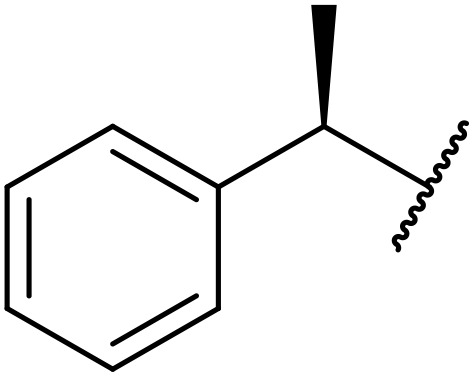	4-MeC_6_H_4_	100%
5	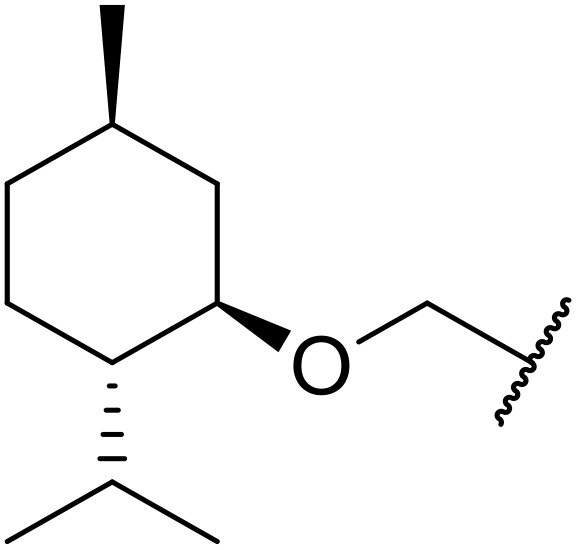	4-MeC_6_H_4_	83%
6	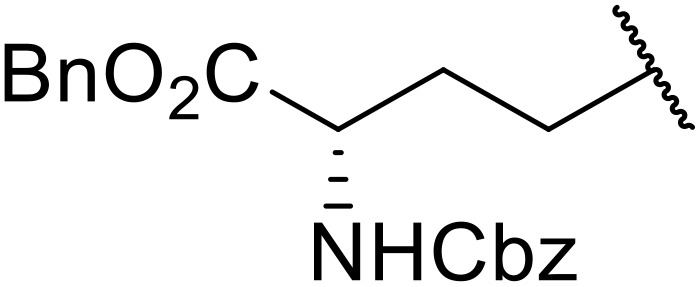	4-MeC_6_H_4_	100%
7	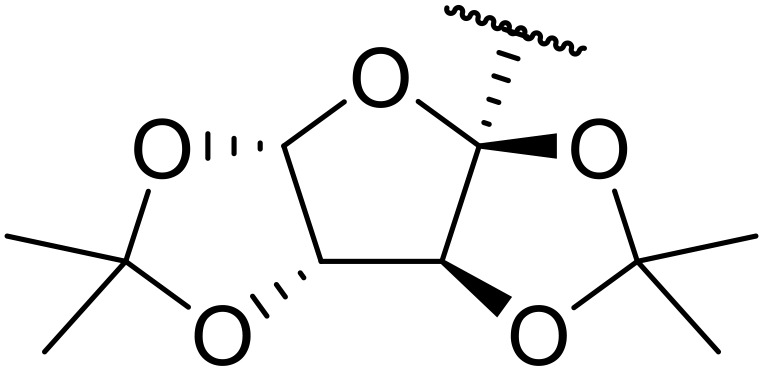	4-MeC_6_H_4_	81%
8	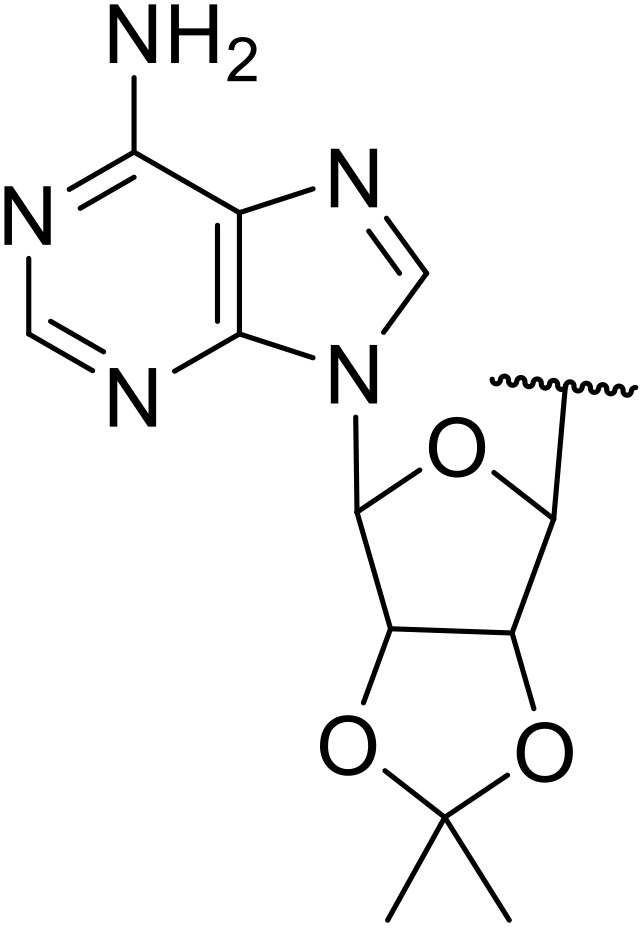	4-MeC_6_H_4_	89%
9	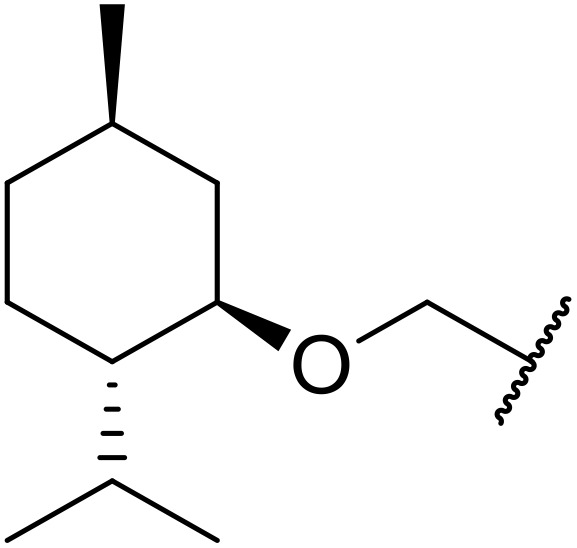	Me	90%
10	2,4-Cl_2_C_6_H_3_	4-ClC_6_H_4_	93%^a^

a Reaction was performed at 50 °C in 5 min.

Ashfeld and colleagues described a chlorophosphite-mediated Staudinger-like acylation of sulfonyl azides as a route to *N*-acyl sulfonamides.^68^ This approach, which relies on a Staudinger-type ligation of the C–N bond, was initially optimised for the preparation of amides and later adapted to *N*-acyl sulfonamides (Table 23). The best outcomes were obtained with ClP(pin), or 2-chloro-4,4,5,5-tetramethyl-1,3,2-dioxaphospholane, and triethylamine with a 1.3 : 1 ratio of acid to azide. Aromatic carboxylic acids (entries 1–6), ranging from electron-rich to electron-poor, in addition to heteroaromatic substrates (entry 7), reacted with tosyl azide in good to excellent yields. This transformation was highly chemoselective with *para*-substituted alcohol (entry 8), aldehyde (entry 9) and ketone (entry 10) functional groups well tolerated. Conversion of aliphatic substrates was also successful (entries 15–17), albeit in low yields for sterically encumbered pivalic acid (entry 17). This utility of this methodology was exemplified by the synthesis of anti-tumor agent LY573636 in excellent 97% yield (entry 18).^69^

**Table 23:** Direct Staudinger-like acylation of carboxylic acids with sulfonyl azides

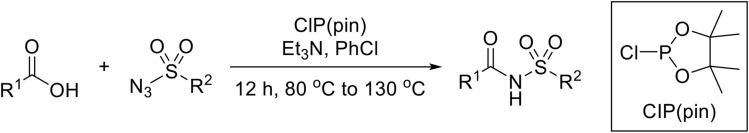			
Entry	R^1^	R^2^	Yield
1	Ph	4-MeC_6_H_4_	93%
2	4-MeOC_6_H_4_	4-MeC_6_H_4_	96%
3	4-F_3_CC_6_H_4_	4-MeC_6_H_4_	81%
4	4-NCC_6_H_4_	4-MeC_6_H_4_	96%
5	4-ClC_6_H_4_	4-MeC_6_H_4_	91%
6	2-IC_6_H_4_	4-MeC_6_H_4_	83%
7	2-Pyrrolyl	4-MeC_6_H_4_	81%
8	4-(HOCH_2_)C_6_H_4_	4-MeC_6_H_4_	64%
9	4-(OHC)C_6_H_4_	4-MeC_6_H_4_	60%
10	4-(MeCO)C_6_H_4_	4-MeC_6_H_4_	82%
11	2-(PhCO)C_6_H_4_	Bn	34%
12	(*E*)-PhCH=CH	4-MeC_6_H_4_	93%
13	(*E*)-PhCH=CH	Me	76%
14	Bn	4-MeC_6_H_4_	94%
15	^*n*^Hept	4-MeC_6_H_4_	91%
16	Cy	4-MeC_6_H_4_	80%
17	^*t*^Bu	4-MeC_6_H_4_	56%
18	2,4-Cl_2_C_6_H_3_	5-Br-2-Thienyl	97%

**Scheme 7:**
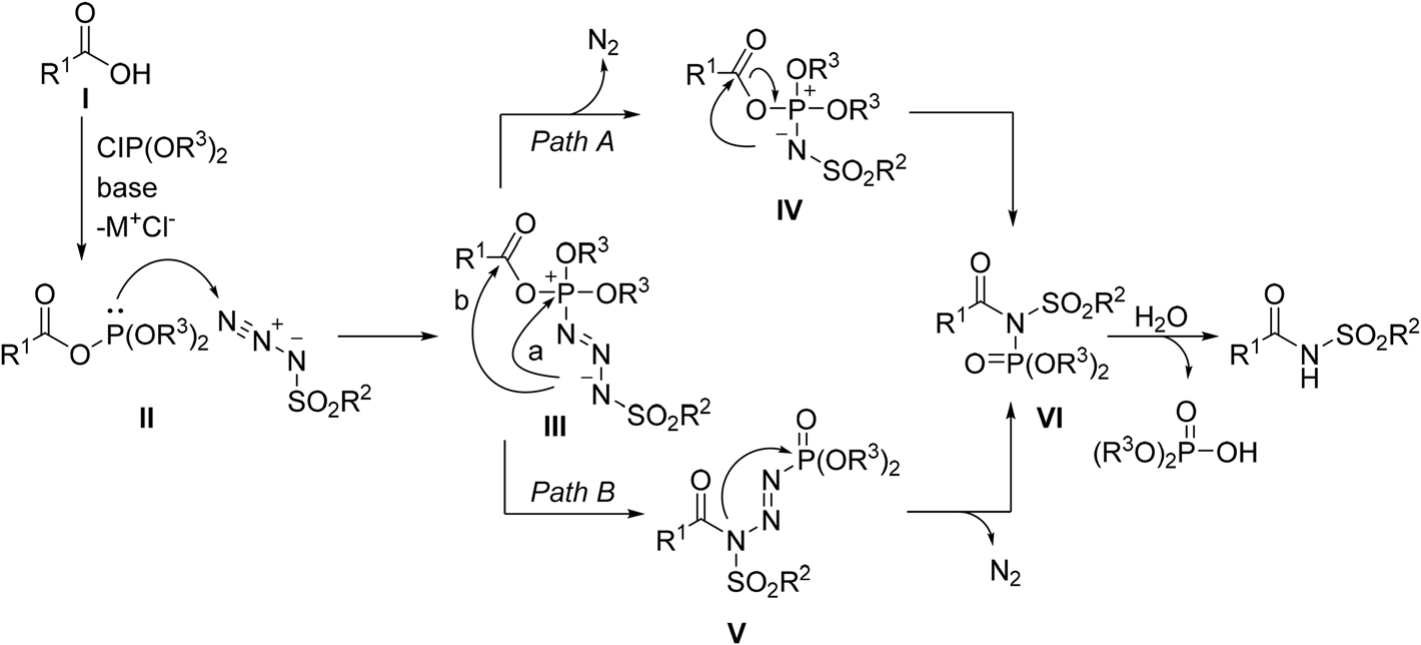
Proposed reaction pathways.

Following initial formation of phosphite ester II, subsequent reaction with the azide generates a common phosphazide intermediate III (Scheme 7). The mechanism can then plausibly proceed by two different pathways. Path A involves acyl substitution and elimination of nitrogen gas to generate an ester azaylide IV, which undergoes [1,3]-acyl migration to the phosphorimide VI. Subsequent hydrolysis liberates the acyl sulfonamide target. Path B proceeds by formation of phosphoryl triazine V which then rearranges to produce the same phosphorimide VI as path A.

A novel, cobalt-catalysed process for the preparation of *N*-acyl sulfonamides from carboxylic acids has been developed by Ji and colleagues.^70^ During a study of insertion reactions of isocyanides, the authors serendipitously observed how a cobalt catalyst promoted the formation of *N*-tosylbenzamide from benzoic acid and tosyl azide.^71^ 5 mol% dicobalt octacarbonyl in acetonitrile at 80 °C proved optimum (Table 24). This chemistry was compatible with aromatic (entries 1–14), *trans*-cinnamic acid (entry 15), aliphatic (entry 16) and heteroaromatic acids (entries 17 and 18). Other azides, ranging from electron-rich (entries 19 and 20) to electron-poor (21–23) and aliphatic examples (entries 25–27), were also well tolerated. Anti-tumor agent LY573636 could be prepared on a gram scale under these conditions (entry 28).

**Table 24:** Co-catalysed coupling of carboxylic acids and sulfonylazides

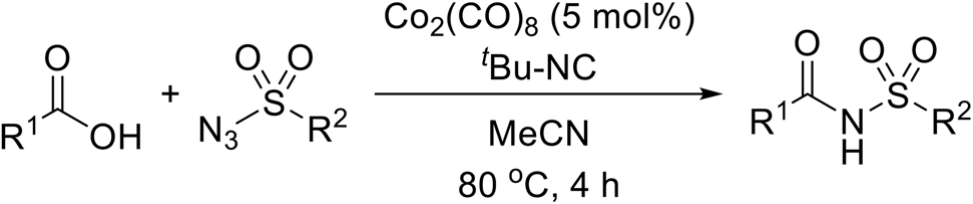			
Entry	R^1^	R^2^	Yield
1	Ph	4-MeC_6_H_4_	96%
2	2-MeC_6_H_4_	4-MeC_6_H_4_	97%
3	3-MeC_6_H_4_	4-MeC_6_H_4_	98%
4	4-MeC_6_H_4_	4-MeC_6_H_4_	96%
5	4-MeOC_6_H_4_	4-MeC_6_H_4_	71%
6	2-FC_6_H_4_	4-MeC_6_H_4_	85%
7	2-ClC_6_H_4_	4-MeC_6_H_4_	80%
8	2-BrC_6_H_4_	4-MeC_6_H_4_	79%
9	3-ClC_6_H_4_	4-MeC_6_H_4_	81%
10	3-BrC_6_H_4_	4-MeC_6_H_4_	83%
11	3-IC_6_H_4_	4-MeC_6_H_4_	85%
12	4-ClC_6_H_4_	4-MeC_6_H_4_	80%
13	4-BrC_6_H_4_	4-MeC_6_H_4_	76%
14	4-F_3_CC_6_H_4_	4-MeC_6_H_4_	70%
15	(*E*)-PhCH=CH	4-MeC_6_H_4_	86%
16	Cyclobutyl	4-MeC_6_H_4_	74%
17	3-Furyl	4-MeC_6_H_4_	91%
18	2-Thienyl	4-MeC_6_H_4_	93%
19	Ph	Ph	93%
20	Ph	4-MeOC_6_H_4_	89%
21	Ph	4-CNC_6_H_4_	71%
22	Ph	4-BrC_6_H_4_	80%
23	Ph	4-IC_6_H_4_	94%
24	Ph	2-Naphthyl	93%
25	Ph	Et	83%
26	Ph	^*n*^Pr	69%
27	Ph	^*n*^Bu	72%
28	2,4-Cl_2_C_6_H_3_	5-Br-2-Thienyl	60%

**Scheme 8:**
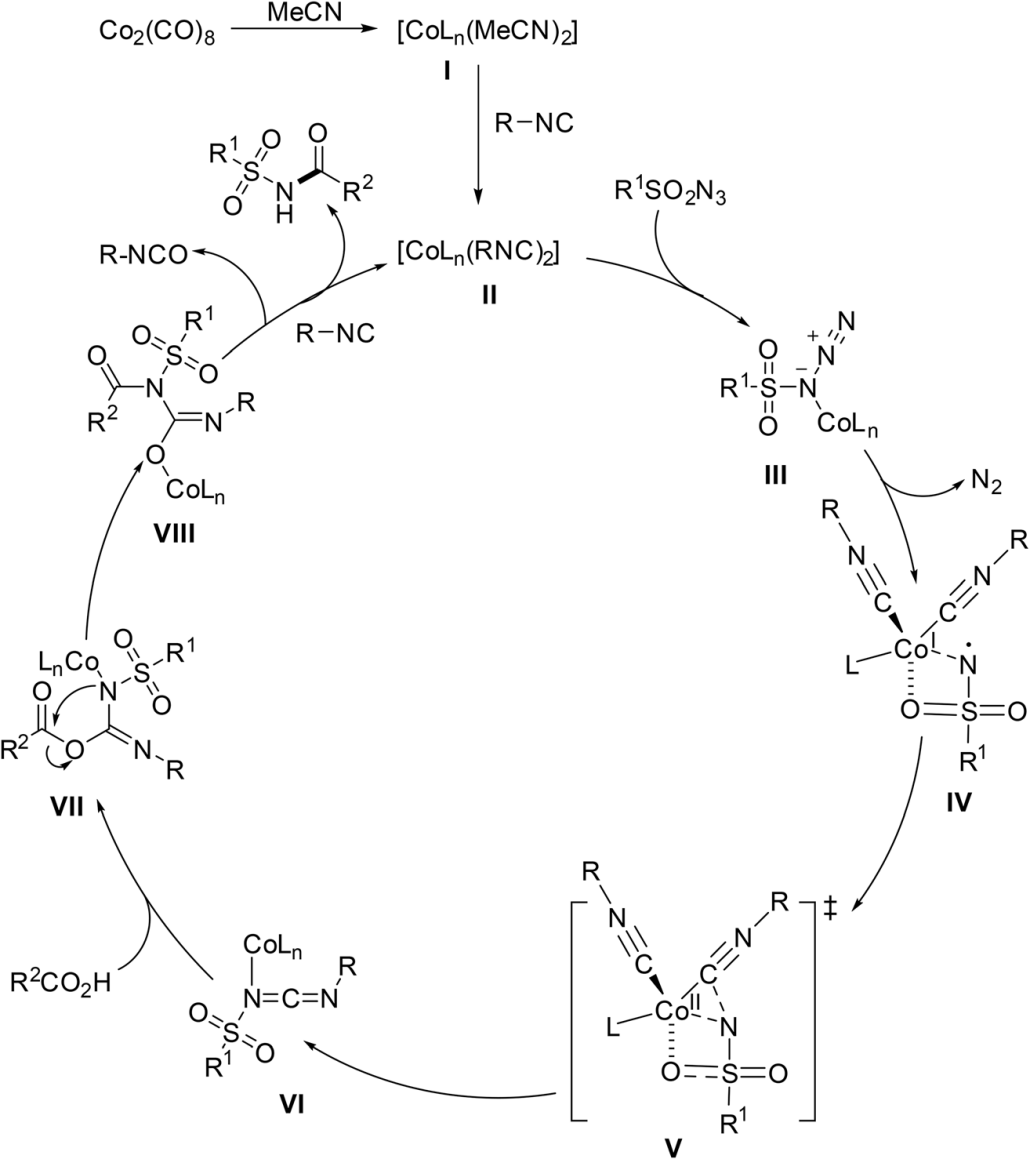
Proposed catalytic cycle.

The proposed catalytic cycle begins with generation of intermediate I*via* ligand exchange from Co_2_(CO)_8_ in acetonitrile, which then reacts with *tert*-butyl isocyanide to form active complex II (Scheme 8). Reaction of II with a sulfonyl azide affords complex III which dissociates to liberate nitrogen gas and intermediate IV. A subsequent coupling reaction with the coordinated isocyanide molecule furnishes VI which is attacked by the carboxylic acid forming VII. Rearrangement of VII leads to intermediate VIII which decomposes to generate the target acyl sulfonamide.

Williams *et al.* developed an effective methodology for transforming amino acids into acyl sulfonamides *via* a thio acid intermediate.^72^ This approach involves the initial conversion of a protected amino acid into a mixed anhydride and subsequent reaction with trimethylsilyl thiolate to form a thioester (Table 25). The thioester rearranges to a thionoester and forms a thio acid upon methanolysis. Addition of sulfonyl azide generates the acyl sulfonamide product. Coupling of either base- (entries 1–3) or acid-sensitive (entries 3–5) substrates was equally successful.

**Table 25:** Synthesis of acyl sulfonamides from amino acids and sulfonyl azides

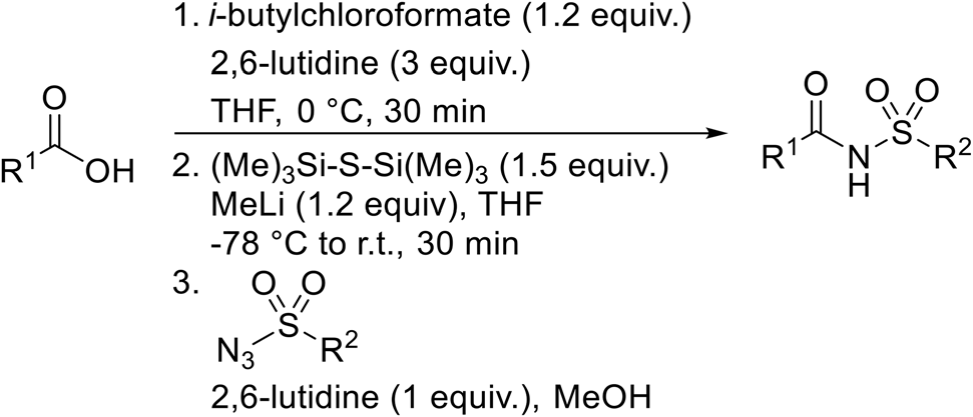			
Entry	R^1^	R^2^	Yield
1	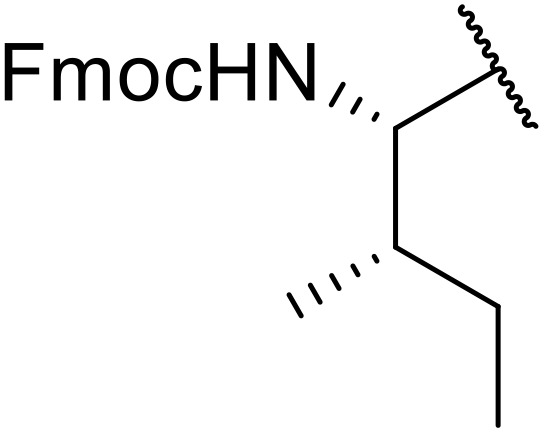	4-(HO_2_C)C_6_H_4_	86%
2	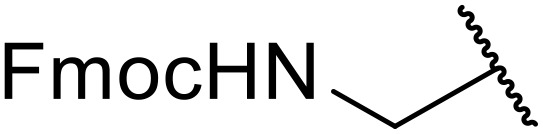	4-(HO_2_C)C_6_H_4_	83%
3	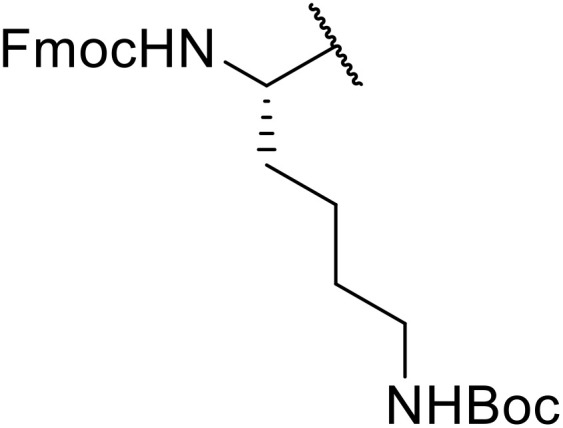	4-(HO_2_C)C_6_H_4_	98%
4	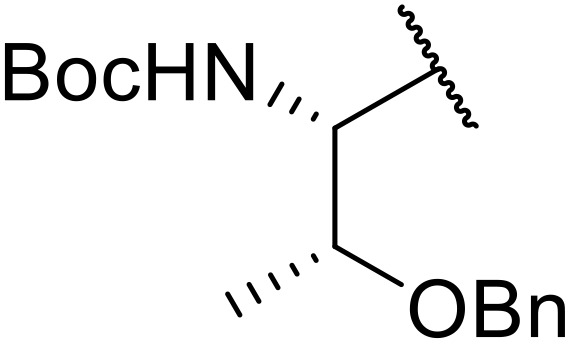	(TMS)CH_2_CH_2_	96%
5	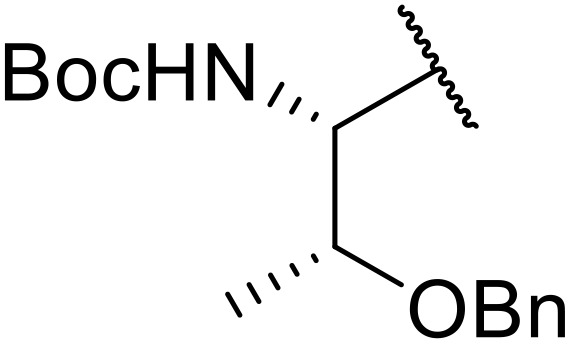	(TMS)CH_2_CH_2_	94%

In an effort to discover novel anti-cancer agents, Sławiński and co-workers pioneered a novel approach to acyl sulfonamides from carboxylic acids and *N*-[4-chloro-5-methyl-2-(R^1^-methylthio)benzenesulfonyl]cyanamide potassium salts (Table 26).^73^ Cyanamide potassium salts containing alkene (entry 1) or amide (entry 3) moieties produced high yields, in contrast to alkyne substrates (entry 2). Aryl/heteroaryl derivatives of cyanamide potassium salts afforded moderate to excellent yields (entries 4–15). Variation of the acid component from propanoic acid (entries 16–25), to isobutyric (entry 26) and 3-cyclohexenepropanoic acid (entry 27) proved similarly successful. Coupling of solid acid reagents, such as benzoic acid (entry 28) or *trans*-cinnamic acid (entries 29 and 30), were conducted in water or toluene, in contrast to solvent-free conditions for other acids.

**Table 26:** *N*-Acyl sulfonamides from carboxylic acids and cyanamide potassium salts

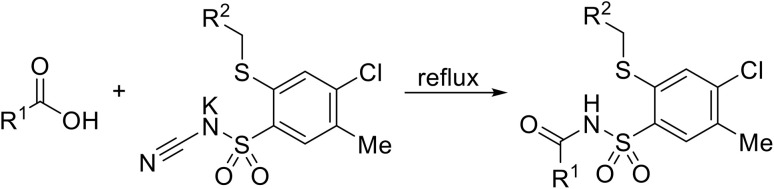					
Entry	R^1^	R^2^	Solvent	Time (h)	Yield
1	Me	H_2_C=CH		15	72%
2	Me	HC≡C		17	35%
3	Me	H_2_NCO		4.5	60%
4	Me	Ph		2	54%
5	Me	3-F_3_CC_6_H_4_		2	84%
6	Me	4-F_3_CC_6_H_4_		1.5	50%
7	Me	4-ClC_6_H_4_		2	50%
8	Me	4-MeOC_6_H_4_		1	51%
9	Me	4-Pyridyl		1.5	45%
10	Me	1-Naphthyl		1.5	54%
11	Me	2-Naphthyl		2	50%
12	Me	2-Quinolinyl		2	52%
13	Me	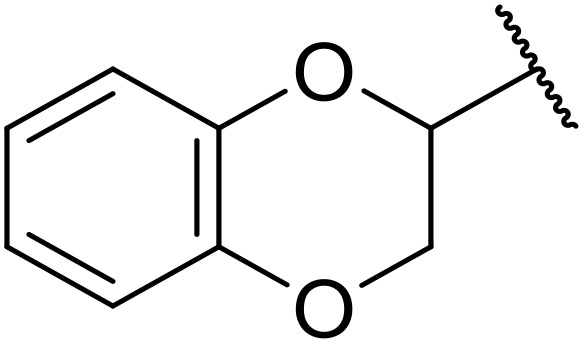		1	55%
14	Me	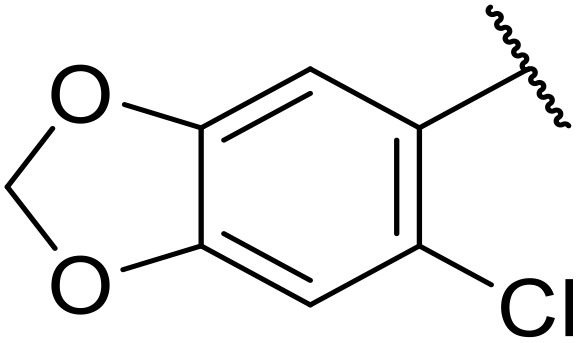		1.5	59%
15	Me	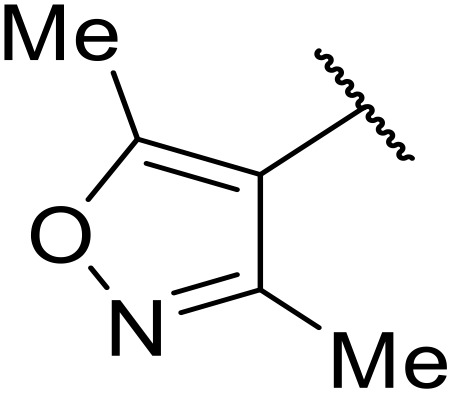		1.5	62%
16	Et	Ph		1	86%
17	Et	3-F_3_CC_6_H_4_		2	68%
18	Et	4-F_3_CC_6_H_4_		1	93%
19	Et	4-ClC_6_H_4_		2	68%
20	Et	4-MeOC_6_H_4_		1	85%
21	Et	4-Pyridyl		2	65%
22	Et	2-Naphthyl		1.5	86%
23	Et	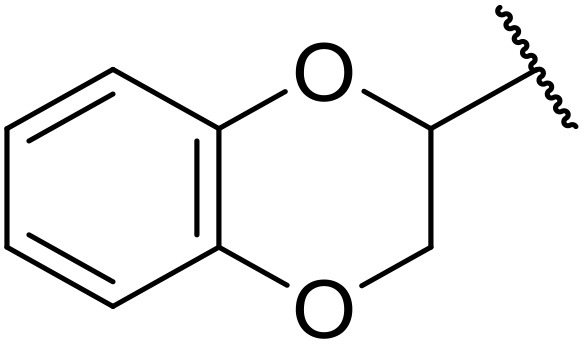		1.75	87%
24	Et	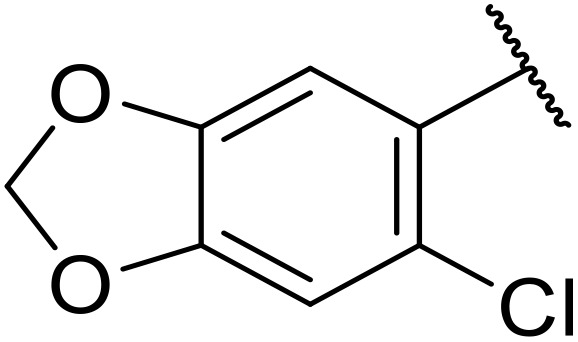		1	81%
25	Et	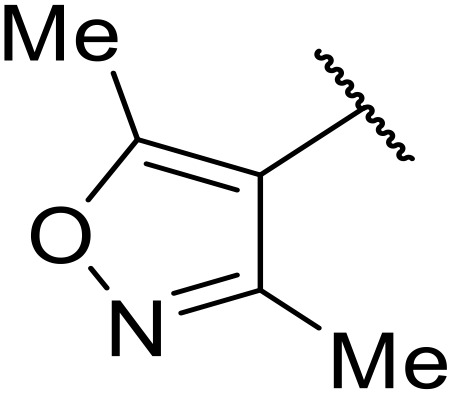		1.5	68%
26	(CH_3_)_2_CH	Ph		2	95%
27	Cy–CH_2_CH_2_	Ph		1.5	81%
28	Ph	Ph	Water	144	72%
29	(*E*)-PhCH=CH	Ph	Water	96	76%
30	(*E*)-PhCH=CH	1-Naphthyl	Toluene	120	50%

**Scheme 9:**
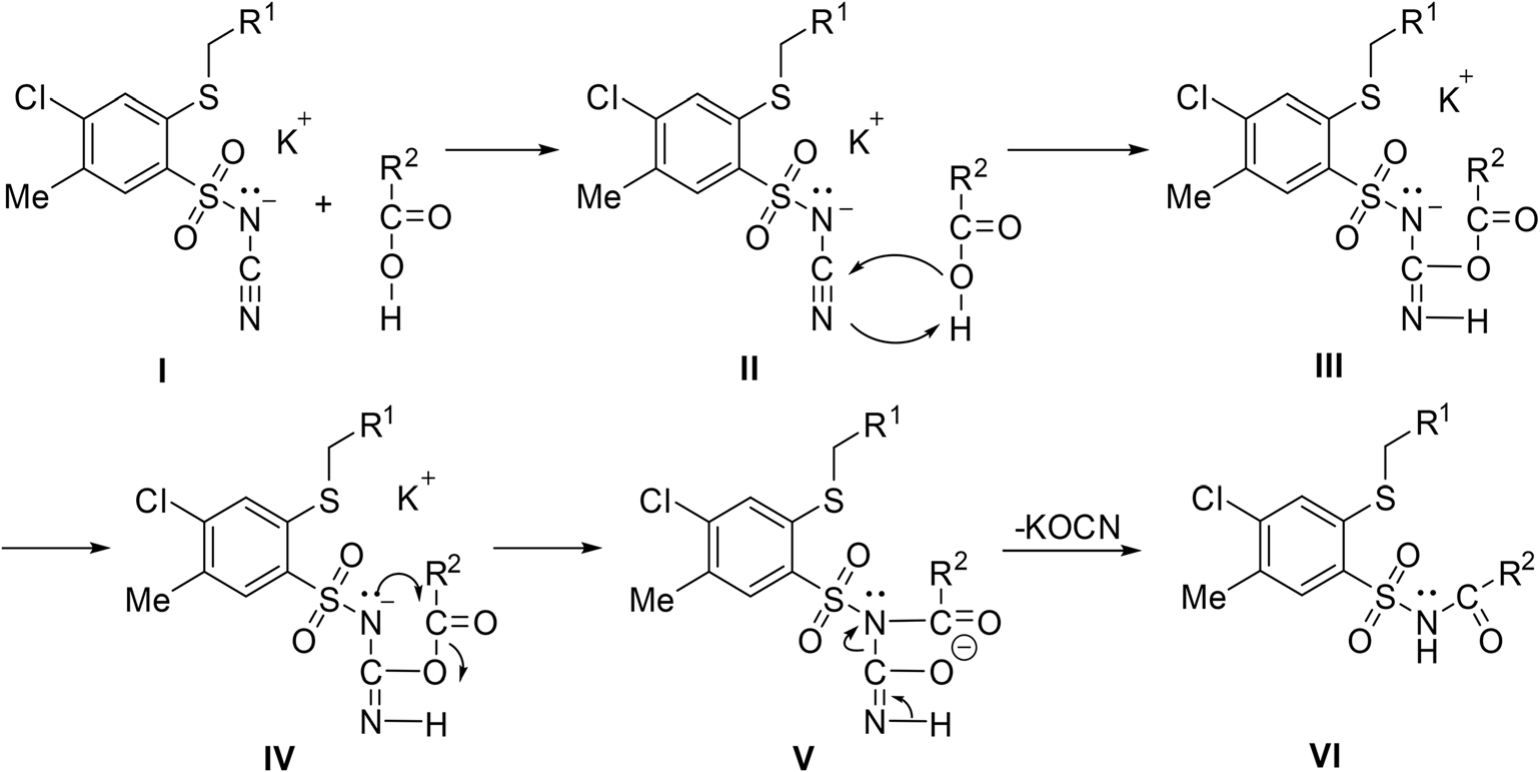
Proposed mechanism for the coupling of cyanamide potassium salts with carboxylic acids.

The reaction is initiated by protonation of the nitrile nitrogen by the carboxylic acid (Scheme 9). Simultaneously, the carboxylate anion accepts the lone pair from the nitrile, activating the nitrile for nucleophilic attack. The carboxylic acid serves dual roles as a proton donor and a stabilizing agent for the nitrile activation. This is followed by a subsequent attack of the sulfonamide nitrogen anion on the carbonyl, leading to C–O cleavage. Elimination of cyanate produces the required acyl sulfonamide.

**Scheme 10:**
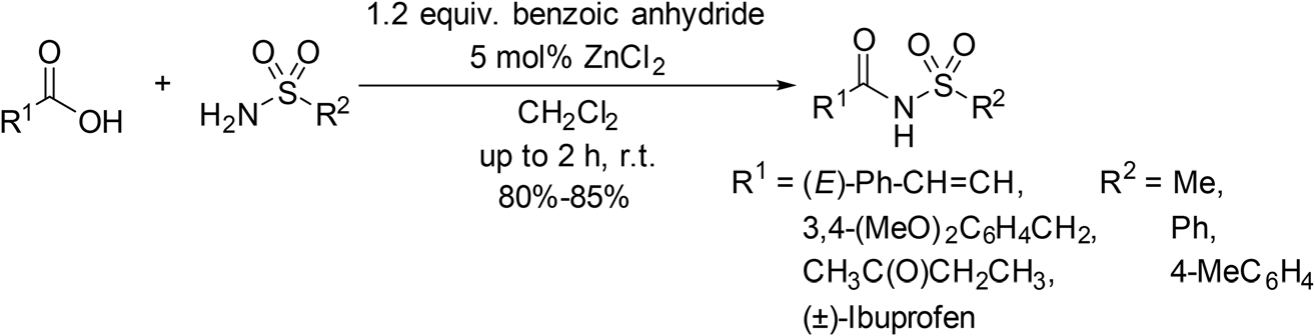
ZnCl_2_-catalysed *N*-acylation *via* mixed anhydrides.

Reddy and co-workers expanded their methodology for the *N*-acylation of sulfonamides from acid anhydrides (Table 3) to carboxylic acids *via* a mixed anhydride intermediate (Scheme 10).^38^ Treating sulfonamides with *trans*-cinnamic, 3,4-dimethoxyphenylacetic acid, levulinic acid and (±)-ibuprofen furnished the corresponding *N*-acyl sulfonamide products in up to 85% yield.

Sagar outlined a procedure for synthesising orthogonally protected *N*-acyl sulfonamide-tethered peptides from commercially available amino acids *via* a selenocarboxylate intermediate.^74^ Initially, the selected amino acid was converted to its mixed anhydride and subsequently selenated with NaBH_2_Se_3_ (Table 27). This methodology allowed for the preparation of sulfonamide-linked peptides (entries 1–7), *N*-acyl sulfonamide-derived dipeptide substrates (entries 8 and 9), as well as aryl sulfonamide-derived peptides (entries 10–17), all in excellent yields. Epimerisation studies involving optically pure Cbz–(l)-Phg–OH and Cbz–(d)-Phg–OH confirmed that racemisation was not observed over the course of the reaction.

**Table 27:** Synthesis of orthogonally protected *N*-acyl sulfonamide tethered dipeptides

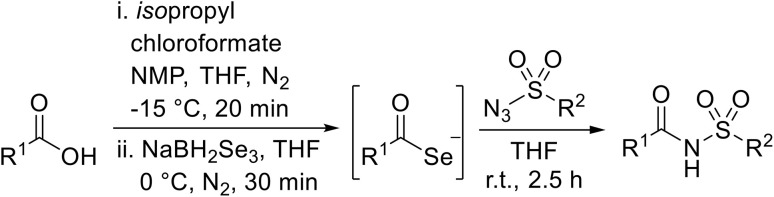			
Entry	R^1^	R^2^	Yield
1	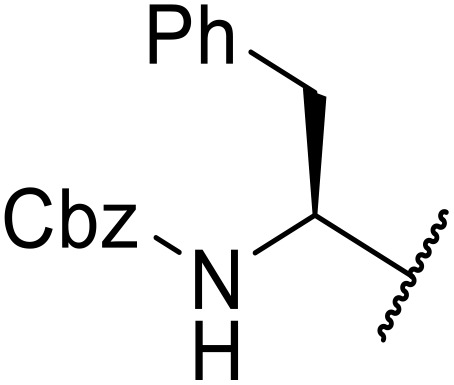	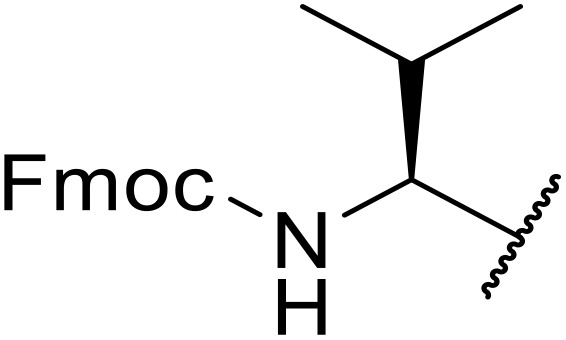	91%
2	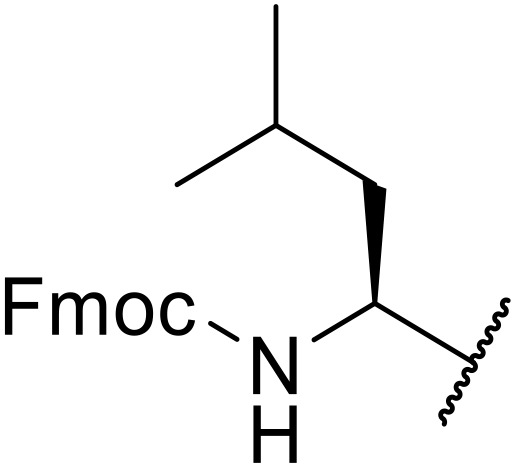	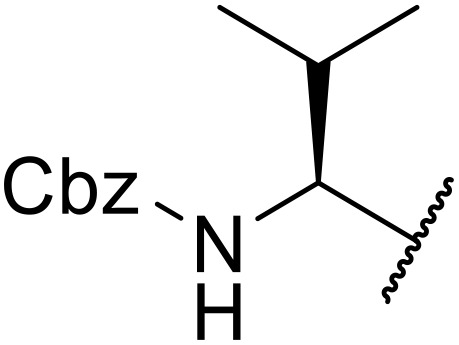	86%
3	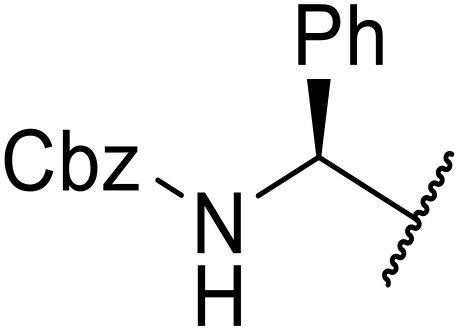	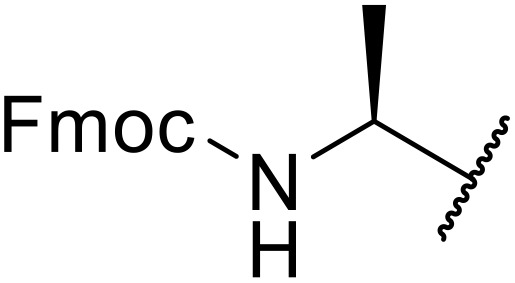	93%
4	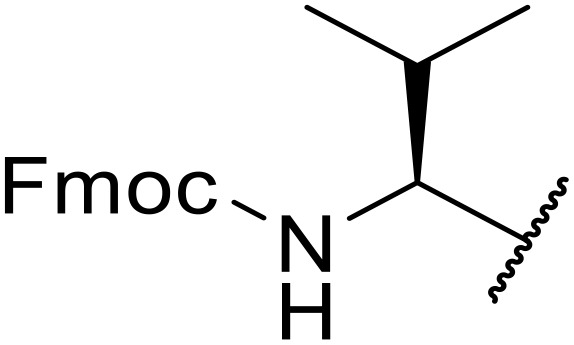	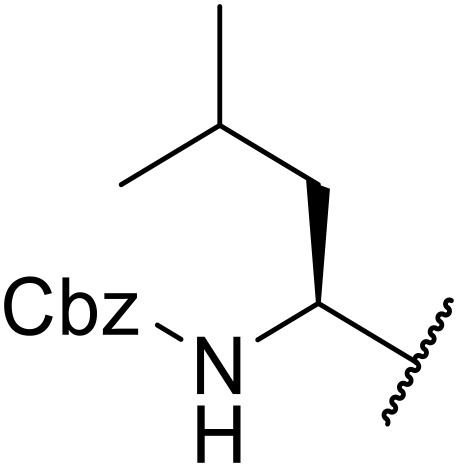	89%
5	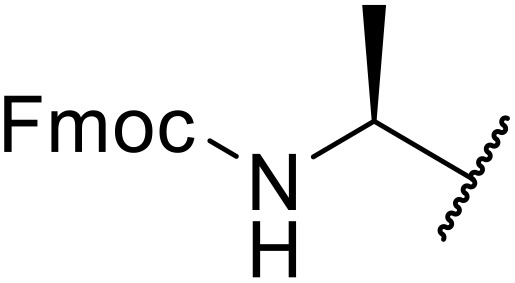	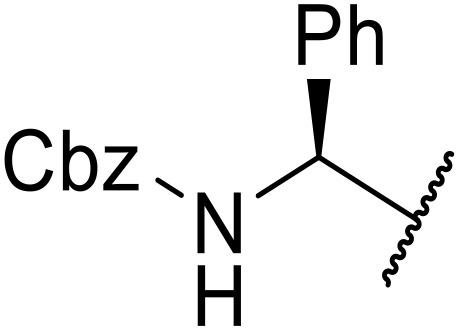	93%
6	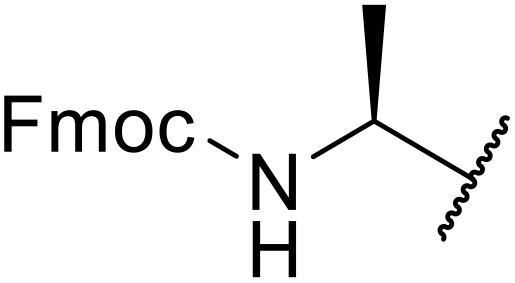	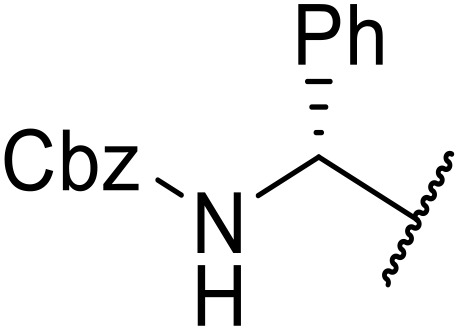	92%
7	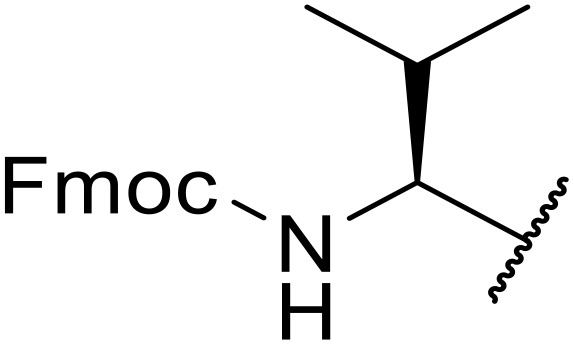	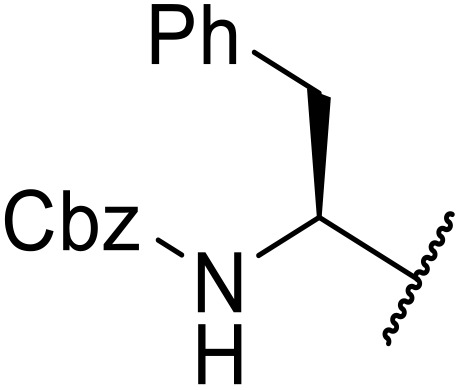	90%
8	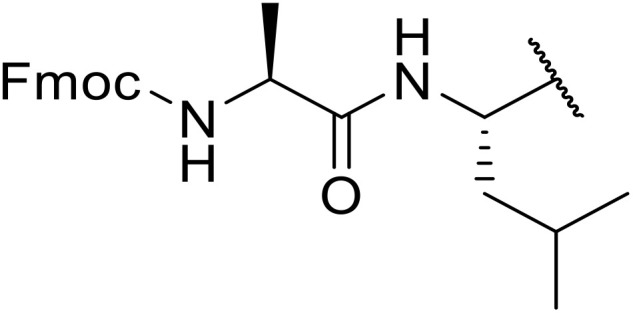	Ph	82%
9	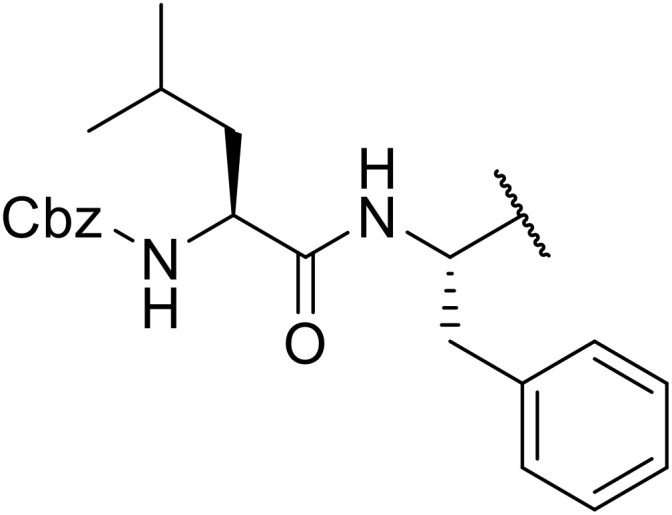	Ph	85%
10	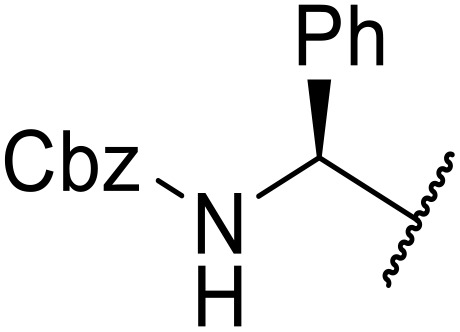	Ph	94%
11	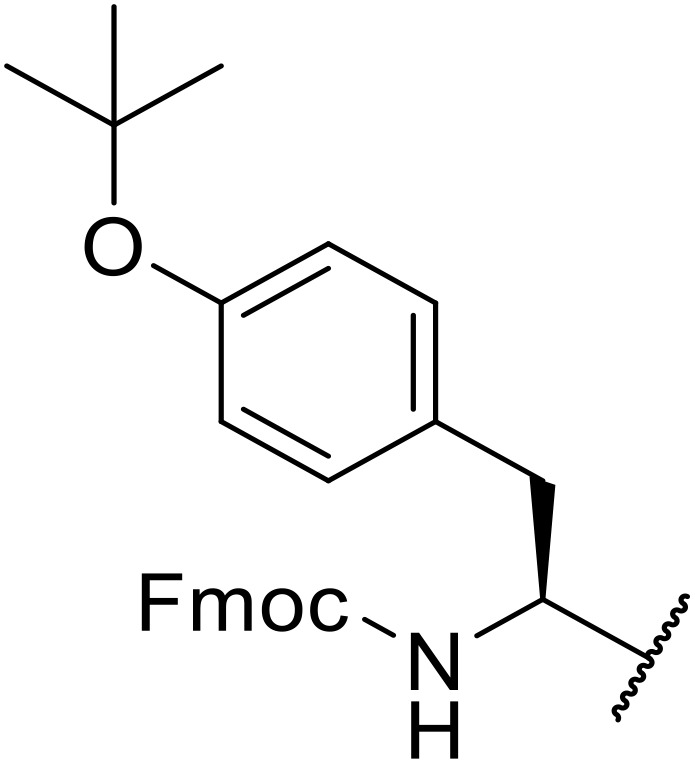	Ph	86%
12	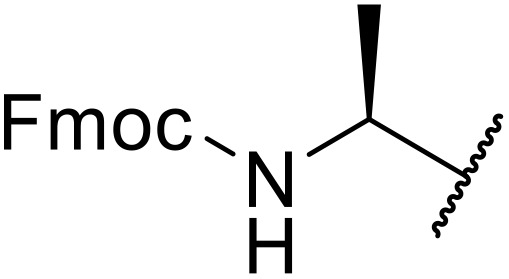	4-MeC_6_H_4_	90%
13	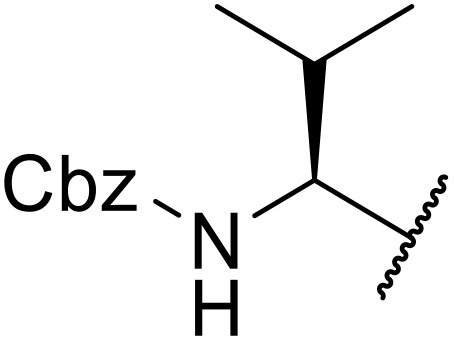	4-MeC_6_H_4_	92%
14	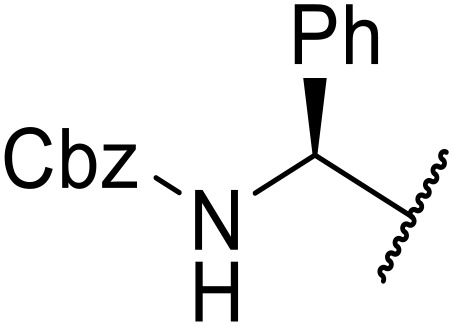	4-MeC_6_H_4_	95%
15	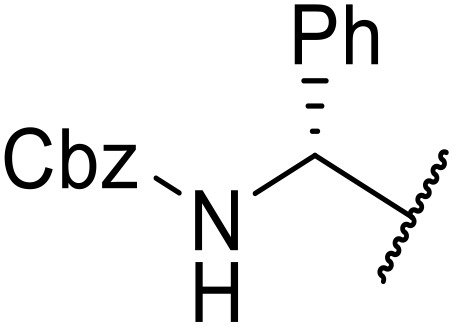	4-MeC_6_H_4_	94%
16	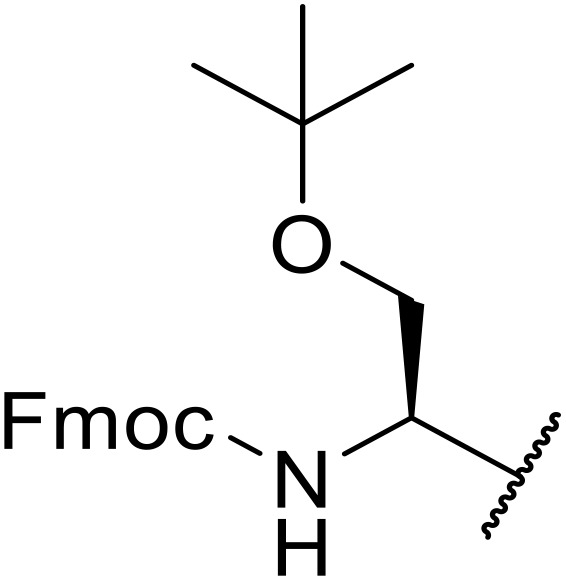	Dansyl	88%
17	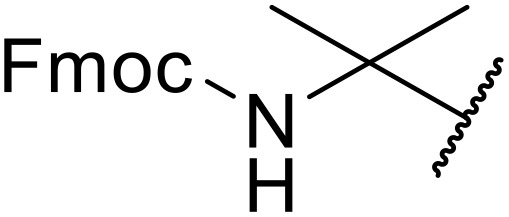	2,4-F_2_C_6_H_3_	90%
18	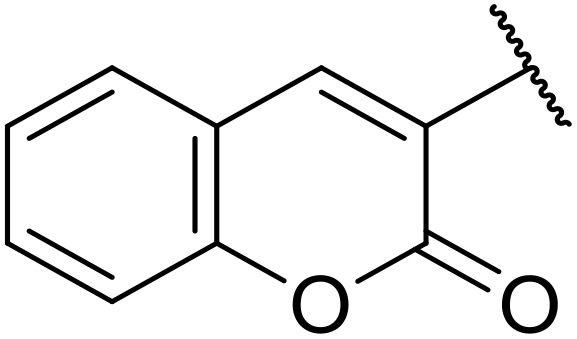	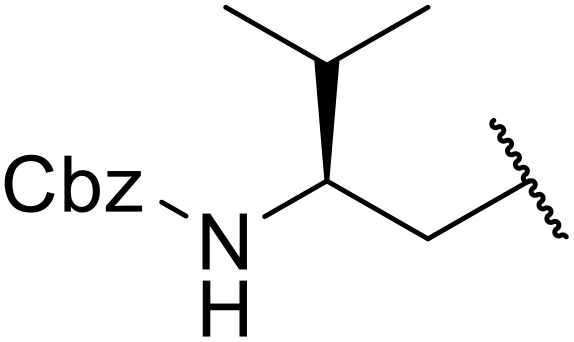	82%
19	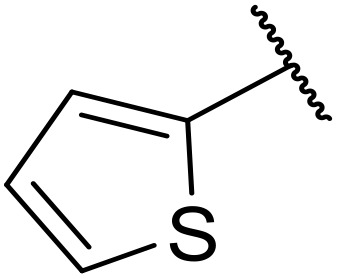	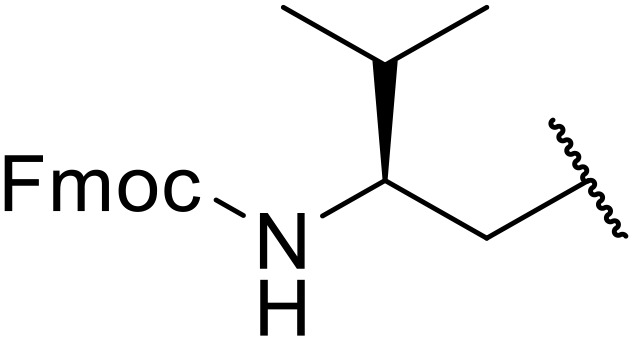	85%

**Scheme 11:**
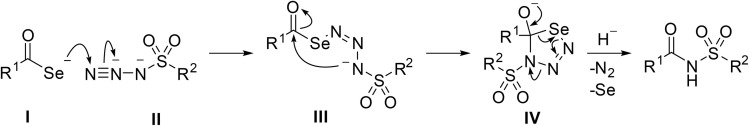
Possible mechanism for *N*-acyl sulfonamide formation.

The authors suggest that the mechanism proceeds *via* the formation of selenocarboxylate I by *in situ* selenation of the corresponding acid (Scheme 11). This intermediate then reacts with sulfonyl azide II through stepwise linear coupling, followed by intramolecular cyclisation to form a five-membered heteroaromatic ring IV. This decomposes through retro-[3 + 2] reaction, expelling nitrogen and selenium to furnish the *N*-acyl sulfonamide.

**Scheme 12:**
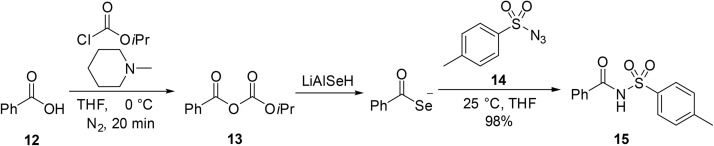
One-pot amidation of carboxylic acids.

Hu *et al.* reported an efficient, one-pot amidation of carboxylic acids *via* selenocarboxylate intermediates.^75^ While the major focus of the paper is on the preparation of amides, one example is provided of an *N*-acyl sulfonamide 15 obtained from carboxylic acid 12 and sulfonyl azide 14 in 98% overall yield (Scheme 12).

**Scheme 13:**
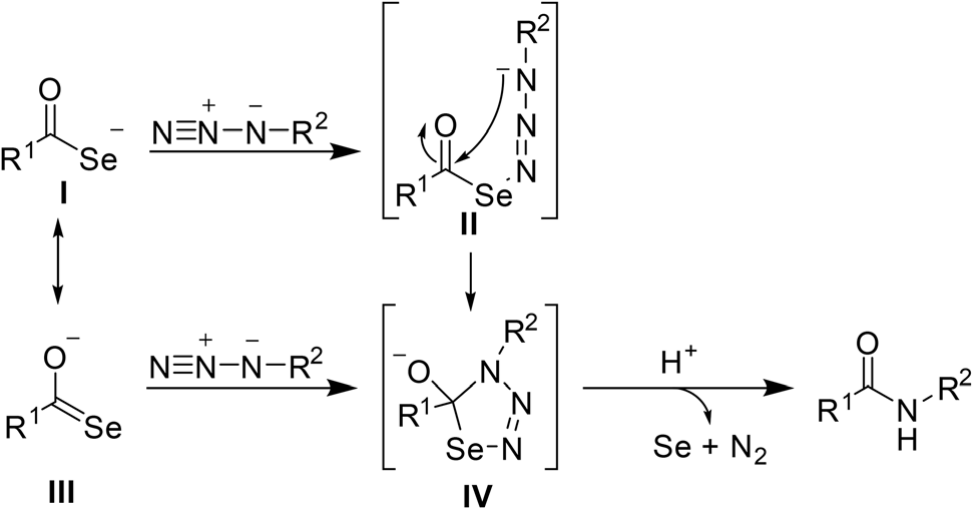
Proposed mechanism for selenocarboxylate/azide amidation.

Two pathways have been proposed by which selenatriazoline intermediate IV can be generated (Scheme 13). Path A is a stepwise mechanism in which the terminal nitrogen of the electron-deficient azide combines with the selenium of selenocarboxylate I followed by an intramolecular cyclisation to form intermediate II. Electron-withdrawing groups help stabilise the transition state by delocalising the negative charge on the nitrogen. Alternatively, the reaction may proceed *via* electron-rich azides (path B), which involves a slower, concerted [3 + 2] cyclisation.

Zeng *et al.* investigated several metal-based Lewis acid catalysts for the inter- and intramolecular *N*-acylation of sulfonamides with esters.^76^ A catalytic loading of 1.5 equivalents of TiCl_4_ in 1,1,2,2-tetrachloroethane at 115–160 °C in a sealed tube proved optimal (Table 28). This protocol was suitable for aliphatic (entries 1–10), aromatic (entries 11–13 and 16), and heteroaromatic (entry 14) esters, in addition to both aromatic (entries 1–15) and aliphatic sulfonamides (entry 16). The group postulated that the 4-methoxy derivative was obtained in poor yield (30%) due to the formation of an unreactive oxonium salt (entry 5). To prevent this, addition of 1.5 equivalents of triethylamine saw the yield increase by 24%. Bulky esters returned lower yields (entries 9 and 10). Varying the electronic nature of the ester substituents did not significantly impact upon reactivity (entries 11–14). An interesting intramolecular transformation of 2-sulfamoyl benzoic acid ethyl ester to saccharin proceeded in 82% yield at 115 °C (entry 15).

**Table 28:** TiCl_4_-mediated *N*-acylation of sulfonamides with esters

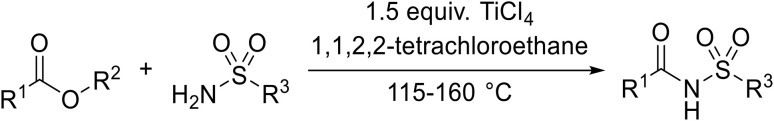					
Entry	R^1^	R^2^	R^3^	Time (h)	Yield
1	Me	Et	4-MeC_6_H_4_	18	76%
2	Me	Et	4-ClC_6_H_4_	30	81%
3	Me	Et	4-BrC_6_H_4_	55	77%
4	Me	Et	4-O_2_NC_6_H_4_	48	48%
5	Me	Et	4-MeOC_6_H_4_	36	30% (54%)^a^
6	Me	Et	Ph	24	70%
7	Me	Et	4-MeC_6_H_4_	24	69%
8	Me	^*n*^Bu	4-MeC_6_H_4_	24	72%
9	Me	^*t*^Bu	4-MeC_6_H_4_	24	56%
10	^*i*^Pr	Et	4-MeC_6_H_4_	24	51%^b^
11	Ph	Et	4-MeC_6_H_4_	24	97%^c^
12	4-MeC_6_H_4_	Et	4-MeC_6_H_4_	24	55%^c^
13	4-ClC_6_H_4_	Et	4-MeC_6_H_4_	24	46%^c^
14	2-Pyridyl	Et	4-MeC_6_H_4_	24	45%^c^
15	—	—	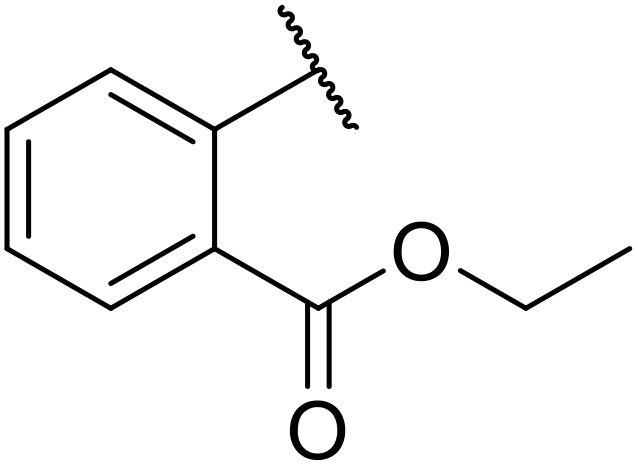	48	82%
16	Ph	Et	Me	24	94%^c^

a 1.5 equiv. triethylamine was added.

b 3 equiv. ethyl isobutyrate was added.

c Reaction carried out at 160 °C.

## 5. Thio acids

Williams *et al.* discovered that reaction of a benzylazide with thioacetic acid produced *N*-benzyl acetamide which could be applied to sulfonyl azides. Accordingly, reaction of thio acids with various sulfonyl azides in the presence of 2,6-lutidine provided easy access to *N*-acyl sulfonamides (Table 29).^77^ Thiobenzoic acid and thioacetic acid readily combined with tosyl azide (entries 1 and 2) or 4-carboxysulfonyl azide in excellent yields (entries 3 and 4). α-Aminoacyl sulfonamide derivatives could be obtained from the corresponding 2,4,6-trimethoxybenzyl (2,4,6-TMOB) thioesters by initial liberation of the thio acid, followed by reaction with the sulfonyl azide (entries 5–9).

**Table 29:** Preparation of acyl sulfonamides from thio acids/esters and sulfonyl azides^a^

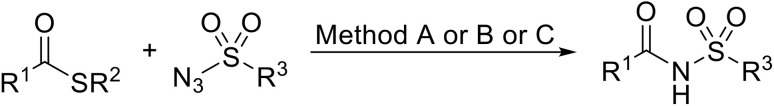						
Entry	R^1^	R^2^	R^3^	Method	Time	Yield
1	Ph	H	Ph	A	15 min	98%
2	Me	H	Ph	A	15 min	96%
3	Ph	H	4-(HO_2_C)C_6_H_4_	B	1 h	93%
4	Me	H	4-(HO_2_C)C_6_H_4_	B	1 h	98%
5	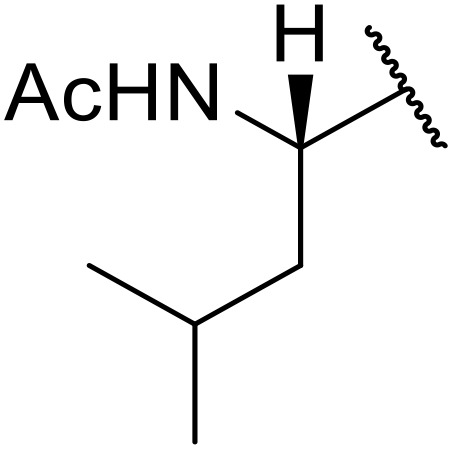	2,4,6-TMOB	Ph	C	12 h	91%
6	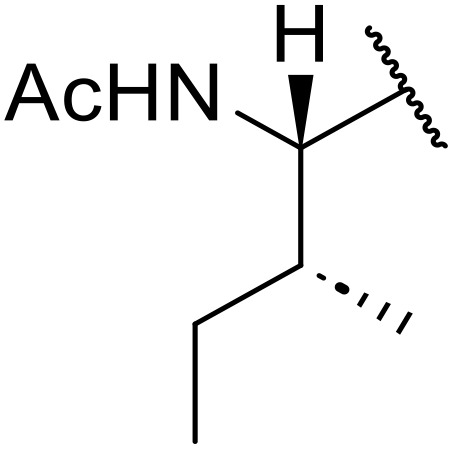	2,4,6-TMOB	4-MeC_6_H_4_	C	12 h	87%
7	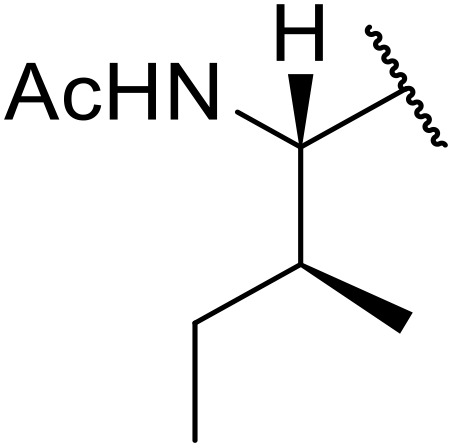	2,4,6-TMOB	4-MeC_6_H_4_	C	12 h	72%
8	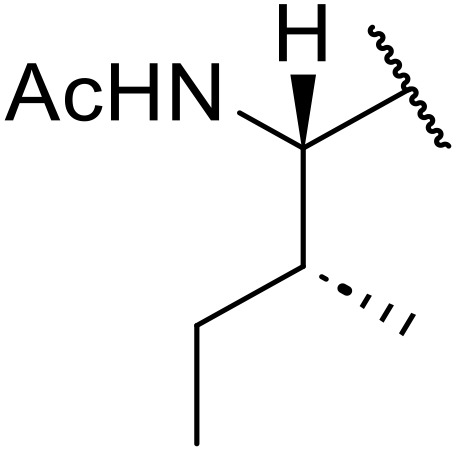	2,4,6-TMOB	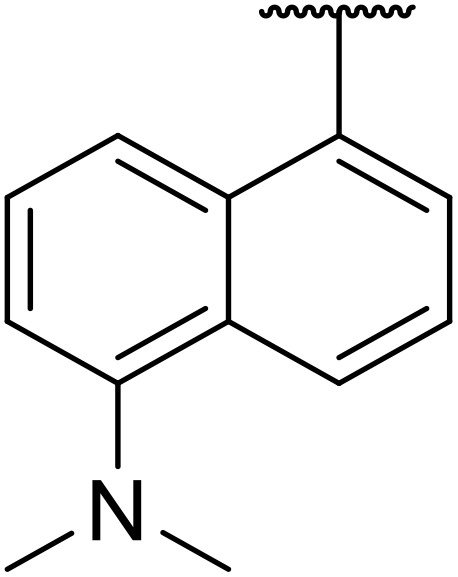	C	12 h	73%
9	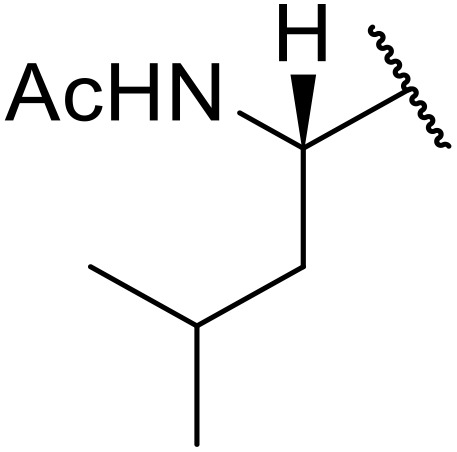	2,4,6-TMOB	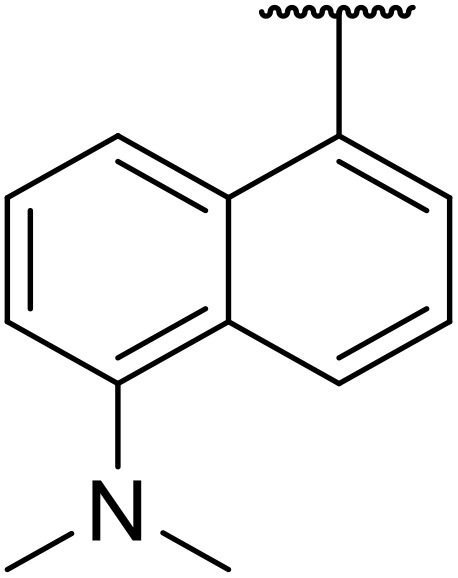	C	12 h	73%

a Method A: 2,6-lutidine, methanol, r.t.; method B: 2,6-lutidine, water, r.t.; method C: TFA/DCM (40–80% v/v), HSiEt_3_, r.t. for 1–3 h, then 2,6-lutidine, MeOH, r.t.

**Scheme 14:**
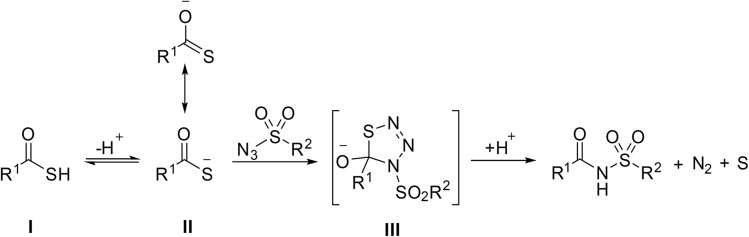
Reaction mechanism of thio acids with sulfonyl azides.

The mechanism is outlined in Scheme 14 and proceeds *via* the formation of thiatriazoline intermediate III. Reaction of the thio acid with sulfonyl azide forms a thiatriazoline intermediate through either a stepwise diazo transfer-like mechanism or a [3 + 2] cycloaddition. Decomposition of thiatriazoline intermediate III*via* a retro [3 + 2] cycloelimination ultimately yields the acyl sulfonamide, with the concomitant release of nitrogen and sulfur.

Liskamp exploited a similar methodology for the preparation of *N*-β-protected acyl sulfonamides (Table 30).^78^ Both Fmoc- and Cbz-protected derivatives of glycine (entries 1–4), phenylalanine (entries 5–8), valine (entries 9 and 10), and serine (entries 11 and 12) were coupled to either thioacetic or thiobenzoic acid in excellent yields. While chloroform was suitable for glycine substrates (entries 1–4), DMF was required for less soluble starting materials (entries 5–12). Significantly, this approach also facilitated chemical ligation, allowing access to (α-amino)acyl sulfonamides (entry 13) or peptidyl sulfonamides (entry 14).

**Table 30:** Synthesis of *N*-β-protected acyl sulfonamides

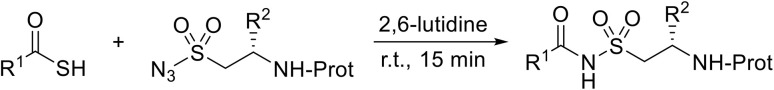					
Entry	Protecting group	R^1^	R^2^	Solvent	Yield
1	Fmoc	Me	H	CHCl_3_	90%
2	Fmoc	Ph	H	CHCl_3_	100%
3	Cbz	Me	H	CHCl_3_	100%
4	Cbz	Ph	H	CHCl_3_	100%
5	Fmoc	Me	Bn	DMF	91%
6	Fmoc	Ph	Bn	DMF	87%
7	Cbz	Me	Bn	DMF	95%
8	Cbz	Ph	Bn	DMF	96%
9	Fmoc	Me	^*i*^Pr	DMF	95%
10	Fmoc	Ph	^*i*^Pr	DMF	96%
11	Fmoc	Me	^*t*^BuO-CH_2_	DMF	93%
12	Fmoc	Ph	^*t*^BuO-CH_2_	DMF	94%
13	Fmoc	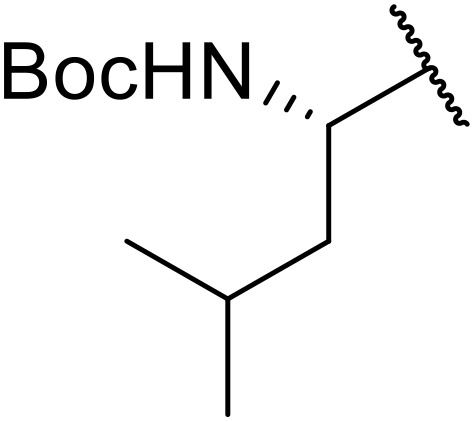	H	DMF	81%
14	Cbz	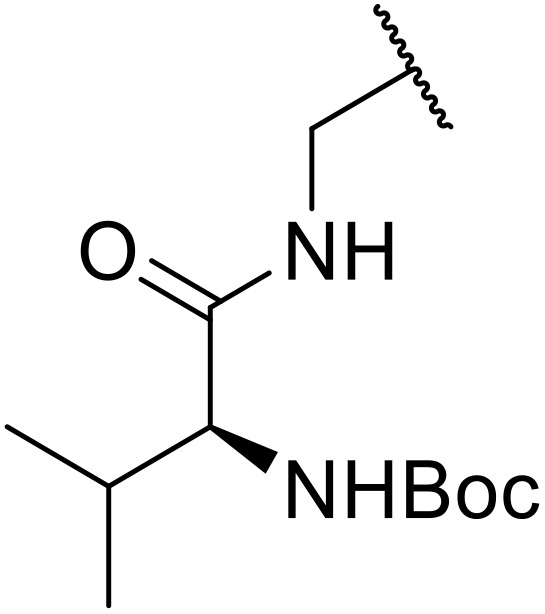	Bn	DMF	99%

## 6. Aldehydes and ketones

Ma *et al.* designed a protocol for the direct *N*-acylation of sulfonamides using aldehydes and azolium salts (Table 31).^79^ A combination of azolium salt 16 (2-mesityl-6,7-dihydro-5*H*-pyrrolo[2,1-*c*][1,2,4]triazol-2-ium chloride), oxidant DPQ (3,3′,5,5′-tetra-*tert*-butyldiphenoquinone) and potassium *tert*-butoxide facilitated the coupling of selected aldehydes to tosylamide to generate the desired *N*-tosylcarboxamides in excellent yields (entries 1–11). Aromatic (entries 1–5), thiophenic (entry 6) and naphthalic (entry 7) aldehydes reacted readily, in addition to enals (entry 8) and ynals (entries 9–11). Changing the sulfonamide substituent to phenyl (entry 12), 3-nitrophenyl (entry 13), 2-chlorophenyl (entry 14) or methyl (entry 15) was similarly successful.

**Table 31:** Oxidative *N*-acylation of sulfonamides using aldehydes

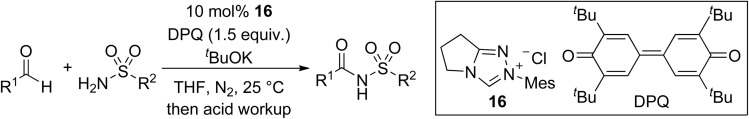			
Entry	R^1^	R^2^	Yield
1	4-ClC_6_H_4_	4-MeC_6_H_4_	97%
2	Ph	4-MeC_6_H_4_	97%
3	2-FC_6_H_4_	4-MeC_6_H_4_	94%
4	3-MeOC_6_H_4_	4-MeC_6_H_4_	90%
5	3-O_2_NC_6_H_4_	4-MeC_6_H_4_	87%
6	2-Thienyl	4-MeC_6_H_4_	87%
7	2-Naphthyl	4-MeC_6_H_4_	91%
8	(*E*)-Ph–CH=CH	4-MeC_6_H_4_	82%
9	Heptyne	4-MeC_6_H_4_	87%
10	Cy–C≡C	4-MeC_6_H_4_	79%
11	Ph–C≡C	4-MeC_6_H_4_	71%
12	4-ClC_6_H_4_	Ph	95%
13	4-ClC_6_H_4_	3-O_2_NC_6_H_4_	93%
14	4-ClC_6_H_4_	2-ClC_6_H_4_	96%
15	4-ClC_6_H_4_	Me	92%
16	2,3-Cl_2_C_6_H_3_	5-Br-2-Thienyl	87%^a^

a 2.0 equiv. ^*t*^BuOK used.

**Scheme 15:**
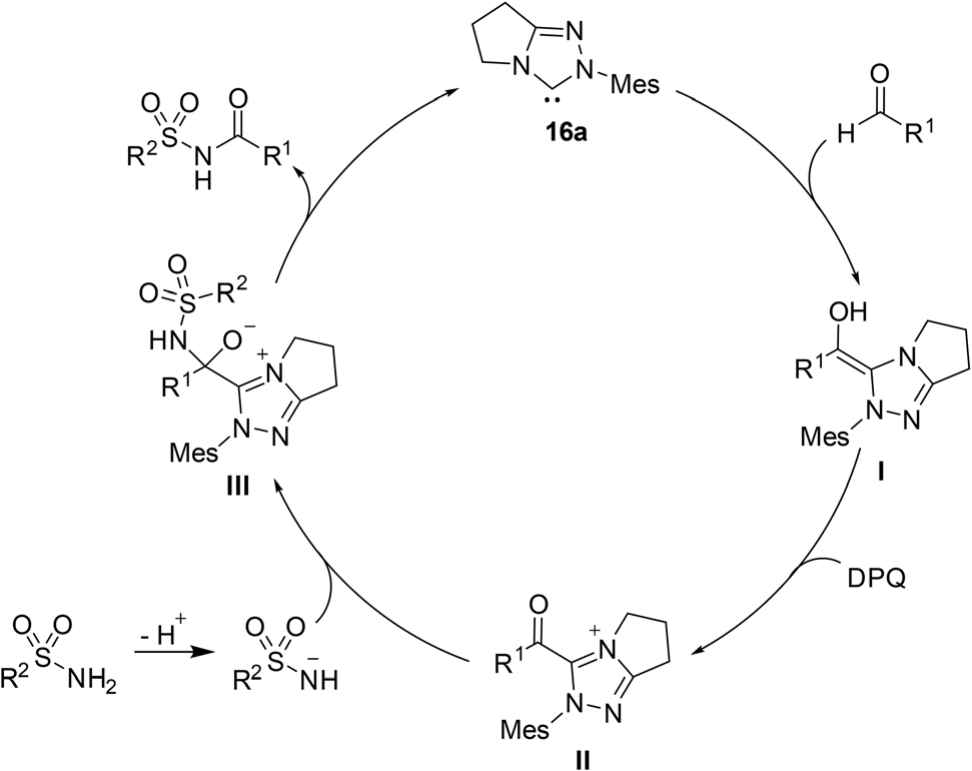
Catalytic cycle.

The catalytic cycle starts with carbene 16a which undergoes nucleophilic addition to the aldehyde to afford Breslow intermediate I (Scheme 15). This intermediate is then oxidised by DPQ to generate acylazolium species II. In tandem, ^*t*^BuOK deprotonates the sulfonamide which then attacks the carbonyl forming adduct III. Subsequent fragmentation of the azolium intermediate regenerates carbene 16a and furnishes the target *N*-acyl sulfonamide.

Chang and Chan developed the first chemoselective ruthenium-catalysed amidation of aldehydes using *N*-(*para*-tolylsulfonyl)imino phenyliodinane (PhI=NTs) as the nitrogen source (Table 32).^80^ The procedure was compatible with tertiary carbon-containing substrates, with C–N bond formation occurring selectively at the aldehyde functional group (entries 1, 2, 7 and 8). Competitive side reactions were not observed in the presence of benzylic (entries 10 and 11) or vinylic (entries 12 and 13) alkene bonds. Aromatic (entries 15–18) and heteroaromatic (entries 19 and 20) aldehydes were readily transformed to their corresponding *N*-acyl sulfonamides.

**Table 32:** [Ru(TTP)(CO)]-catalysed amidation

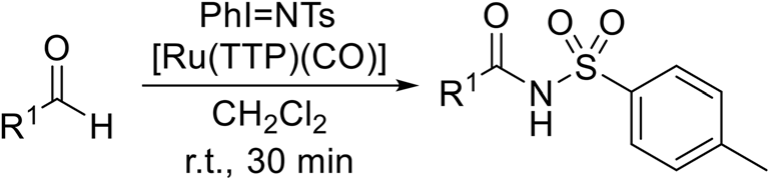		
Entry	R^1^	Yield
1	^*i*^Bu	94%
2	^*i*^Pr	86%
3	Et	97%
4	^*n*^Hex	97%
5	^*t*^Bu	60%
6	Br(CH_2_)_4_	96%
7	Cyclopropyl	91%
8	Cyclopentyl	99%
9	Cy	97%
10	Ph–CH_2_	86%
11	Ph–(CH_2_)_2_	68%
12	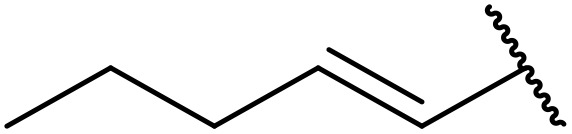	99%
13	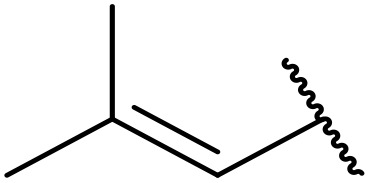	91%
14	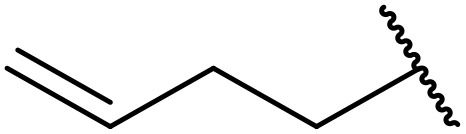	99%
15	Ph	93%
16	4-MeC_6_H_4_	92%
17	4-MeOC_6_H_4_	96%
18	1-Naphthyl	68%
19	2-Thienyl	76%
20	2-Furyl	93%

**Scheme 16:**
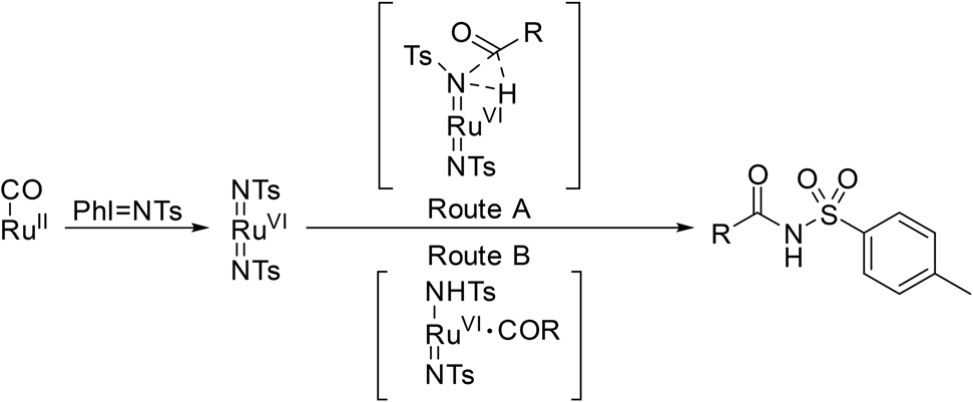
Proposed mechanism for Ru-catalysed amidation.

To elucidate the mechanism, deuterium-labelling experiments were performed using α-[d]-benzaldehyde. *N*,*N*-[d]-Tosylbenzamide was isolated in 90% yield with 76% deuterium incorporation at the nitrogen atom (Scheme 16). The reaction likely proceeds initially *via* a high-oxidation-state metal complex [Ru(TTP)(NTs)_2_], with subsequent transfer of the imido/nitrene group either by direct insertion (route A) or by H-atom abstraction/radical rebound (route B) to generate the sulfonamide adduct.

Johann Chan and co-workers established a mild method for preparing *N*-sulfonylcarboxamides from aldehydes under rhodium(ii) catalysis (Table 33).^81^ The best yields were obtained using a Rh_2_(esp)_2_ catalyst in isopropyl acetate with PhI(OC(O)^*t*^Bu)_2_ as the oxidant. The procedure had excellent functional group tolerability with successful coupling to halo- (entries 4–7, 9 and 12) and nitro-containing (entry 8, 10 and 11) aryl sulfonamides. Similarly, good results were recorded with heterocyclic (entries 7 and 8) and activated olefinic aldehydes (entry 12). Lower temperatures were required for some electron-rich sulfonamides to reduce their tendency to degrade (entries 1–3).

**Table 33:** Rh(ii)-catalysed oxidative sulfamidation of aldehydes

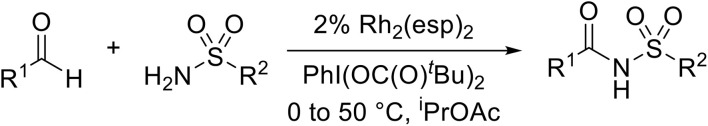				
Entry	R^1^	R^2^	*T* (°C)	Yield
1	Ph	Ph	0	94%
2	Ph	4-MeC_6_H_4_	0	90%
3	Mesityl	Me	0	90%
4	Ph	2-Cl-4-BrC_6_H_3_	50	80% (99%)
5	4-MeOC_6_H_4_	2-Cl-4-BrC_6_H_3_	50	98%
6	4-NCC_6_H_4_	2-Cl-4-BrC_6_H_3_	50	85% (99%)
7	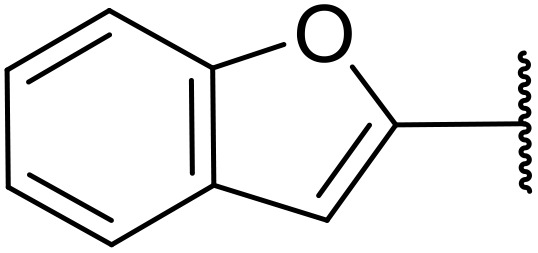	3-F_3_CC_6_H_4_	50	72%
8	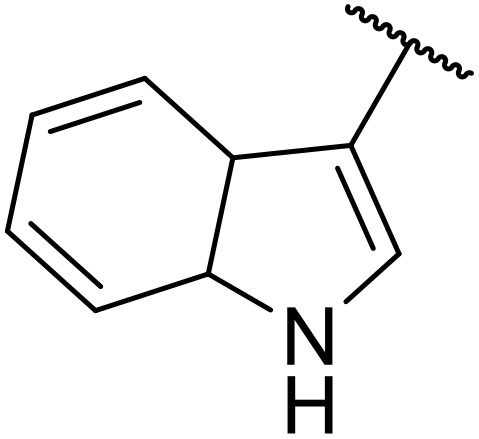	3-O_2_NC_6_H_4_	50	72%
9	^*t*^Bu	2-Cl-4-BrC_6_H_3_	50	67%
10	Cyclopropyl	3-O_2_NC_6_H_4_	50	62%
11	^*n*^Pr	3-O_2_NC_6_H_4_	50	85%
12	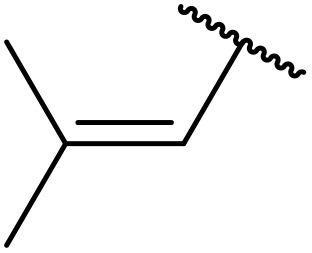	3-BrC_6_H_4_	50	63%

**Scheme 17:**
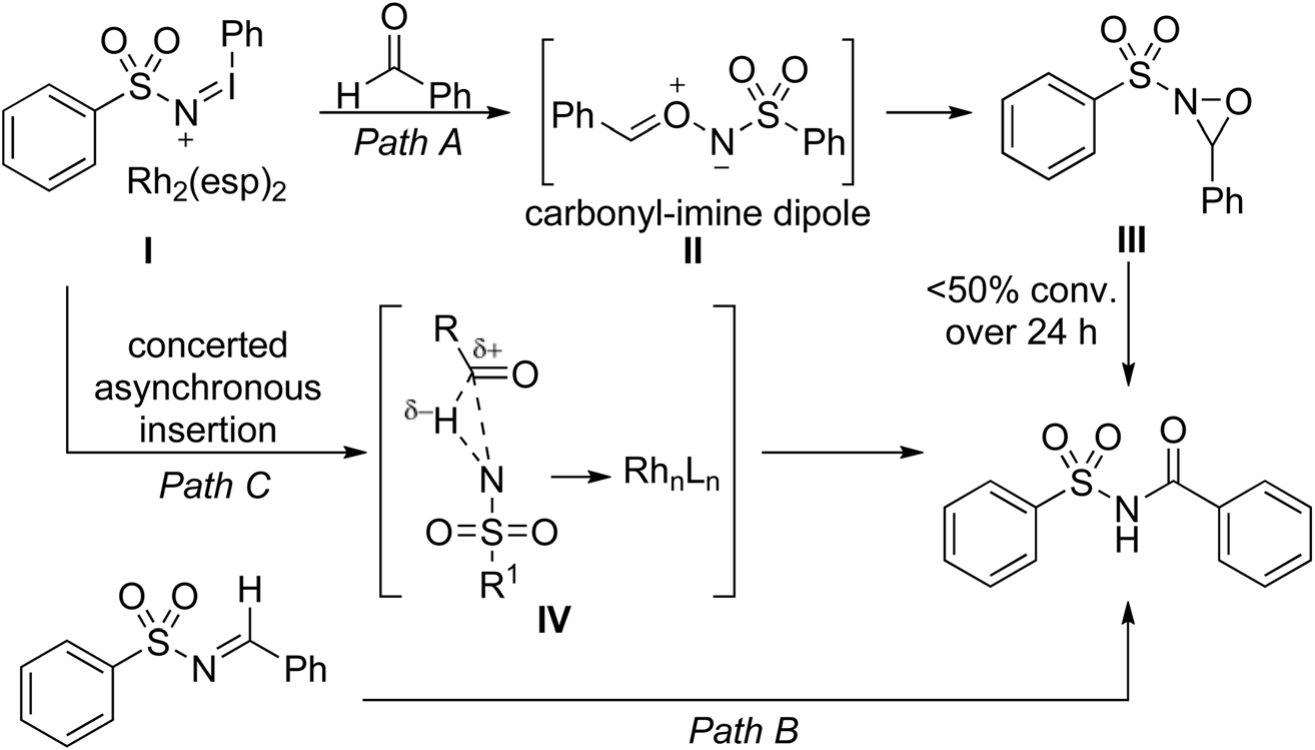
Potential pathways for Rh catalysed sulfamidation of aldehydes.

To determine the most likely mechanistic pathway, the authors carried out several rate experiments (Scheme 17). Reaction of a sulfonamide with an equimolar mixture of benzaldehyde and benzaldehyde-*d*_6_ was accompanied by a primary isotope effect (*k*_H_/*k*_D_ = 2.5), indicating that C–H bond cleavage was occurring during the rate determining step. Given this and other supporting evidence, the most plausible route is path C which proceeds *via* a concerted asynchronous nitrene insertion into an aldehyde hydrogen.

A catalyst-free process for the sulfamidation of aldehydes has been described by Phukan and co-workers employing *N*,*N*-dibromo-*p*-toluene sulfonamide (TsNBr_2_) as a nitrene transfer source (Table 34).^82^ A series of aromatic (entries 1–11) and aliphatic (entries 12–14) aldehydes were treated with TsNBr_2_ and potassium carbonate to generate the corresponding *N*-acyl sulfonamides in excellent yields (79–90%) in 4 hours or less. The mechanism proceeds *via* base-mediated abstraction of Br^+^ ions with subsequent loss of KBr to form a sulfonyl nitrene intermediate which undergoes C–H σ-insertion to generate the final product.

**Table 34:** Sulfamidation of aldehydes using TsNBr_2_

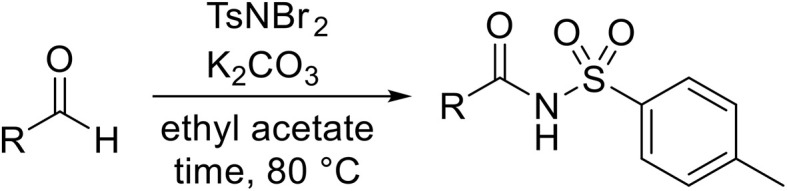			
Entry	R	Time (h)	Yield
1	Ph	3	85%
2	4-ClC_6_H_4_	3	84%
3	4-BrC_6_H_4_	3	80%
4	4-MeOC_6_H_4_	3	88%
5	2-BrC_6_H_4_	3	78%
6	2-FC_6_H_4_	3	80%
7	2-MeOC_6_H_4_	3	88%
8	3-ClC_6_H_4_	3	84%
9	3-BrC_6_H_4_	3	80%
10	4-MeC_6_H_4_	3	89%
11	2-Naphthyl	3	90%
12	CH_3_(CH_2_)_6_	3	89%
13	^*i*^Pr	4	79%
14	Ph(CH_2_)_2_	4	87%

Phukan subsequently extended this protocol to α-nitroketones (Table 35).^83^ Higher yields were obtained from *para*- and *meta*-substituted aryl ketones (entries 2–8), whereas *ortho*-substituted ketones were less reactive (entries 9–11). The transformation of aliphatic ketones was also successful, albeit in lower yields (entries 13 and 14). Typically, α-nitroketones are stable in the β-hydroxy nitroolefin form due to stabilisation by intramolecular hydrogen bonding during enolization. Treatment with base effects the abstraction of Br^+^ from TsNBr_2_, leading to nucleophilic attack of the nitrogen on the α-nitroketone. Subsequent protonation-deprotonation in water results in elimination of nitromethane and formation of the tosyl amide product.

**Table 35:** Sulfamidation of nitroketones using TsNBr_2_

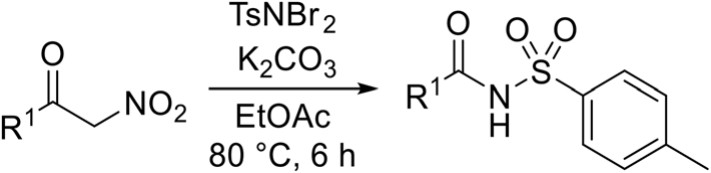		
Entry	R^1^	Yield
1	Ph	76%
2	4-BrC_6_H_4_	71%
3	4-ClC_6_H_4_	74%
4	4-MeC_6_H_4_	76%
5	4-MeOC_6_H_4_	72%
6	3-ClC_6_H_4_	68%
7	3-BrC_6_H_4_	66%
8	3-O_2_NC_6_H_4_	65%
9	2-MeOC_6_H_4_	53%
10	2-FC_6_H_4_	57%
11	2-BrC_6_H_4_	56%
12	2-Naphthyl	70
13	Ph(CH_2_)_2_	65%
14	^*n*^Heptyl	61%

## 7. Pd-catalysed aminocarbonylations

**Scheme 18:**
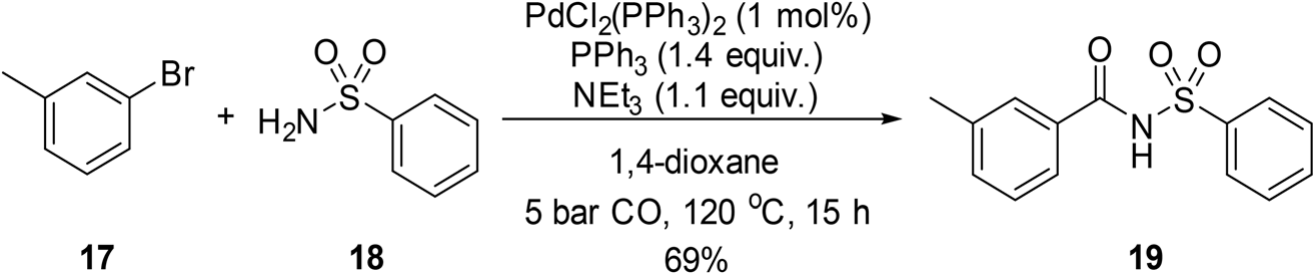
Pd-catalysed aminocarbonylation of 1-bromo-3-methylbenzene.

In pioneering work on Pd-catalysed carbonylations, Indolese and Schnyder demonstrated how aminocarbonylation of aryl bromides with sulfonyl azides could provide access to acyl sulfonamides.^84^ Reaction of 1-bromo-3-methylbenzene with benzenesulfonamide in the presence of PdCl_2_(PPh_3_)_2_, PPh_3_ and triethylamine in a carbon monoxide atmosphere afforded 3-methyl-*N*-(phenylsulfonyl)benzamide in 70% conversion and 69% yield (Scheme 18). By increasing the equivalents of triethylamine from 1.1 to 2, 100% conversion was achieved.

A robust Pd-catalysed aminocarbonylation method for the preparation of *N*-acyl sulfonamides using molybdenum hexacarbonyl, which generates carbon monoxide *in situ*, has been developed by Larhed and co-workers (Table 36).^85^ With the appropriate sulfonamide and aryl iodide in the presence of Mo(CO)_6_, 10 mol% palladium acetate, and DBU in a sealed tube, carbonylations reached completion in 15 minutes under microwave irradiation (method A). Various aryl iodides were coupled to tosyl azide in high yields (entries 1–7, 10 and 11). Heteroaryl halides were also compatible substrates (entries 10 and 11). Coupling to other primary aromatic and aliphatic sulfonamides (entries 12–15) was similarly successful, while reactions with secondary sulfonamides was likewise possible, although in reduced yields (entry 16). The reduced reactivity of aryl bromides necessitated a switch to Herrmann's palladacycle^86^ in combination with [(^*t*^Bu)_3_ PH]BF_4_ ^87^ at higher temperature (method B). Gratifyingly, the resulting yields were higher than those obtained from the matching aryl iodides (entries 1–5, 7, 10, 14 and 15).

**Table 36:** Pd catalysed carbonylation of aryl halides with sulfonamides in the presence of Mo(CO)_6_

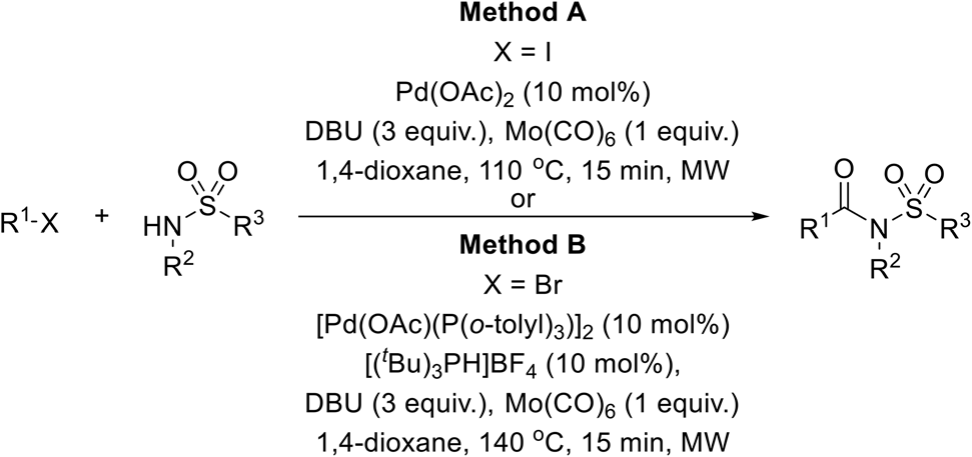					
Entry	R^1^	R^2^	R^3^	Yield	
				Method A	Method B
1	4-MeOC_6_H_4_	H	4-MeC_6_H_4_	88%	93%
2	4-MeC_6_H_4_	H	4-MeC_6_H_4_	87%	91%
3	2-MeC_6_H_4_	H	4-MeC_6_H_4_	88%	93%
4	Ph	H	4-MeC_6_H_4_	80%	95%
5	1-Naphthyl	H	4-MeC_6_H_4_	74%	96%
6	4-PhCO–C_6_H_4_	H	4-MeC_6_H_4_	70%	
7	4-F_3_CC_6_H_4_	H	4-MeC_6_H_4_	76%	95%
8	2-MeOC_6_H_4_	H	4-MeC_6_H_4_		94%
9	4-NCC_6_H_4_	H	4-MeC_6_H_4_		83%
10	2-Thienyl	H	4-MeC_6_H_4_	65%	79%
11	3-Thienyl	H	4-MeC_6_H_4_	79%	
12	4-MeC_6_H_4_	H	Ph	88%	
13	4-MeC_6_H_4_	H	4-BrC_6_H_4_	84%	
14	4-MeC_6_H_4_	H	Me	72%	88%
15	4-MeC_6_H_4_	H	CF_3_	71%	80%
16	4-MeC_6_H_4_	Me	Ph	47%	
17	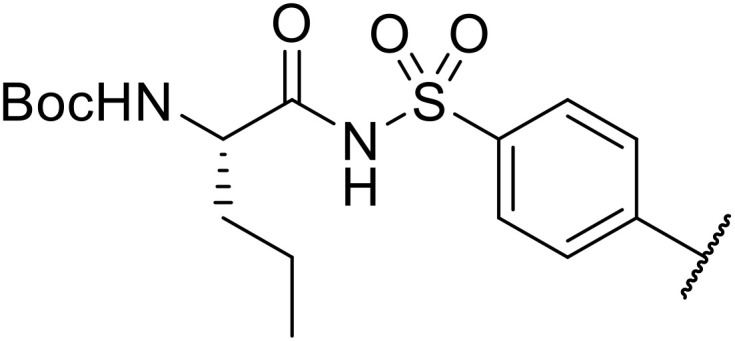	H	Me		52%

**Scheme 19:**
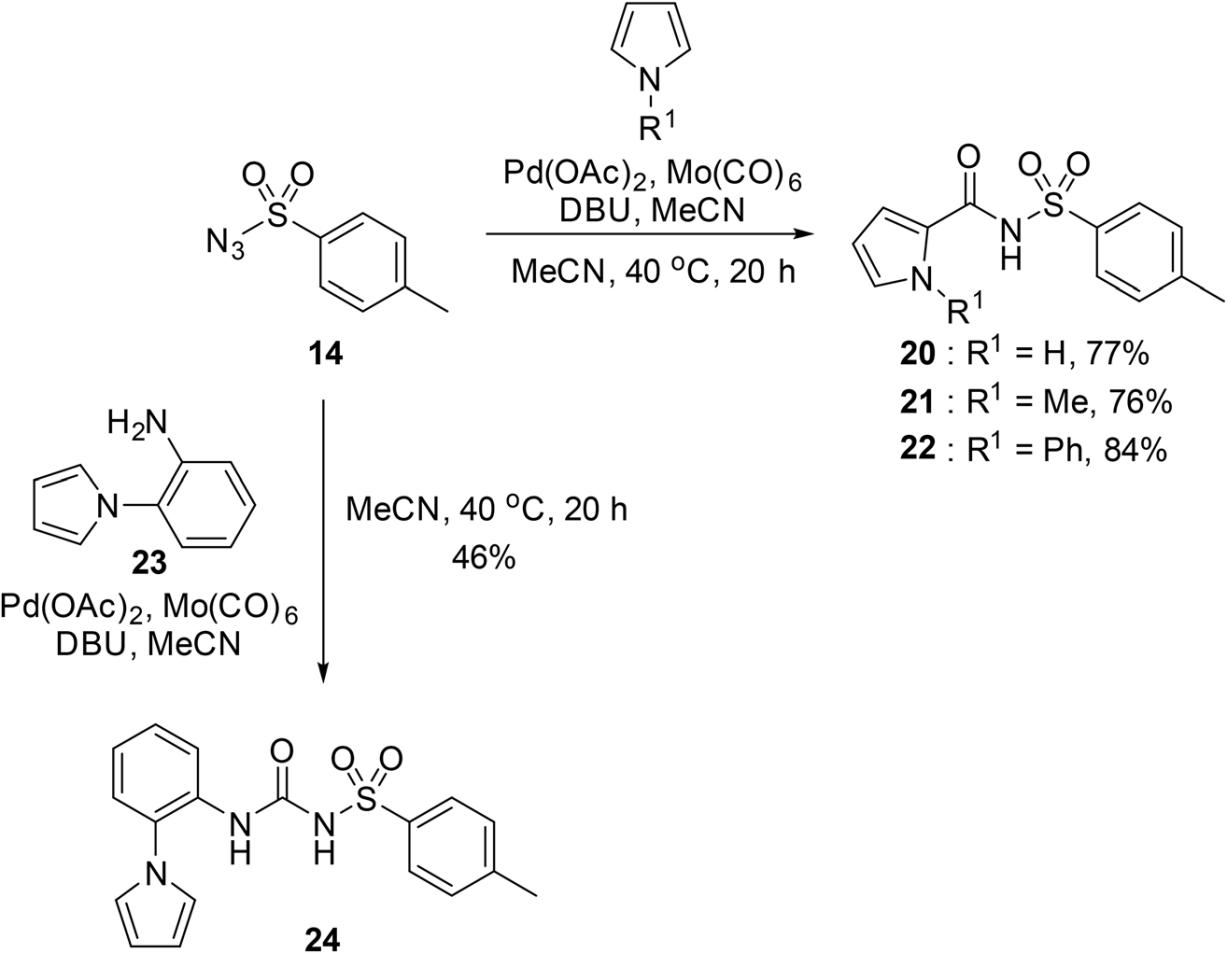
Regioselective aminocarbonylation of pyrroles.

Odell *et al.* demonstrated a mild method for the regioselective Pd-catalysed aminocarbonylation of substituted indoles and pyrroles with sulfonyl azides in the presence of Mo(CO)_6_ (Table 37).^88^ Carbonylation of differently substituted indoles in anhydrous acetonitrile proceeded mostly in high yields (entries 1–17), apart from electron-poor substrates, such as acyl- (entry 4), nitro- (entry 16) and nitrile-containing (entry 17) derivatives. Attempted transformation of 7-aza indole failed, even under forcing conditions. Both electron-rich (entries 18–22) and electron-poor (entries 23–27) sulfonyl azides reacted readily, with two exceptions. The steric bulk of two *ortho*-isopropyl blocked reaction completely (entry 20), while a *para*-brominated sulfonyl azide (entry 23) was also unreactive. *N*-Acyl sulfonamides were accessible from heteroaromatic sulfonyl azides (entries 28–30) but not in the case of pyridinesulfonyl azide (entry 31). Furthermore, pyrroles reacted with tosyl azide 14 under the same conditions to afford the corresponding *N*-acyl sulfonamides 20–22 in good yields, while carbonylation of ambident nucleophile 2-(1*H*-pyrrol-1-yl)aniline 23 resulted in the formation of *N*-carbamoyl sulfonamide 24 (Scheme 19).

**Table 37:** Regioselective Pd-catalysed aminocarbonylation of heteroaromatics

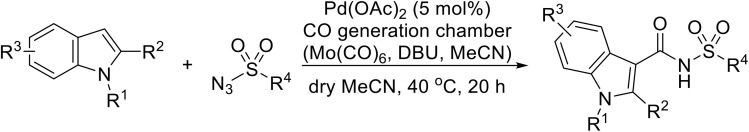					
Entry	R^1^	R^2^	R^3^	R^4^	Yield
1	Me	H	H	4-MeC_6_H_4_	95%
2	Bn	H	H	4-MeC_6_H_4_	89%
3	Ph	H	H	4-MeC_6_H_4_	68%
4	Ac	H	H	4-MeC_6_H_4_	n.r.
5	H	H	H	4-MeC_6_H_4_	95%
6	H	H	4-Me	4-MeC_6_H_4_	70%
7	Me	Me	H	4-MeC_6_H_4_	89%
8	Me	H	4-Me	4-MeC_6_H_4_	90%
9	Me	H	5-Me	4-MeC_6_H_4_	91%
10	Me	H	6-Me	4-MeC_6_H_4_	90%
11	Me	H	7-Me	4-MeC_6_H_4_	78%
12	Me	Ph	H	4-MeC_6_H_4_	84%
13	Me	H	5-MeO	4-MeC_6_H_4_	77%
14	Me	Me	4-MeO	4-MeC_6_H_4_	76%
15	Me	H	6-Cl	4-MeC_6_H_4_	79%
16	Me	H	5-NO_2_	4-MeC_6_H_4_	38%
17	Me	H	5-NC	4-MeC_6_H_4_	26%
18	Me	H	H	3,4-(MeO)_2_C_6_H_3_	75%
19	Me	H	H	4-MeOC_6_H_4_	71%
20	Me	H	H	2,4,6-^*i*^Pr_3_C_6_H_2_	n.r.
21	Me	H	H	4-NHAcC_6_H_4_	56%
22	Me	H	H	3-MeOC_6_H_4_	77%
23	Me	H	H	4-BrC_6_H_4_	Trace
24	Me	H	H	2,3,4-Cl_3_C_6_H_2_	73%
25	Me	H	H	3-F_3_CC_6_H_4_	92%
26	Me	H	H	4-NCC_6_H_4_	89%
27	Me	H	H	4-NO_2_C_6_H_4_	79%
28	Me	H	H	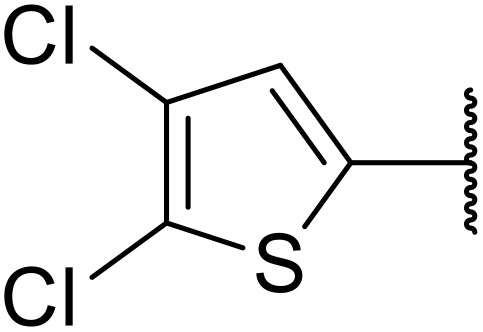	81%
29	Me	H	H	2-Thienyl	89%
30	Me	H	H	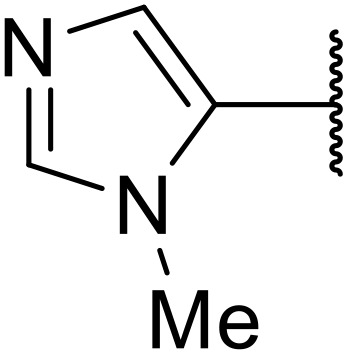	44%
31	Me	H	H	3-Pyridyl	Trace
32	Me	H	H	^*i*^Pr	73%
33	Me	H	H	^*n*^Bu	70%
34	Me	H	H	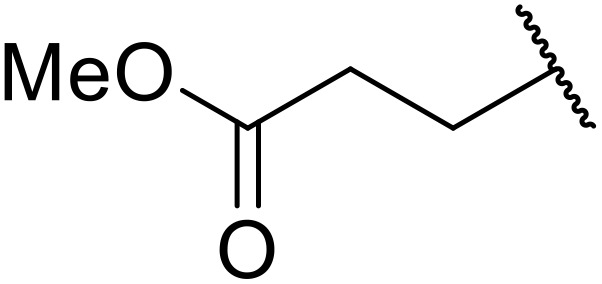	73%
35	Me	H	H	Bn	93%

**Scheme 20:**
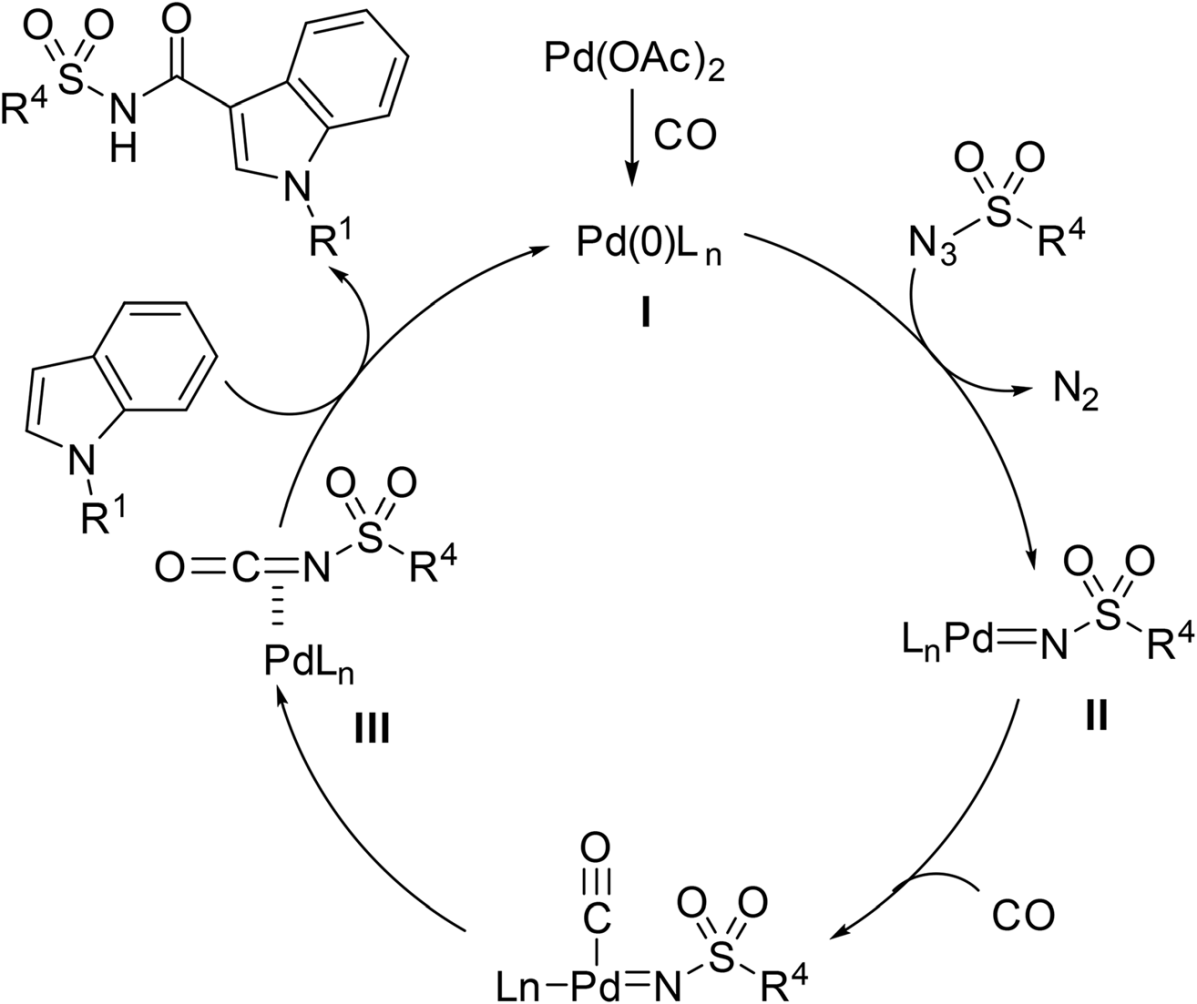
Proposed mechanism for heteroaryl aminocarbonylation.

According to the proposed mechanism (Scheme 20), the Pd(ii) precatalyst is reduced *in situ* to Pd(0) active complex I under carbon monoxide. The active Pd(0) species I undergoes oxidative addition into the sulfonyl azide, generating a Pd-bound sulfonyl nitrene intermediate II with the release of nitrogen gas. Coordination of carbon monoxide to intermediate II facilitates migratory insertion, leading to the formation of sulfonyl isocyanate intermediate III. Finally, nucleophilic attack by the indole or pyrrole affords the *N*-acyl sulfonamide product.

More recently, Xia *et al.* demonstrated that C–H aminocarbonylation could be conducted on unactivated alkenes.^89^ Screening of reaction parameters showed that 5 mol% palladium acetate in acetonitrile at 80 °C with a carbon monoxide atmosphere was optimal (Table 38). A range of *N*-acyl sulfonamides were initially prepared by treating alkenes with tosyl azide, affording the corresponding products in moderate to good yields (entries 1–19). Interestingly, aminocarbonylation of an *E*-/*Z*-mixture of 1-ethoxy-1-propene furnished the *E*-isomeric product exclusively in 76% yields (entry 13). Subsequently, the scope of the sulfonyl azide component was investigated by reaction with 3,4-dihydro-2*H*-pyran (entries 20–34). Electron-rich (entries 20–22) and electron-poor (entries 23–26) starting materials displayed good reactivity, as did naphthyl (entries 27 and 28), heteroaryl (entry 29) and aliphatic (entries 31–34) sulfonyl azides. Complex substrates, such as 3,4,6-tri-*O*-benzyl-d-glucal (entry 35) and stigmasterol derivatives (entry 36) produced the corresponding *N*-acyl sulfonamides in good yields. This strategy was also applied to the synthesis of an analogue of glibenclamide (entry 37).^90^

**Table 38:** Pd-catalysed aminocarbonylation of alkenes

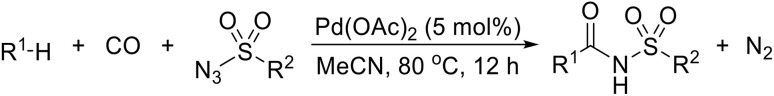			
Entry	R^1^	R^2^	Yield
1	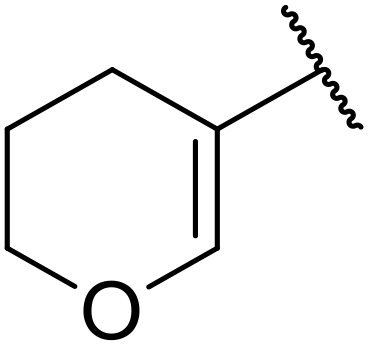	4-MeC_6_H_4_	82%
2	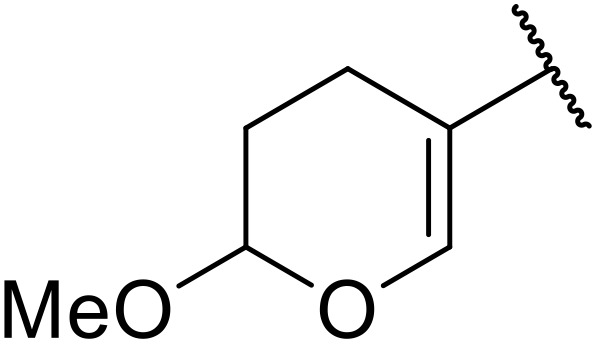	4-MeC_6_H_4_	80%
3	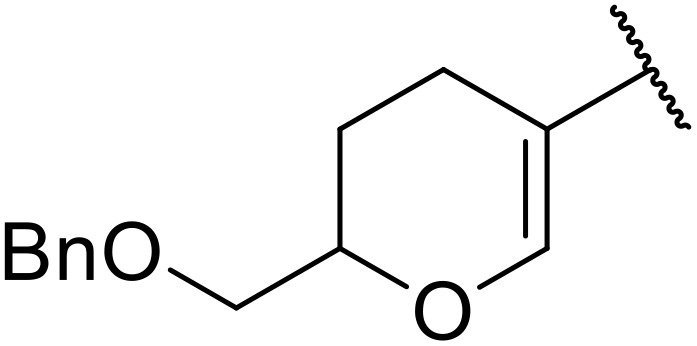	4-MeC_6_H_4_	86%
4	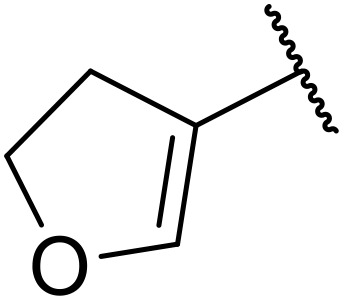	4-MeC_6_H_4_	61%
5	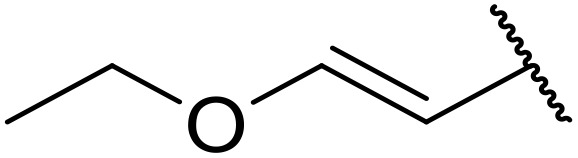	4-MeC_6_H_4_	54%
6	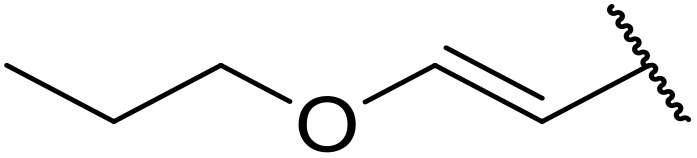	4-MeC_6_H_4_	59%
7	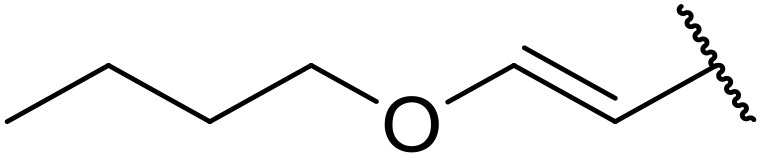	4-MeC_6_H_4_	44%
8	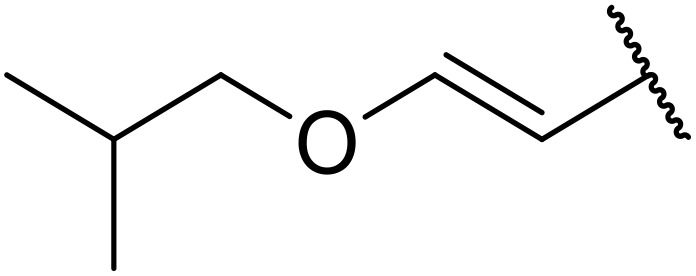	4-MeC_6_H_4_	52%
9	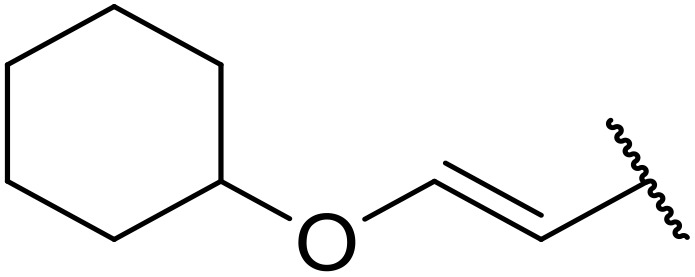	4-MeC_6_H_4_	82%
10	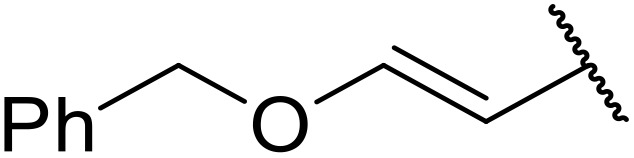	4-MeC_6_H_4_	70%
11	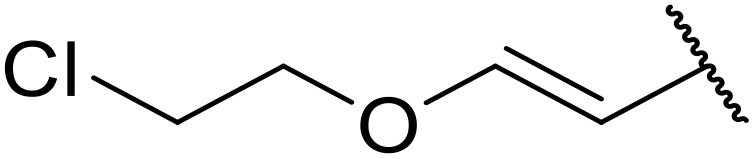	4-MeC_6_H_4_	63%
12	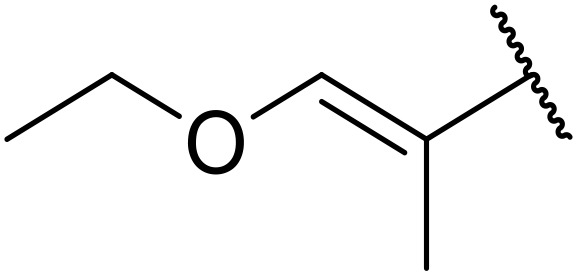	4-MeC_6_H_4_	76%
13	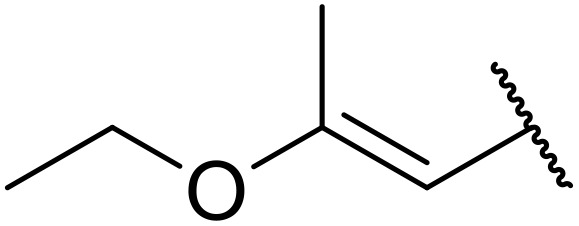	4-MeC_6_H_4_	87%
14	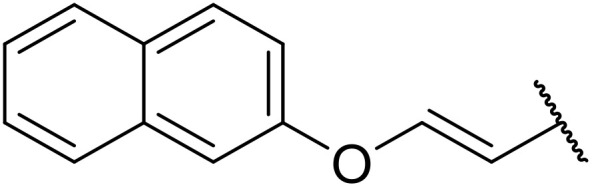	4-MeC_6_H_4_	47%
15	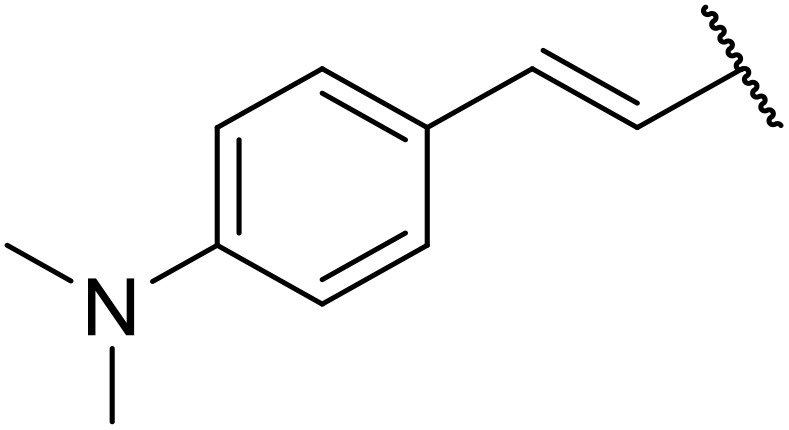	4-MeC_6_H_4_	76%
16	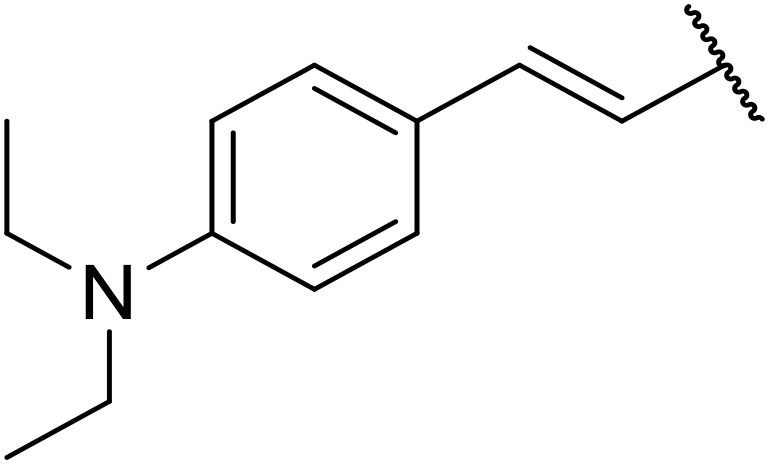	4-MeC_6_H_4_	79%
17	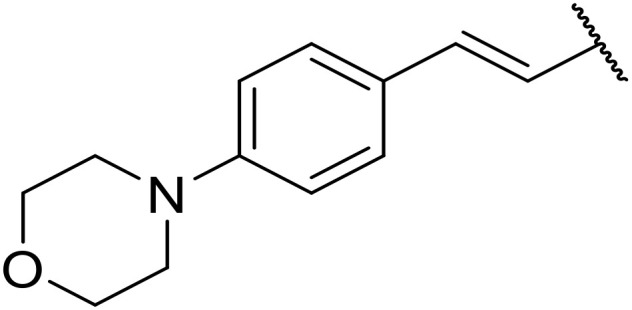	4-MeC_6_H_4_	83%
18	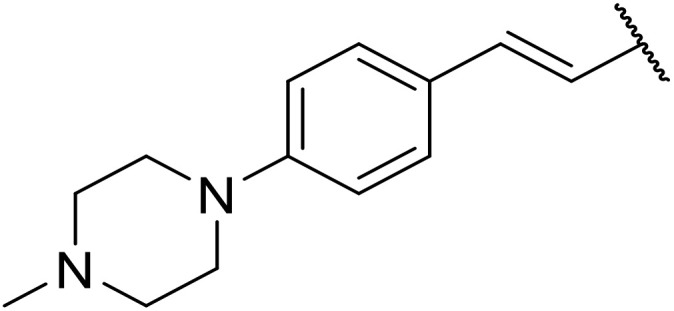	4-MeC_6_H_4_	87%
19	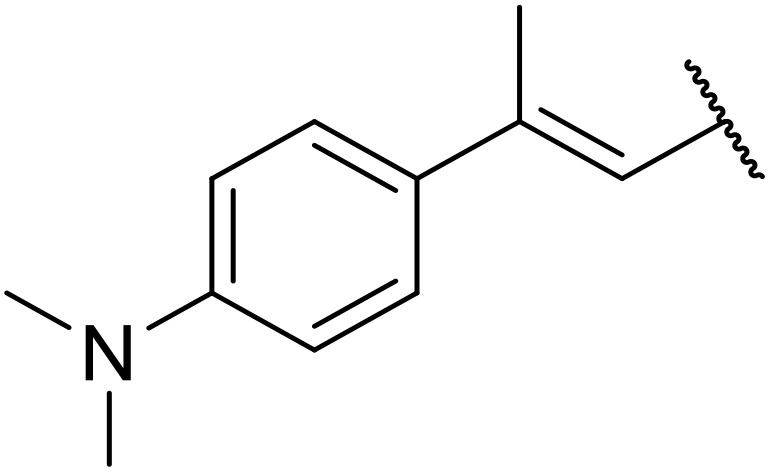	4-MeC_6_H_4_	91%
20	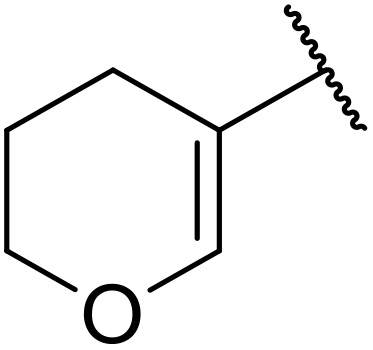	Ph	73%
21	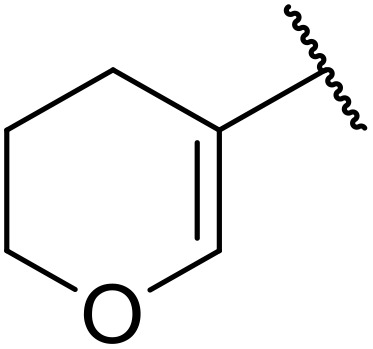	4-MeOC_6_H_4_	82%
22	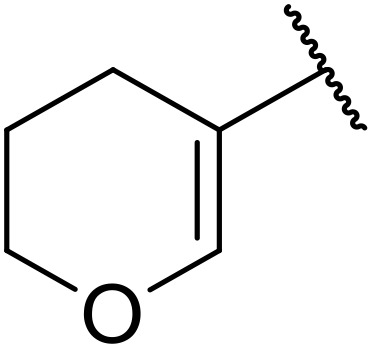	2,4,6-Me_3_C_6_H_2_	81%
23	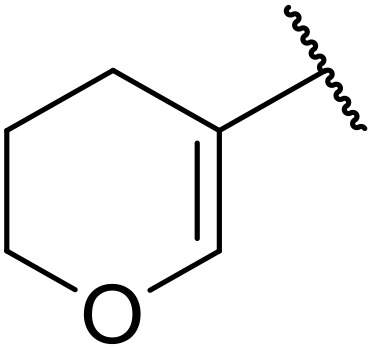	4-F_3_CC_6_H_4_	82%
24	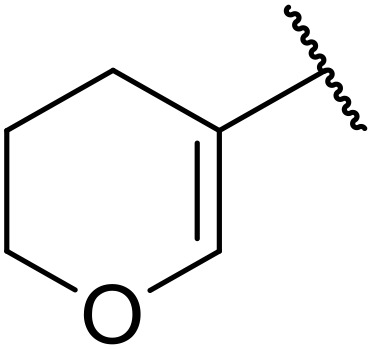	4-FC_6_H_4_	71%
25	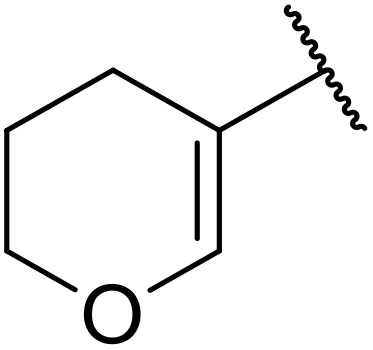	4-ClC_6_H_4_	75%
26	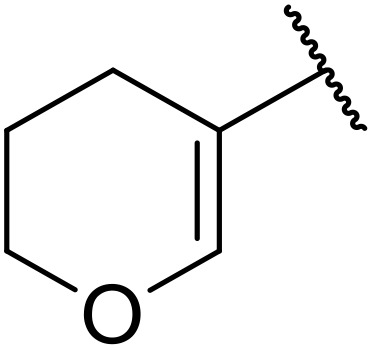	4-BrC_6_H_4_	83%
27	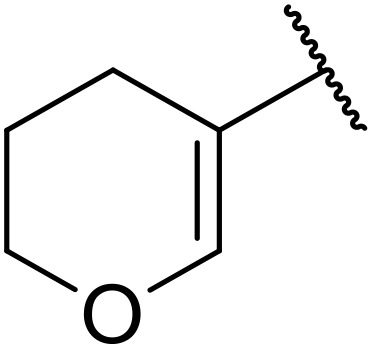	1-Naphthyl	52%
28	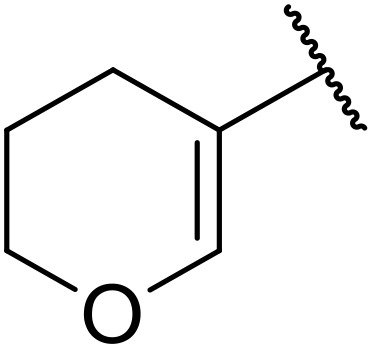	2-Naphthyl	80%
29	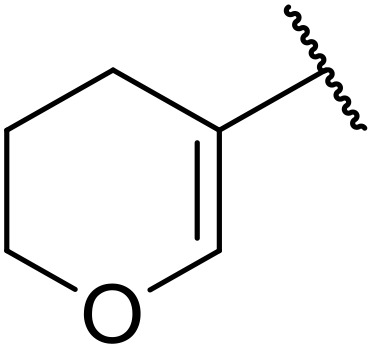	2-Thienyl	65%
30	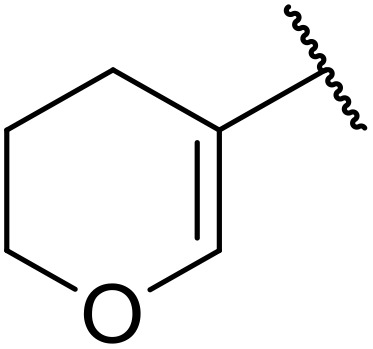	Bn	83%
31	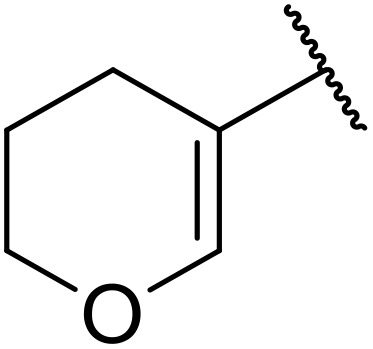	^*n*^Pr	65%
32	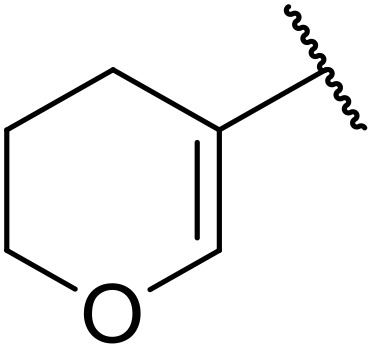	^*n*^Bu	70%
33	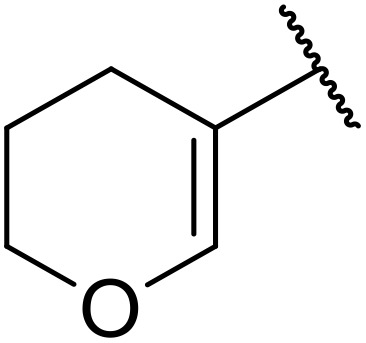	^*i*^Pr	76%
34	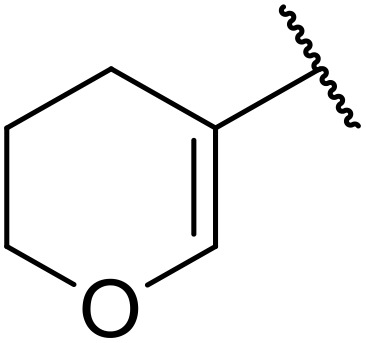	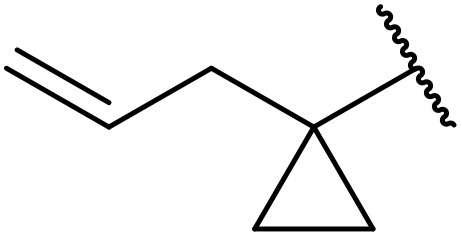	44%
35	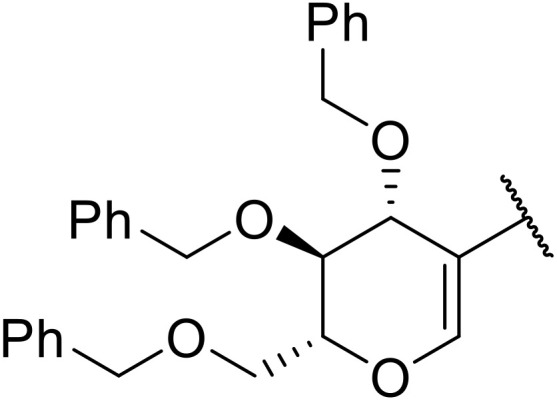	4-MeC_6_H_4_	67%
36	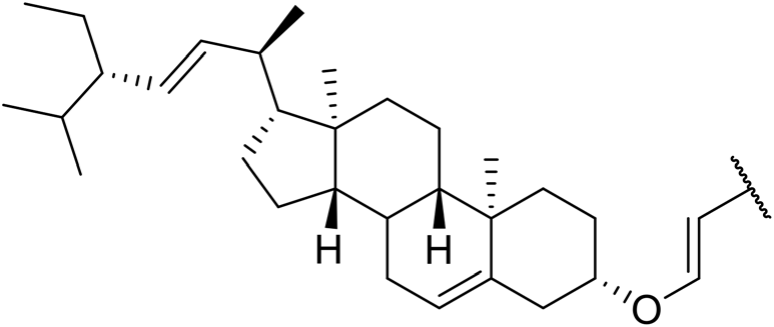	4-MeC_6_H_4_	53%
37	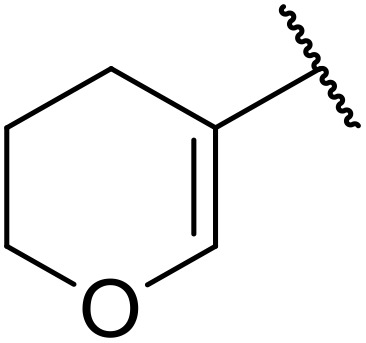	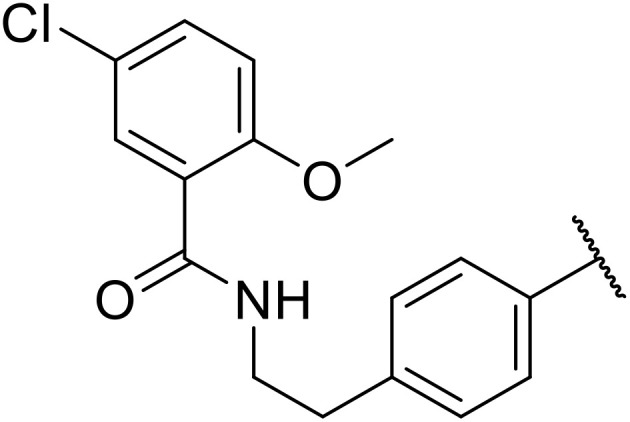	78%

**Scheme 21:**
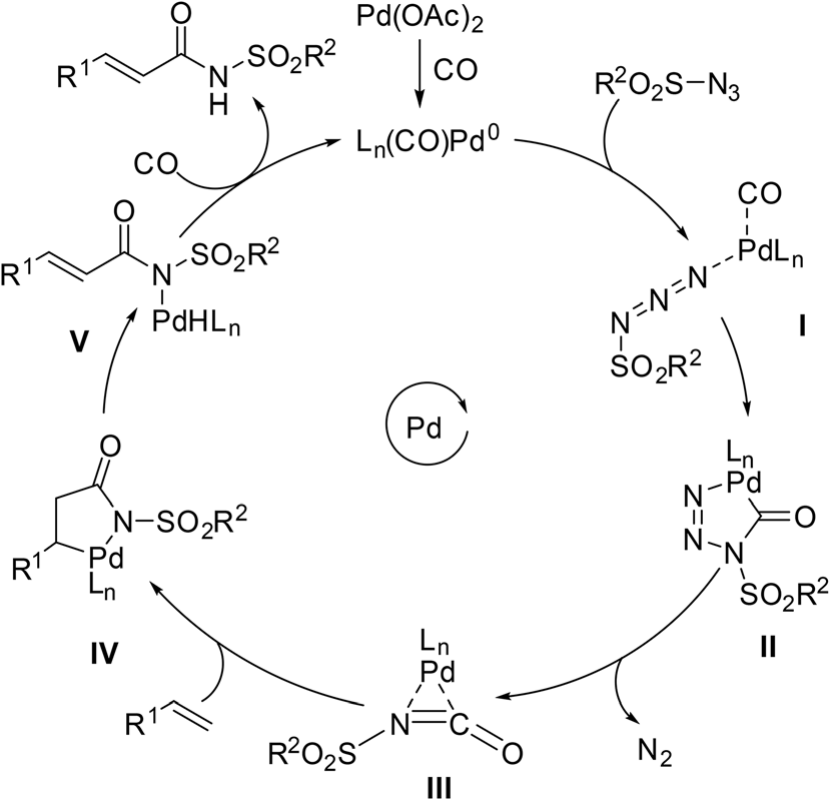
Catalytic cyclic for the aminocarbonylation of alkenes.

The active Pd(0) species is generated by the reaction of palladium acetate with carbon monoxide and is stabilized by an acetonitrile ligand (Scheme 21). This Pd(0) species, containing a coordinated CO ligand, reacts with the sulfonyl azide to generate intermediate I. CO then undergoes migratory insertion into the azide, forming II. The release of nitrogen gas generates palladium-coordinated isocyanate intermediate III. The formation of the isocyanate intermediate was confirmed by a control experiment in which Pd-catalyzed carbonylation of tosyl azide under carbon monoxide furnished tosyl isocyanate. The palladium-coordinated isocyanate intermediate (III) then undergoes alkene coordination, and regioselective migratory insertion affords intermediate IV. This is followed by β-H elimination, leading to the formation of intermediate V. Finally, reductive elimination affords the desired *N*-acyl sulfonamide, while regenerating the Pd(0) catalyst, thereby completing the catalytic cycle.

A Pd-catalysed carbonylation strategy was exploited by Roberts and colleagues to produce acylsulfamides.^91,92^ Two different approaches were developed, using either carbon monoxide, triethylamine and catalytic [1,1′-bis(diphenylphosphino)ferrocene]dichloropalladium(ii) dichloromethane complex [PdCl_2_ (dppf)·CH_2_Cl_2_] (Table 39, method A) or Mo(CO)_6_ and DBU in combination with [Pd(OAc)(P(*o*-tolyl)_3_)]_2_ and [(^*t*^Bu)_3_PH]BF_4_ (method B). The latter method is more advantageous as it does not require access to specialised carbonylation apparatus and is more readily adaptable to parallel synthesis. Using these methods, a range of aryl (entries 1–20) and heteroaryl (21–33) bromides were coupled to pyrrolidine-1-sulfonamide in high yields. Variation of the sulfonamide partner from other cyclic (entries 34–37) to acyclic (entries 38–48) substrates did not negatively impact on outcomes.

**Table 39:** Pd-catalysed carbonylation of sulfamides with aryl/heteroaryl bromides

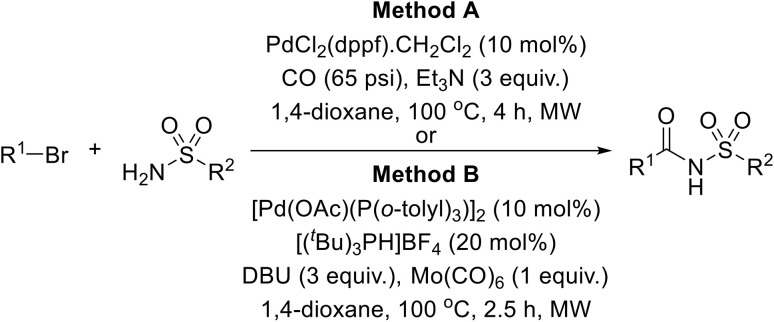				
Entry	R^1^	R^2^	Yield	
			Method A	Method B
1	Ph	Pyrrolidinyl	92%	78%
2	2-MeC_6_H_4_	Pyrrolidinyl	83%	60%
3	3-MeC_6_H_4_	Pyrrolidinyl	83%	72%
4	4-MeC_6_H_4_	Pyrrolidinyl	75%	
5	2-MeOC_6_H_4_	Pyrrolidinyl	73%	68%
6	3-MeOC_6_H_4_	Pyrrolidinyl	82%	88%
7	4-MeOC_6_H_4_	Pyrrolidinyl	80%	88%
8	2-FC_6_H_4_	Pyrrolidinyl	76%	78%
9	2-ClC_6_H_4_	Pyrrolidinyl	61%	56%
10	3-ClC_6_H_4_	Pyrrolidinyl	76%	90%
11	4-ClC_6_H_4_	Pyrrolidinyl	90%	77%
12	2-CyC_6_H_4_	Pyrrolidinyl	36%	
13	4-^*t*^BuC_6_H_4_	Pyrrolidinyl		53%
14	3-MeO_2_CC_6_H_4_	Pyrrolidinyl	70%	
15	4-Me_2_NC(O)C_6_H_4_	Pyrrolidinyl		86%
16	3-NCC_6_H_4_	Pyrrolidinyl	78%	74%
17	4-NCC_6_H_4_	Pyrrolidinyl	80%	50%
18	3-F_3_CC_6_H_4_	Pyrrolidinyl		68%
19	4-F_3_CC_6_H_4_	Pyrrolidinyl		73%
20	2-Naphthyl	Pyrrolidinyl		70%
21	3-Furyl	Pyrrolidinyl		84%
22	2-Thienyl	Pyrrolidinyl	70%	58%
23	3-Thienyl	Pyrrolidinyl	75%	51%
24	2-Pyridyl	Pyrrolidinyl	66%	—
25	3-Pyridyl	Pyrrolidinyl	56%	74%
26	4-Pyridyl	Pyrrolidinyl	58%	46%
27	5-Pyrimidyl	Pyrrolidinyl	78%	
28	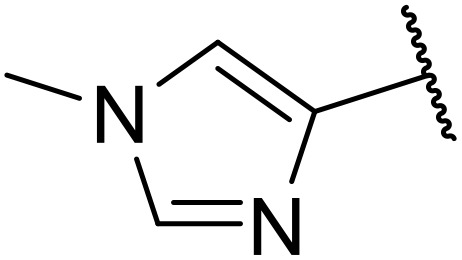	Pyrrolidinyl	50%	
29	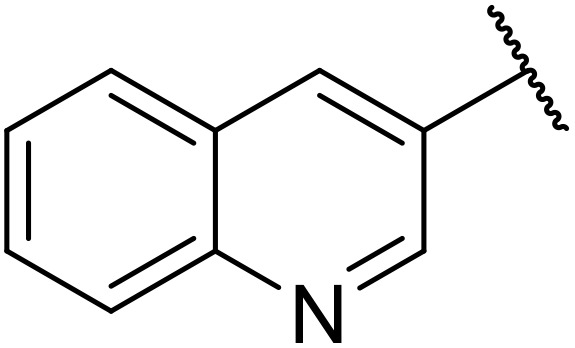	Pyrrolidinyl	86%	76%
30	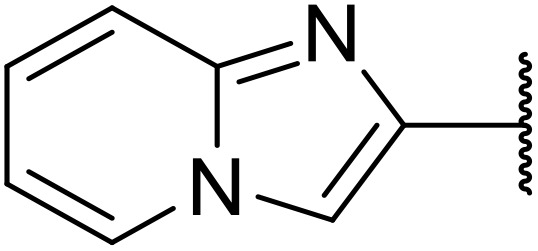	Pyrrolidinyl	72%	
31	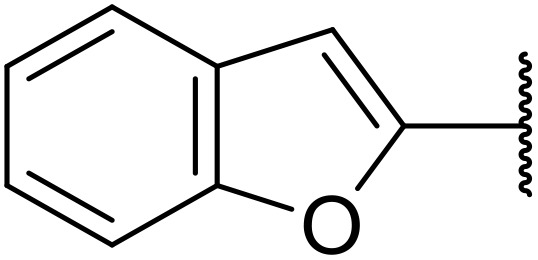	Pyrrolidinyl		52%
32	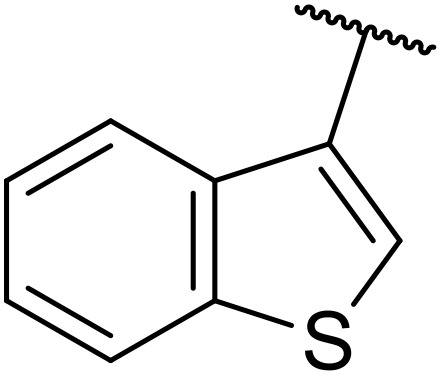	Pyrrolidinyl		87%
33	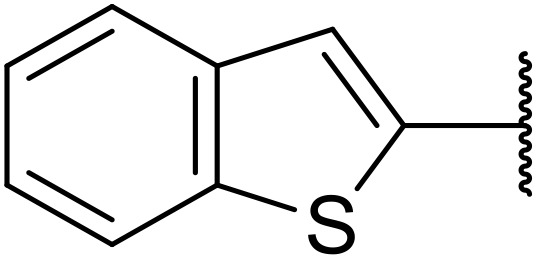	Pyrrolidinyl		55%
34	4-NCC_6_H_4_	Piperidinyl	84%	
35	4-MeOC_6_H_4_	Piperidinyl	66%	
36	4-NCC_6_H_4_	4-Morpholinyl	72%	
37	4-MeOC_6_H_4_	4-Morpholinyl	69%	
38	Ph	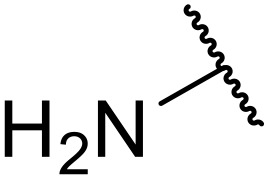	50%	
39	Ph	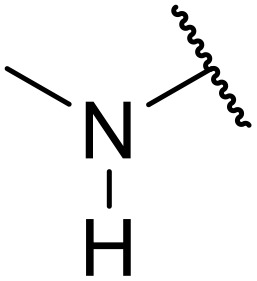	85%	91%
40	4-NCC_6_H_4_	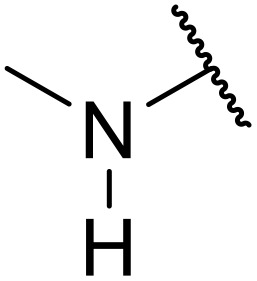	75%	
41	4-NCC_6_H_4_	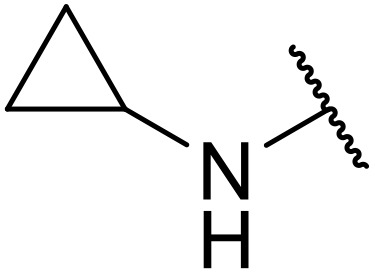	70%	
42	4-NCC_6_H_4_	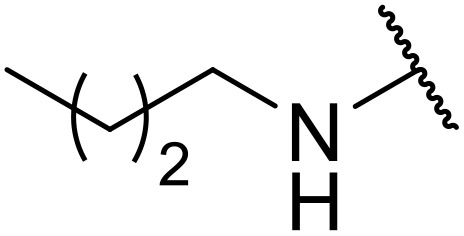	69%	
43	Ph	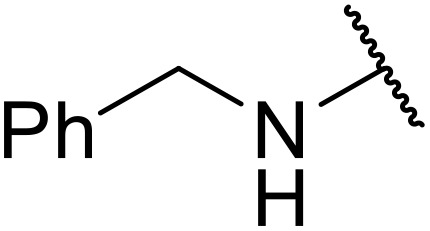		59%
44	4-NCC_6_H_4_	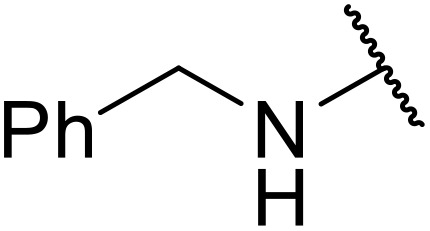	67%	
45	4-MeOC_6_H_4_	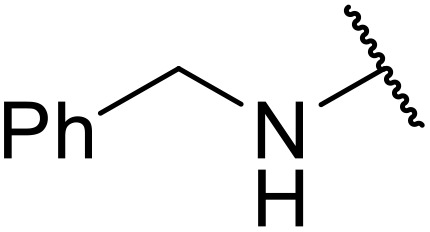	84%	
46	4-NCC_6_H_4_	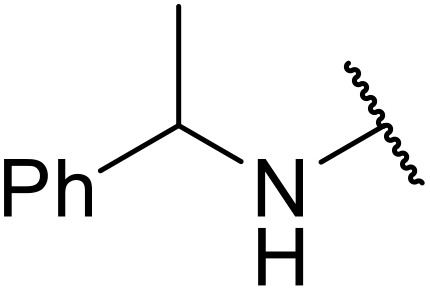	75%	
47	Ph	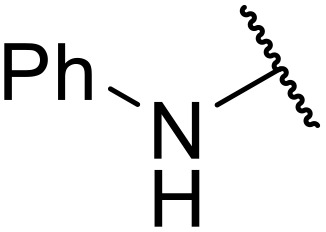		72%
48	4-NCC_6_H_4_	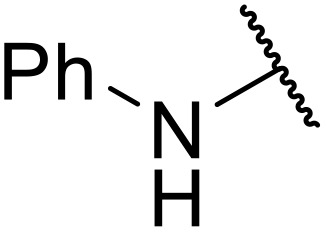	50%	

## 8. Sulfonyl fluorides, chlorides and benzotriazoles

We have previously described the application of the benzotriazole group in the *N*-acylation of sulfonamides (see Table 16). This approach was also adapted to the acylation of amides using the corresponding *N*-sulfonyl benzotriazoles (Table 40).^58^ A small substrate scope study was undertaken where 4-tolyl or 4-pyridyl amides were treated with mesyl or 4-tosyl benzotriazole in the presence of sodium hydride in refluxing THF. The *N*-acyl sulfonamide products were obtained in yields ranging from 34% to 91%, with entries 2–4 comparable to the previous methodology, albeit with longer reaction times. This protocol was extended to α,β-unsaturated amides with the expected *N*-(α,β-unsaturated acyl) sulfonamides recovered in 25–71% yields (entries 5–9).^59^ While these reaction times were relatively short, the average yields were significantly lower than those obtained from the matching *N*-acyl benzotriazoles (see Table 17).

**Table 40:** Coupling of amides and sulfonyl benzotriazoles

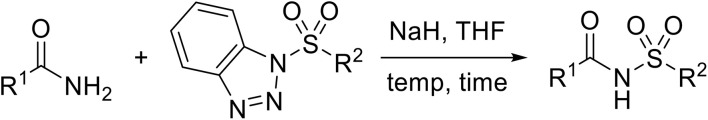					
Entry	R^1^	R^2^	*T* (°C)	Time (h)	Yield
1	4-MeC_6_H_4_	Me	Reflux	24	34%
2	4-MeC_6_H_4_	4-MeC_6_H_4_	Reflux	24	83%
3	4-Pyridyl	Me	Reflux	24	77%
4	4-Pyridyl	4-MeC_6_H_4_	Reflux	24	91%
5	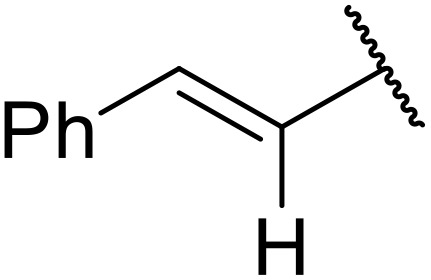	4-MeC_6_H_4_	r.t.	2	71%
6	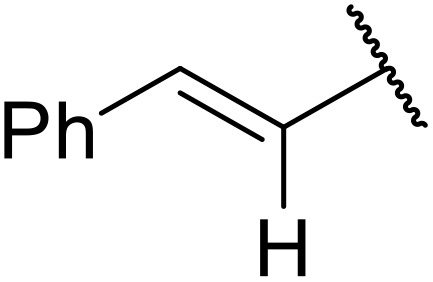	Me	r.t.	2	25%
7	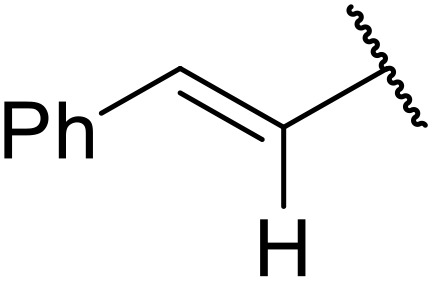	2,4,6-Me_3_C_6_H_2_	r.t.	2	30%
8	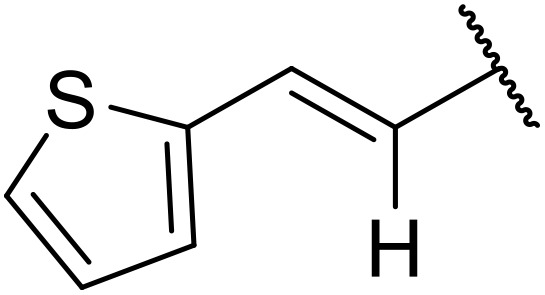	4-MeC_6_H_4_	r.t.	2	30%
9	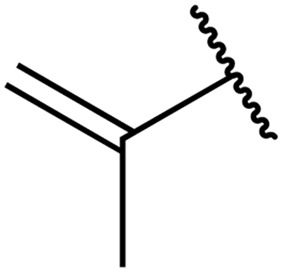	2,4,6-Me_3_C_6_H_2_	r.t.	2	30%

A route to *N*-acyl sulfamates from fluorosulfates was developed by Borggraeve and co-workers.^93^ Fluorosulfates have gained popularity in recent years through Sharpless' sulfur fluoride exchange (SuFEx) chemistry^94^ and in cross coupling reactions.^95^ The protocol demonstrated wide functional group tolerability, with halogen (entries 2, 5, 8 and 10), ester (entry 3) and nitrile (entry 7) substituents withstanding sodium hydride in THF (Table 41). This chemistry was also compatible with more complex natural products (entries 14 and 15). Various amide coupling partners were explored, with butyramide (entry 16), methyl acrylamide (entry 17) and Boc-prolinamide (entry 18) returning similar yields. Unsubstituted (entry 19) and *para*-substituted benzamides (entries 20–22) were less reactive coupling partners, requiring 24 h or elevated temperatures to achieve desirable yields.

**Table 41:** Synthesis of *N*-acyl sulfamates from fluorosulfamates

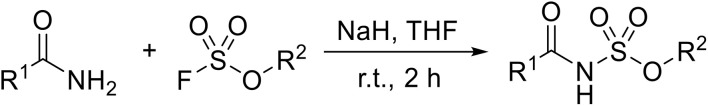			
Entry	R^1^	R^2^	Yield
1	Me	3,5-Me_2_C_6_H_3_	85%
2	Me	3-F_3_CC_6_H_4_	60%
3	Me	4-EtO_2_CC_6_H_4_	70%
4	Me	4-MeOC_6_H_4_	80%
5	Me	3-IC_6_H_4_	68%
6	Me	4-MeSO_2_C_6_H_4_	62%
7	Me	3-NCC_6_H_4_	72%
8	Me	4-BrC_6_H_4_	78%
9	Me	2,6-Me_2_C_6_H_3_	76%
10	Me	2-BrC_6_H_4_	69%
11	Me	2-Naphthyl	83%
12	Me	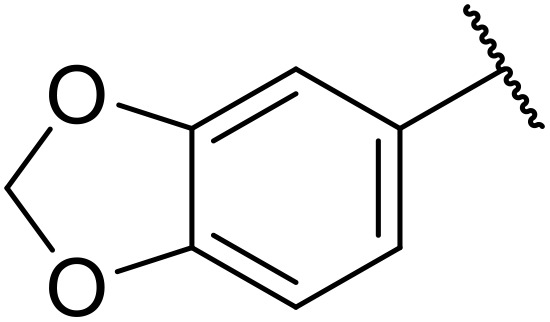	82%
13	Me	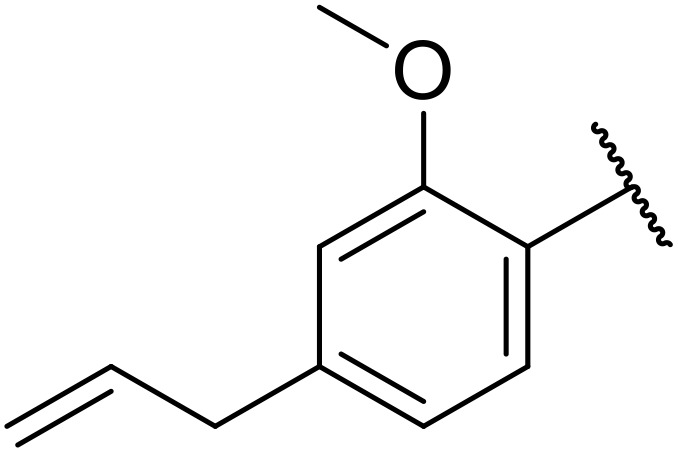	69%
14	Me	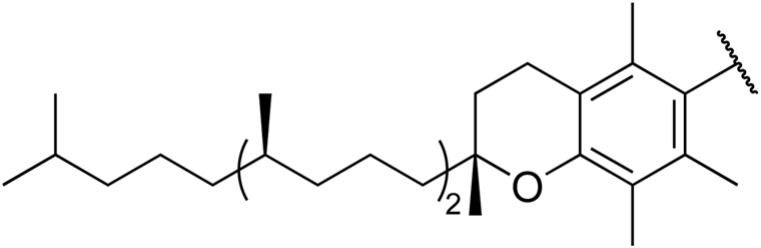	69%
15	Me	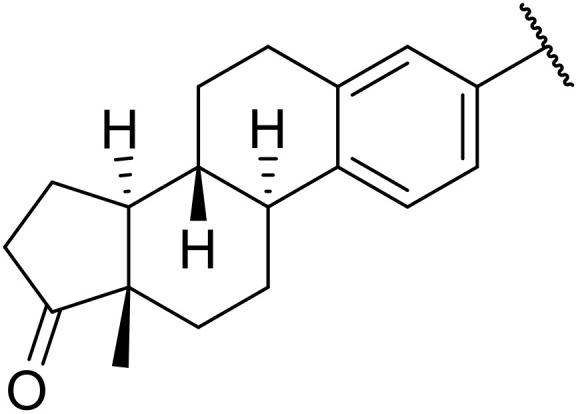	73%
16	^*n*^Pr	3,5-Me_2_C_6_H_3_	69%
17	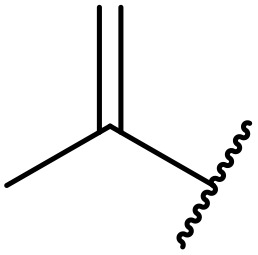	3,5-Me_2_C_6_H_3_	69%
18	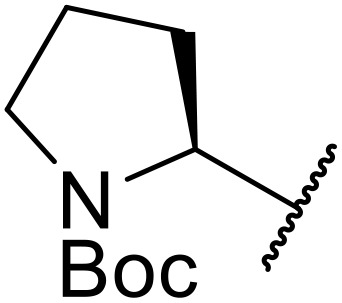	3,5-Me_2_C_6_H_3_	50%
19	Ph	3,5-Me_2_C_6_H_3_	79%^a^
20	4-FC_6_H_4_	3,5-Me_2_C_6_H_3_	62%^a^
21	4-O_2_NC_6_H_4_	3,5-Me_2_C_6_H_3_	63%^a^ (79% at 50 °C)^a^
22	4-MeOC_6_H_4_	3,5-Me_2_C_6_H_3_	25%^a^ (70% at 50 °C)^a^
23	Me	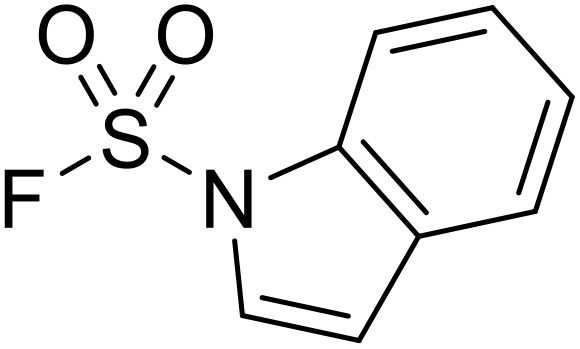	67%

a 24 h.

The unusual reactivity of 4-carboxylamido-2-oxazoline systems was reported by Cossu and co-workers.^96^ The team intended to synthesise 4-nitrile derivatives of various 4-carboxyamido-2-oxazolines using a well-known procedure (*i.e.* reaction with *p*-toluenesulfonyl chloride in pyridine).^97^ However, only the *N*-sulfonylated derivatives were isolated in excellent yields (Table 42). The authors suggest that an equilibrium between two species exists, whereby a hydrogen bond might form between the amidic proton and the carboxylic oxygen. This allows for the amidic nitrogen to attack the electrophilic sulfur, owing to the decreased nucleophilicity of the oxygen atom and is likely due to the presence of α-exocyclic protons (entries 2–5). Notably, racemic 2-phenyl-4-carboxyamido-2-oxazoline treated with *p*-toluenesulfonyl chloride exclusively generated the 4-nitrile derivative, demonstrating the importance of the α-exocyclic proton in this transformation.

**Table 42:** Synthesis of *N*-acyl sulfonamides from 4-carboxylamido-2-oxazoline derivatives

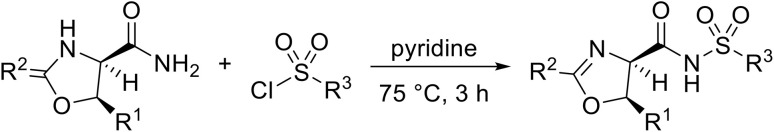				
Entry	R^1^	R^2^	R^3^	Yield
1	Ph	H	4-MeC_6_H_4_	80%
2	Ph(CH_2_)_3_	H	4-MeC_6_H_4_	80%
3	PhCH_2_	H	2-Napthyl	90%
4	PhCH_2_	H	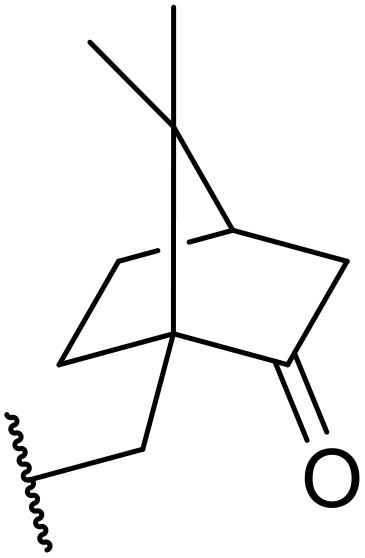	85%
5	Ph(CH_2_)_3_	Me	4-MeC_6_H_4_	85%

**Scheme 22:**
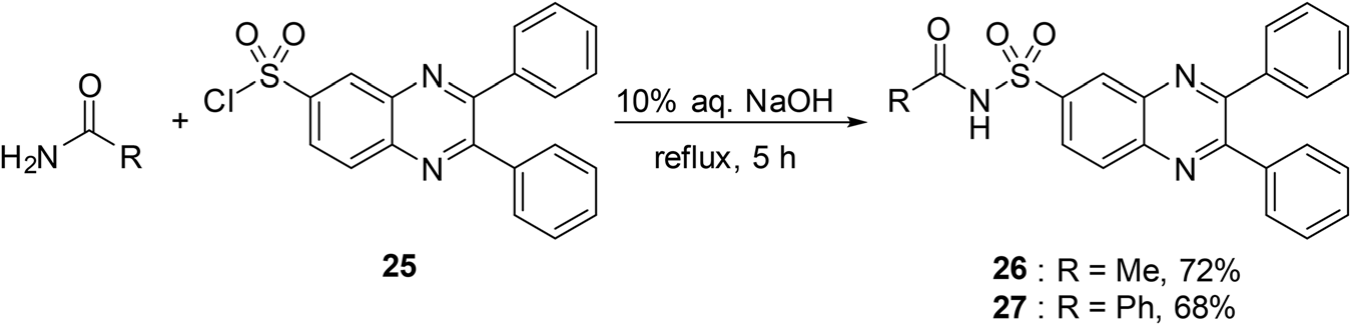
Novel sulfonamide–quinoxaline derivatives.

Patel and co-workers developed a series of novel sulfonamide–quinoxaline derivatives 26 and 27 in their search for anti-cancer agents (Scheme 22).^98^ Different primary amides were refluxed with 2,3-diphenylquinoxaline-6-sulfonyl chloride 25 in 10% aqueous sodium hydroxide solution to afford the *N*-acyl sulfonamides in moderate yields.

**Scheme 23:**
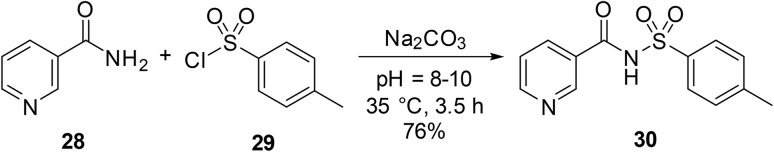
Synthesis of *N*-tosylnicotinamide.

In their investigation of novel antimicrobial agents, Rehman *et al.* treated nicotinamide 28 with tosyl chloride 29 in the presence of sodium carbonate to afford *N*-tosylnicotinamide 30 in 76% yield (Scheme 23).

**Scheme 24:**
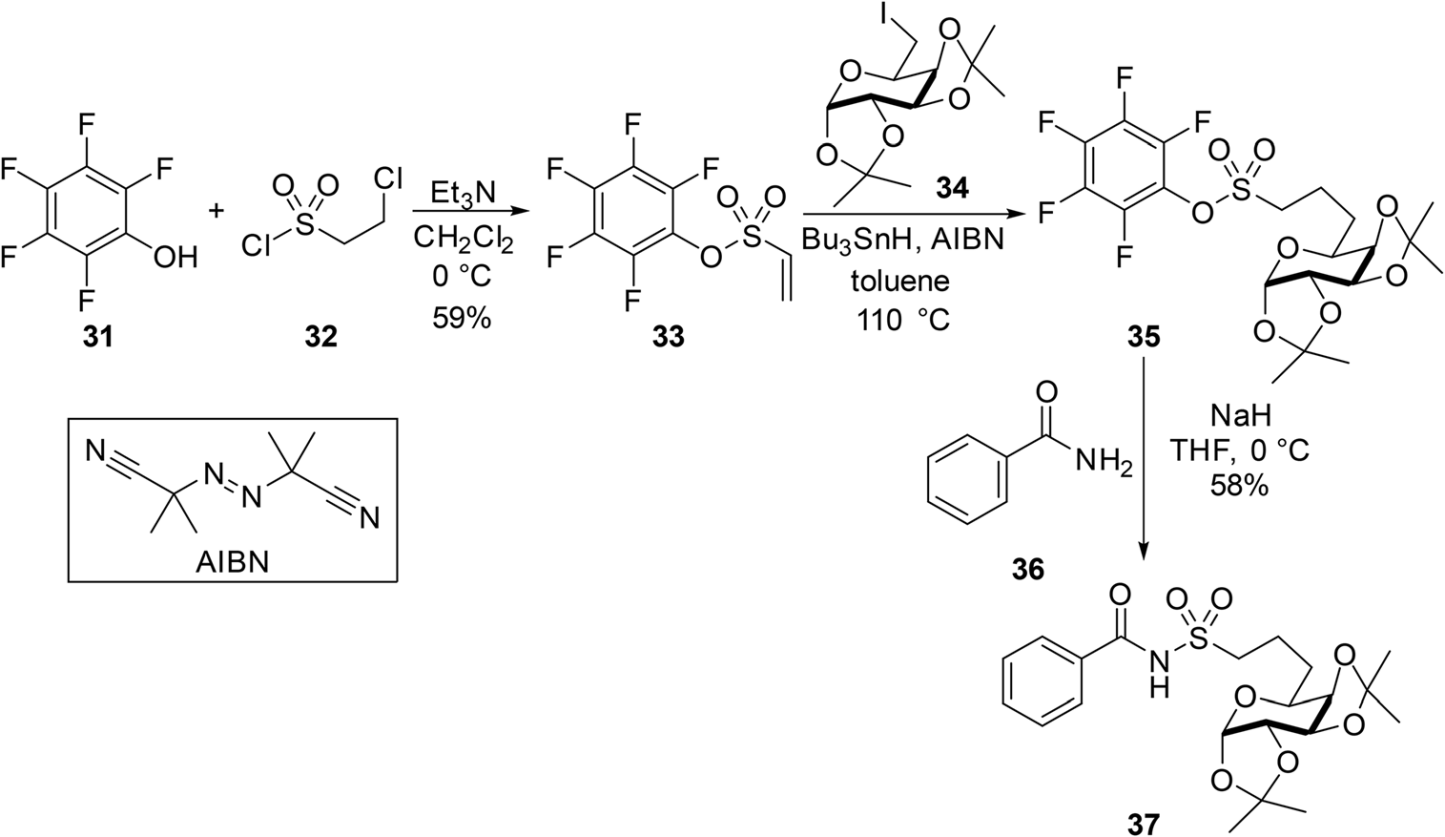
Synthesis of *N*-acyl sulfonamides from pentafluorophenyl vinylsulfonate esters.

Caddick *et al.* established a new route to sulfonamides and *N*-acyl sulfonamides *via* intramolecular radical addition to pentafluorophenyl vinylsulfonate 33 with subsequent aminolysis (Scheme 24).^99^ Initially, pentafluorophenyl vinylsulfonate intermediate 33 was synthesised using pentafluorophenol (31) and 2-chloro-ethane-1-sulfonyl chloride (31). Next, 6-iodo-d-galactose (34) was coupled to pentafluorophenyl vinylsulfonate intermediate 33*via* a tin-mediated radical addition. Subsequent aminolysis of substrate 35 with benzamide 36 and sodium hydride furnished the corresponding *N*-acyl sulfonamide 37. The authors suggest that pentafluorophenyl vinylsulfonate 33 acts as a bifunctional acceptor which is highly susceptible to radical attack, in addition to nucleophilic attack by amines with simultaneous displacement of the pentafluorophenol species. This chemistry has several advantages over sulfonyl chlorides, such as an increased ability to withstand column chromatography or more basic reaction condition.

**Scheme 25:**
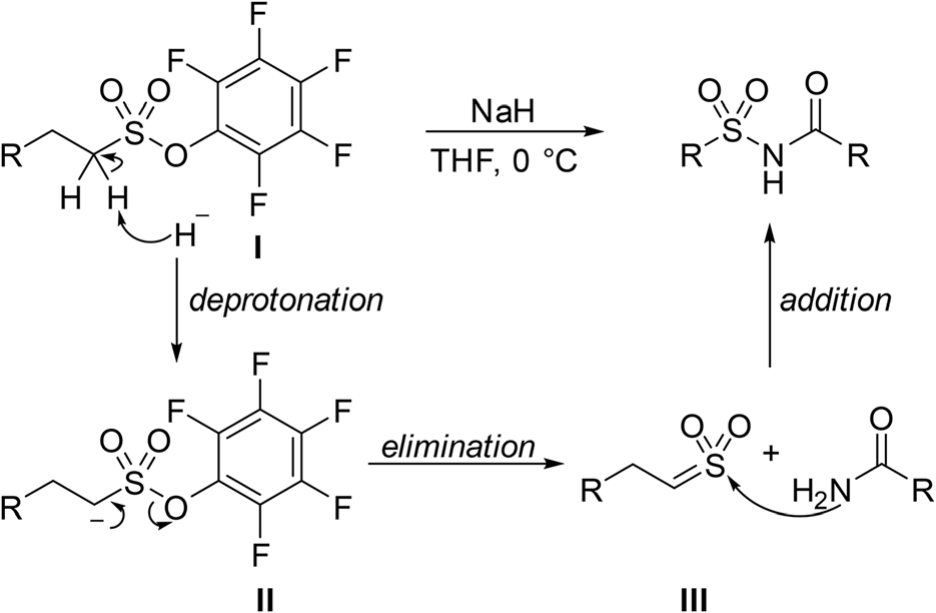
Proposed mechanism for amine displacement.

The authors suggest that the reaction proceeds by the deprotonation of the sulfonate group with rapid extrusion of pentafluorophenol, generating sulfene intermediate III (Scheme 25). This was previously shown by King *et al.* to be the preferred pathway for the hydrolysis of sulfonyl halides at high pH.^100^

## 9. Alkynes

Building upon their previous research on copper-catalysed coupling of sulfonyl azides, alkynes, and amines,^101^ Chang *et al.* later adapted this chemistry to the synthesis of *N*-acyl sulfonamides.^102^ Initial optimisation of the coupling of phenylacetylene and tosyl azide saw isolation of the product in 94% yield using catalytic copper(i) iodide and triethylamine in chloroform (Table 43, entry 1). Other aromatic (entries 2 and 3) and aliphatic (entries 4–10) alkynes reacted in high yields, while functional groups such as alkenes (entry 11), esters (entry 12) and internal alkynes (entry 13) were also tolerated. In the case of propargylic ether (entry 10), swapping triethylamine for lutidine was accompanied by a dramatic increase in yields (34% to 74%). Additionally, phenylacetylene reacted with sulfonyl azides to afford the corresponding acyl sulfonamides in good yields (entries 14–17).

**Table 43:** Copper-catalysed hydrative coupling of terminal alkynes and sulfonyl azides

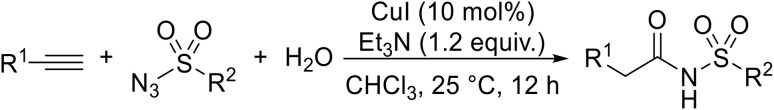			
Entry	R^1^	R^2^	Yield
1	Ph	4-MeC_6_H_4_	94%
2	4-MeC_6_H_4_	4-MeC_6_H_4_	82%
3	4-F_3_CC_6_H_4_	4-MeC_6_H_4_	83%
4	^*n*^Bu	4-MeC_6_H_4_	78%
5	^*t*^Bu	4-MeC_6_H_4_	87%
6	3-Thienyl	4-MeC_6_H_4_	84%
7	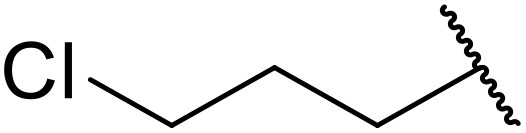	4-MeC_6_H_4_	83%
8	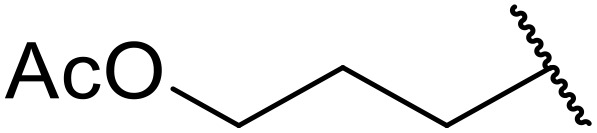	4-MeC_6_H_4_	75%
9	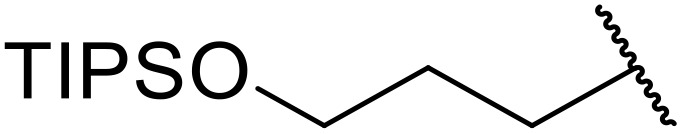	4-MeC_6_H_4_	84%
10	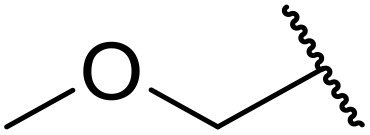	4-MeC_6_H_4_	34% (74%)^a^
11	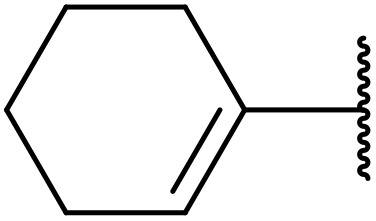	4-MeC_6_H_4_	75%
12	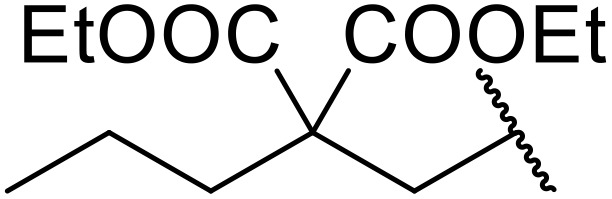	4-MeC_6_H_4_	89%
13	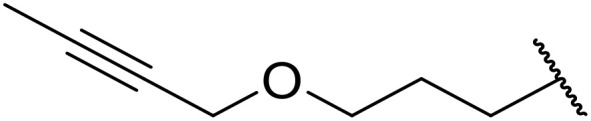	4-MeC_6_H_4_	77%
14	Ph	Bn	74%
15	Ph	^*n*^Bu	87%
16	Ph	(CH_3_)_3_Si(CH_2_)_2_	97%
17	Ph	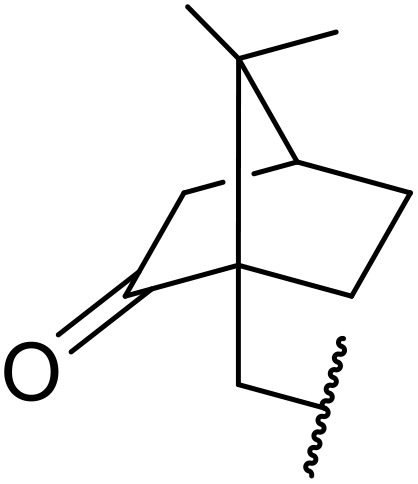	77%
18	BocNHCH_2_	4-MeC_6_H_4_	81%^b^

a Lutidine used in place of triethylamine.

b CuI (3 mol%) was used.

A similar strategy was explored by Fokin *et al.* (Table 44).^103^ A range of alkynes were treated with aryl sulfonyl azides in the presence of 2 mol% [Cu(MeCN)_4_]PF_6_ and tris(benzyltriazolylmethyl)amine (TBTA), sodium ascorbate and sodium bicarbonate in *t*-butanol/water at room temperature. TBTA was found to have an accelerating effect while sodium ascorbate prevented formation of oxidation byproducts. Coupling of 4-acetamidobenzenesulfonyl azide with terminal alkynes bearing alcohol (entry 1), carboxylic acid (entries 2 and 8), carbamate (entry 4) and ether (entry 6) functional groups proceeded in good yields. Transformation of complex substrates, such as ethynyl estradiol (entry 9), was similarly successful.

**Table 44:** Copper(i)-catalysed synthesis of *N*-acyl sulfonamides from alkynes and sulfonyl azides

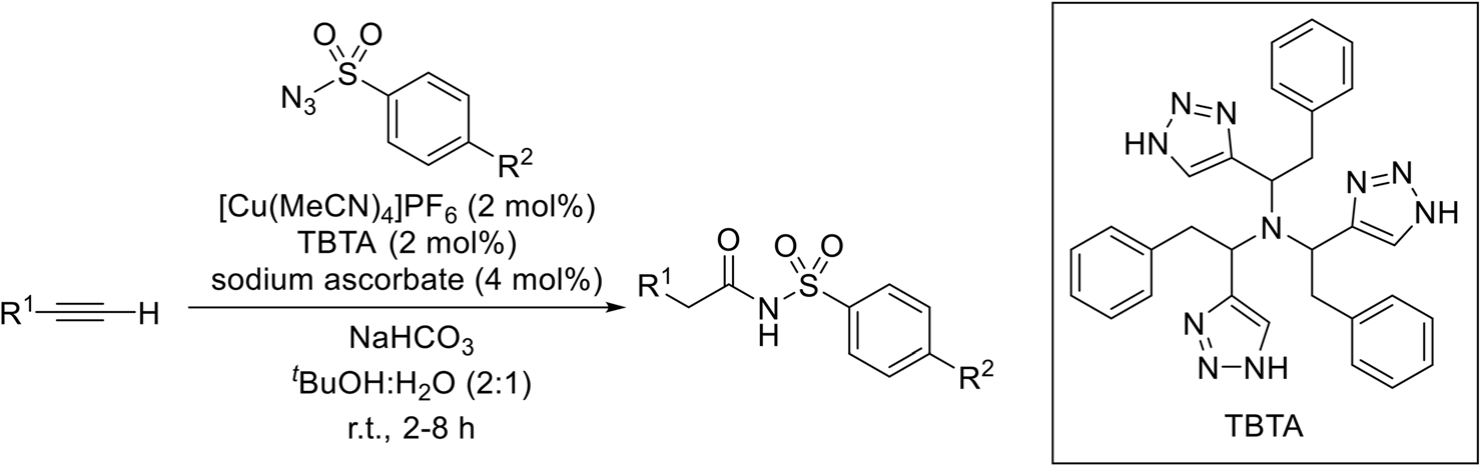			
Entry	R^1^	R^2^	Yield
1	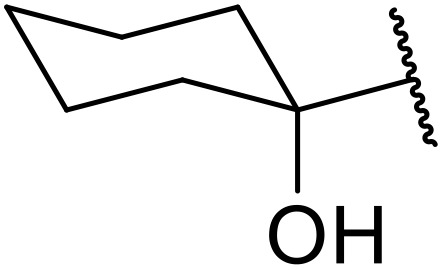	NHAc	60%
2	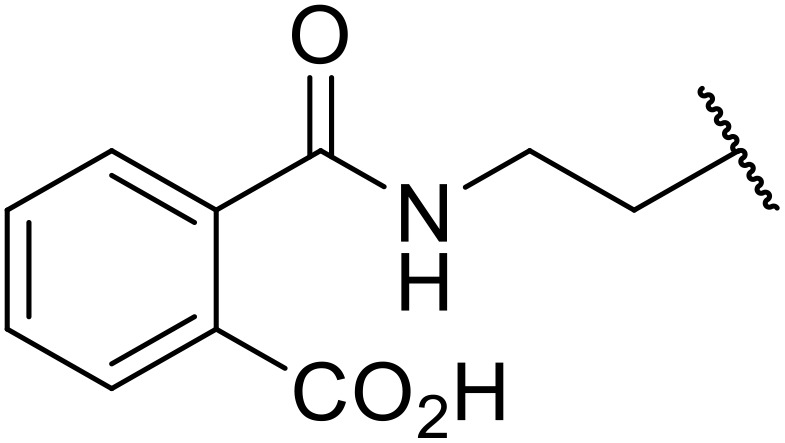	NHAc	67%
3	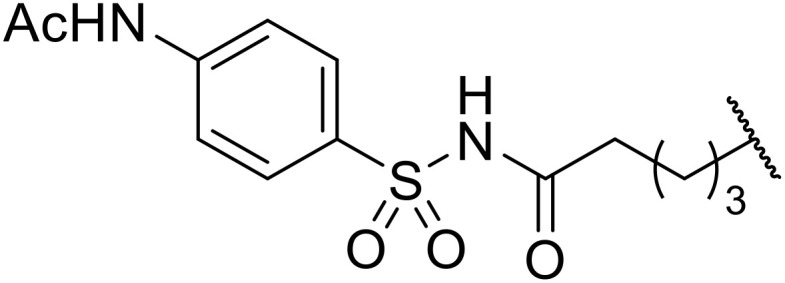	NHAc	74%
4	BocNHCH_2_	NHAc	83%
5	Ph	NHAc	64%
6	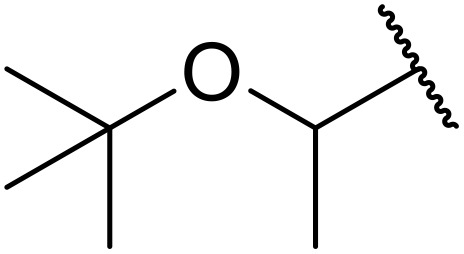	NHAc	83%^a^
7	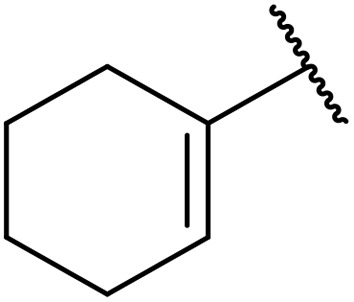	NHAc	54%
8	HO_2_C	NHAc	75%
9	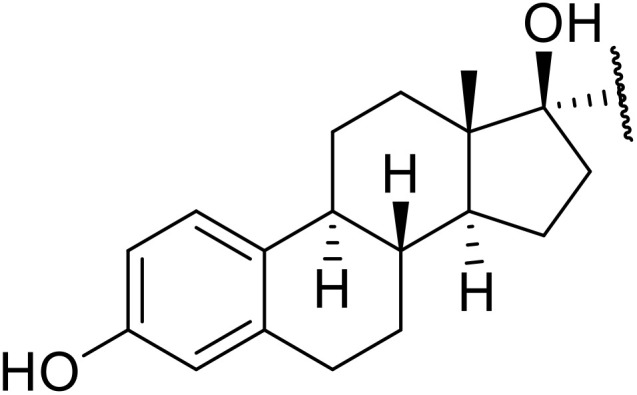	NHAc	68%
10	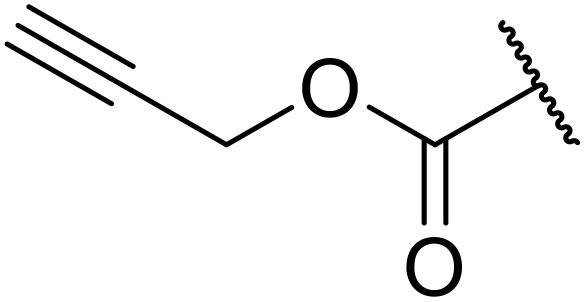	NHAc	62%^a^
11	Ph	NO_2_	27%
12	Ph	Me	69%^a^

a TBTA/[Cu] resin (2 mol%) used as catalyst.

**Scheme 26:**
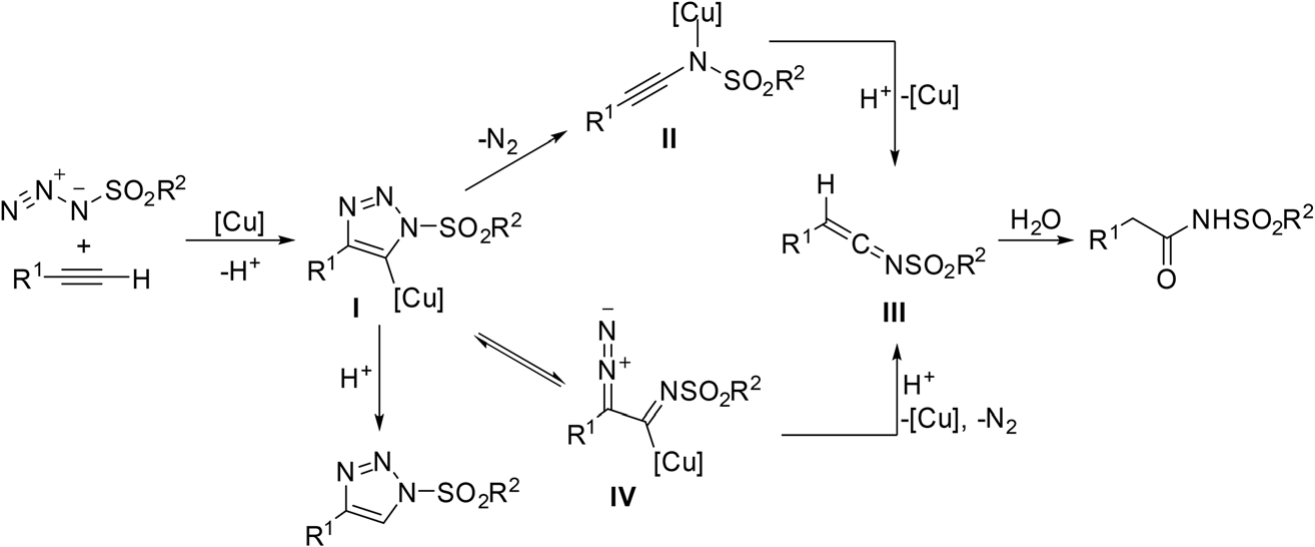
Proposed mechanism for copper-catalysed coupling of alkynes and sulfonyl azides.

Mechanistically, the copper(i) acetylide reacts with a sulfonyl azide to afford (1,2,3-triazol-5-yl)copper intermediate I (Scheme 26). This species undergoes nitrogen elimination to form an alkynamide intermediate II. Protonation of II leads to the formation of a highly reactive ketenimine III, which subsequently undergoes hydrolysis to yield the *N*-acyl sulfonamide. An alternative ring-opening pathway, similar to the Dimroth rearrangement, is also possible where the triazolyl copper species rearranges *via* a diazoimine intermediate IV to form the ketenimine III.

## 10. Conclusion

The presence of an *N*-acyl sulfonamide in wide range of pharmaceutical ingredients and bioactive compounds underlines its significance as a key functional group. The ability to efficiently prepare *N*-acyl sulfonamides remains an important goal in medicinal chemistry. As demonstrated in this review, while traditional routes involving acid chlorides and anhydrides are the mainstay methods to accessing *N*-acyl sulfonamides, an increasing array of novel chemistries are being employed which offer advantages such as increased reactivity, reduced waste or higher selectivity. To our knowledge, no dedicated review on the synthesis of *N*-acyl sulfonamides has been recently published. This review seeks to fill that gap by presenting a comprehensive, reagent-based survey of recent advances in the preparation of *N*-acyl sulfonamide, including assessments of reaction scope, functional group tolerance, and potential for late-stage diversification. This review should act as practical resource for both synthetic chemists and drug discovery teams, facilitating the informed selection of the most efficient routes to this important functional group.

## Conflicts of interest

There are no conflicts of interest to declare.

## Data Availability

No primary research results, software or code have been included and no new data were generated or analysed as part of this review.
